# Supplementary material for: Elucidation of unusual biosynthesis and DnaN-targeting mode of action of potent anti-tuberculosis antibiotics Mycoplanecins
Source: Nat Commun. 2024 Jan 26;15:791. doi: 10.1038/s41467-024-44953-5 (PMC10817943; doi:10.1038/s41467-024-44953-5)
Supplement: Supplementary file 1 — Supplementary Information [file 41467_2024_44953_MOESM1_ESM.pdf]

Supplementary Information

Elucidation of unusual biosynthesis and DnaN-targeting mode of action of potent anti-tuberculosis antibiotics Mycoplanecins

Chengzhang Fu[1,2,#], Yunkun Liu[1,#], Christine Walt[1,4,#], Sari Rasheed[1,4,#], Chantal D. Bader[1,4], Peer Lukat[3], Markus Neuber[1,4], F.P. Jake Haeckl[1,4], Wulf Blankenfeldt[3], Olga V. Kalinina[5,6], Rolf Müller[1,2,4,*]

[1] Helmholtz Institute for Pharmaceutical Research Saarland (HIPS), Helmholtz Centre for Infection Research (HZI), and Department of Pharmacy, Saarland University, 66123 Saarbrücken, Germany

[2] Helmholtz International Lab for Anti-Infectives, Helmholtz Center for Infection Research, 38124 Braunschweig, Germany

[3] Structure and Function of Proteins, Helmholtz Centre for Infection Research, Inhoffenstr. 7, 38124, Braunschweig, Germany.

[4] German Centre for Infection Research (DZIF), 38124 Braunschweig, Germany

[5] Medical Faculty, Saarland University, 66421 Homburg, Germany

[6] Helmholtz Institute for Pharmaceutical Research Saarland (HIPS), Helmholtz Centre for Infection Research (HZI), and Center for Bioinformatics, Saarland Informatics Campus, 66123 Saarbrücken, Germany

# Equal contribution

* Corresponding author: Rolf Müller

Email: rolf.mueller@helmholtz-hips.de

Keywords

Mycoplanecin, DnaN, tuberculosis, alkylproline, homo-amino acid biosynthesis

Supplementary Discussion 7

Coexistence of self-resistance gene and putative transposase genes in the MP BGC 7

Validation of the *mypA* gene sequence.. 7

The α-ketobutyric acid moiety formation in MP biosynthesis 7

Marfey's analysis 8

Crystal structure of DnaN in complex with MP A 8

Supplementary Figures... 9

Supplementary Figure 1. Dot plots of two versions f mypA genes.. 9

Supplementary Figure 2. Design of a combination of PCR amplification to verify the mypA gene sequence. 10

Supplementary Figure 3. PCR amplified fragments from the genomic DNA of *A. awajinensis* subsp. *mycoplanecinus* subsp. nov. ATCC 33919 using a combination of primers confirm the mypA gene should be the 22.9 kb version. 11

Supplementary Figure 4. MST dose-response curves for the binding interaction between BLUE-NHS labeled MsDnaN and different ligands.. 12

Supplementary Figure 5. Comparison of *E. coli* and mycobacterial DnaNs................................ 13

Supplementary Figure 6. Electron density maps 14

Supplementary Figure 7. Ligand interaction diagrams. 15

Supplementary Figure 8. Conformational changes upon ligand binding. 16

Supplementary Figure 9. Binding pose comparison of MP A and GM in the ecDnaN complexes with GM bound to mycobacterial DnaNs.. 16

Supplementary Figure 10. Relative crystallographic B-factors of MP A and GM in the complexes ecDnaN and the mycobacterial proteins... 17

Supplementary Figure 11. *In vitro* transamination of purified MypL$_C$. 18

Supplementary Figure 12. SDS-PAGE gel of recombinant proteins involved in this study......... 19

Supplementary Figure 13. Biosynthetic routes to 4-EtPro and 4-PrPro in lincomycins biosynthesis 19

Supplementary Figure 14. Examples of natural products containing 4-ethylproline, 4-propylproline and homoleucine.. 20

Supplementary Figure 15. 1H-spectrum of MP A in CDCl3 at 500 MHz. 21

Supplementary Figure 16. ^{13}C-spectrum of MP A in $CDCl_3$ at 125 MHz.................................... 22

Supplementary Figure 17. COSY-spectrum of MP A in CDCl3 at 500 MHz. 23

Supplementary Figure 18. HSQC-spectrum of MP A in $CDCl_3$ at 500 MHz (^{1}H)/125 MHz (^{13}C). 24

Supplementary Figure 19. HMBC-spectrum of MP A in CDCl3 at 500 MHz (1H)/125 MHz (13C).. 25

Supplementary Figure 20. TOCSY-spectrum of MP A in CDCl3 at 500 MHz. 26

Supplementary Figure 21. Marfey's derivatization of reference N-Methyl-L-Valine (upper chromatogram) with D-FDLA and MP A with both D-FDLA (middle chromatogram) and L-FDLA (lower chromatogram)... 27

Supplementary Figure 22. Marfey's derivatization of reference R-Ethyl-S-Proline (upper chromatogram) with D-FDLA and MP A with both D-FDLA (middle chromatogram) and L-FDLA (lower chromatogram)... 27

Supplementary Figure 23. Marfey's derivatization of reference L-Leucine (upper chromatogram) with D-FDLA and MP A with both D-FDLA (middle chromatogram) and L-FDLA (lower chromatogram). 28

Supplementary Figure 24. Marfey's derivatization of reference R-Methyl-S-Proline (upper chromatogram) with D-FDLA and MP A with both D-FDLA (middle chromatogram) and L-FDLA (lower chromatogram)... 28

Supplementary Figure 25. Marfey's derivatization of reference 5-Methyl-D/L-Norleucine (upper chromatogram) with D-FDLA and MP A with both D-FDLA (middle chromatogram) and L-FDLA (lower chromatogram)... 29

Supplementary Figure 26. Marfey's derivatization of reference L-Proline (upper chromatogram) with D-FDLA and MP A with both D-FDLA (middle chromatogram) and L-FDLA (lower chromatogram). 29

Supplementary Figure 27. Marfey's derivatization of reference N-Methyl-L-Leucine with D-FDLA (upper chromatogram) and MP A with both D-FDLA (middle chromatogram) and L-FDLA (lower chromatogram)... 30

Supplementary Figure 28. Marfey's derivatization of reference N-Methyl-L-Threonine with D-FDLA (upper chromatogram) and MP A with both D-FDLA (middle chromatogram) and L-FDLA (lower chromatogram)... 30

Supplementary Figure 29. ^{1}H-spectrum of MP B in $CDCl_3$ at 500 MHz. 31

Supplementary Figure 30. 13C-spectrum of MP B in CDCl3 at 125 MHz................................. 32

Supplementary Figure 31. COSY-spectrum of MP B in CDCl3 at 500 MHz. 33

Supplementary Figure 32. HSQC-spectrum of MP B in $CDCl_3$ at 500 MHz (^{1}H)/125 MHz (^{13}C). 34

Supplementary Figure 33. HMBC-spectrum of MP B in $CDCl_3$ at 500 MHz (^{1}H)/125 MHz (^{13}C). 35

Supplementary Figure 34. TOCSY-spectrum of MP B in $CDCl_3$ at 500 MHz. 36

Supplementary Figure 35. Marfey's derivatization of reference N-Methyl-L-Valine with D-FDLA (upper chromatogram) and MP B with both D-FDLA (middle chromatogram) and L-FDLA (lower chromatogram). 37

Supplementary Figure 36. Marfey's derivatization of reference R-Methyl-S-Proline with D-FDLA (upper chromatogram) and MP B with both D-FDLA (middle chromatogram) and L-FDLA (lower chromatogram)... 37

Supplementary Figure 37. Marfey's derivatization of reference L-Leucine with D-FDLA (upper chromatogram) and MP B with both D-FDLA (middle chromatogram) and L-FDLA (lower chromatogram). 38

Supplementary Figure 38. Marfey's derivatization of reference 5-Methyl-D/L-Norleucine with D-FDLA (upper chromatogram) and MP B with both D-FDLA (middle chromatogram) and L-FDLA (lower chromatogram).. 38

Supplementary Figure 39. Marfey's derivatization of reference L-Proline with D-FDLA (upper chromatogram) and MP B with both D-FDLA (middle chromatogram) and L-FDLA (lower chromatogram). 39

Supplementary Figure 40. Marfey's derivatization of reference N-Methyl-L-Leucine with D-FDLA (upper chromatogram) and MP B with both D-FDLA (middle chromatogram) and L-FDLA (lower chromatogram).. 39

Supplementary Figure 41. Marfey's derivatization of reference N-Methyl-L-Threonine with D-FDLA (upper chromatogram) and MP B with both D-FDLA (middle chromatogram) and L-FDLA (lower chromatogram).. 40

Supplementary Figure 42. ^{1}H-spectrum of MP D in $CDCl_3$ at 500 MHz.................................... 41

Supplementary Figure 43. ^{13}C-spectrum of MP D in $CDCl_3$ at 125 MHz.................................... 42

Supplementary Figure 44. COSY-spectrum of MP D in $CDCl_3$ at 500 MHz. 43

Supplementary Figure 45. HSQC-spectrum of MP D in CDCl3 at 500 MHz (1H)/125 MHz (13C).. 44

Supplementary Figure 46. HMBC-spectrum of MP D in CDCl3 at 500 MHz (1H)/125 MHz (13C).. 45

Supplementary Figure 47. TOCSY-spectrum of MP D in $CDCl_3$ at 500 MHz............................ 46

Supplementary Figure 48. Marfey's derivatization of reference N-Methyl-L-Valine (upper chromatogram) with D-FDLA and MP D with both D-FDLA (middle chromatogram) and L-FDLA (lower chromatogram).. 47

Supplementary Figure 49. Marfey's derivatization of MP D with D-FDLA. 47

Supplementary Figure 50. Marfey's derivatization of reference L-Leucine with D-FDLA (upper chromatogram) and MP D with both D-FDLA (middle chromatogram) and L-FDLA (lower chromatogram). 48

Supplementary Figure 51. Marfey's derivatization of reference R-Methyl-S-Proline (upper chromatogram) with D-FDLA and MP D with both D-FDLA (middle chromatogram) and L-FDLA (lower chromatogram).. 48

Supplementary Figure 52. Marfey's derivatization of reference 5-Methyl-D/L-Norleucine with D-FDLA (upper chromatogram) and MP D with both D-FDLA (middle chromatogram) and L-FDLA (lower chromatogram).. 49

Supplementary Figure 53. Marfey's derivatization of reference L-Proline (upper chromatogram) with D-FDLA and MP D with both D-FDLA (middle chromatogram) and L-FDLA (lower chromatogram). 49

Supplementary Figure 54. Marfey's derivatization of reference N-Methyl-L-Leucine with D-FDLA (upper chromatogram) and MP D with both D-FDLA (middle chromatogram) and L-FDLA (lower chromatogram)........ 50

Supplementary Figure 55. Marfey's derivatization of reference N-Methyl-L-Threonine with D-FDLA (upper chromatogram) and MP D with both D-FDLA (middle chromatogram) and L-FDLA (lower chromatogram)........ 50

Supplementary Figure 56. 1H-spectrum of MP E in CDCl3 at 700 MHz........ 51

Supplementary Figure 57. COSY-spectrum of MP E in CDCl3 at 700 MHz........ 52

Supplementary Figure 58. HSQC-spectrum of MP E in CDCl3 at 700 MHz (1H)/175 MHz (13C). 53

Supplementary Figure 59. HMBC-spectrum of MP E in CDCl3 at 700 MHz (1H)/175 MHz (13C)........ 54

Supplementary Figure 60. Marfey's derivatization of reference N-Methyl-L-Valine (upper chromatogram) with D-FDLA and MP E with both D-FDLA (middle chromatogram) and L-FDLA (lower chromatogram)........ 55

Supplementary Figure 61. Marfey's derivatization of MP E with D-FDLA........ 55

Supplementary Figure 62. Marfey's derivatization of reference L-Leucine with D-FDLA (upper chromatogram) and MP E with both D-FDLA (middle chromatogram) and L-FDLA (lower chromatogram). 56

Supplementary Figure 63. Marfey's derivatization of reference R-Methyl-S-Proline (upper chromatogram) with D-FDLA and MP E with both D-FDLA (middle chromatogram) and L-FDLA (lower chromatogram)........ 56

Supplementary Figure 64. Marfey's derivatization of reference 5-Methyl-D/L-Norleucine with D-FDLA (upper chromatogram) and MP E with both D-FDLA (middle chromatogram) and L-FDLA (lower chromatogram)........ 57

Supplementary Figure 65. Marfey's derivatization of reference L-Proline (upper chromatogram) with D-FDLA and MP E with both D-FDLA (middle chromatogram) and L-FDLA (lower chromatogram). 57

Supplementary Figure 66. Marfey's derivatization of MP E with D-FDLA measured with a more apolar gradient........ 58

Supplementary Figure 67. Marfey's derivatization of reference N-Methyl-L-Threonine (upper chromatogram) with D-FDLA and MP E with both D-FDLA (middle chromatogram) and L-FDLA (lower chromatogram)........ 58

Supplementary Tables........ 59

Supplementary Table 1. All homologs of GriE and GriF found in biosynthetic gene clusters (BGCs) in all species of the phylum *Actinobacteria* which were used in sequence similarity networks in this study........ 59

Supplementary Table 2. Deduced functions of genes in the mycoplanecins biosynthetic gene cluster (genbank ccession number OR083095) from Actinoplanes awajinensis ATCC 33919.... 62

Supplementary Table 3. Substrate specificity analysis of the 11 adenylation (A) domains from the mycoplanecins megasynthetase........ 63

Supplementary Table 4. Analysis of active site, acyl-transfer and C-O bond formation region residues of MypJ homologues. 63

Supplementary Table 5. Walking primers used for the verification of MP gene cluster. 63

Supplementary Table 6. The comparison of bioactivity of MPs and GM against different bacteria 64

Supplementary Table 7. Primers used for protein expression. 64

Supplementary Table 8. X-ray data collection and refinement statistics. 65

Supplementary Table 9. Crystallization conditions 66

Supplementary Table 10. Kinetic data for the enzymatic condensation MypK substrates 66

Supplementary Table 11. NMR spectroscopic data of MP A in CDCl3 at 500/125 MHz. 67

Supplementary Table 12. NMR spectroscopic data of MP B in CDCl3 at 500/125 MHz. 71

Supplementary Table 13. NMR spectroscopic data of MP D in CDCl3 at 500/125 MHz. 75

Supplementary Table 14. NMR spectroscopic data of MP E in CDCl3 at 700/175 MHz. 78

References 81

Supplementary Discussion

Coexistence of self-resistance gene and putative transposase genes in the MP BGC

Three putative transposase genes (*tnp1, tnp2 and tnp3*) were identified in the 5'-end of the MP BGC. It was also observed in the GM pathway that five hypothetical transposase and integrase genes are located next to the self-resistance gene *griR*.[1]

Validation of the *mypA* gene sequence

Three giant NRPS genes *mypABC* are proposed to build the decapeptide backbone of MPs. However, due to their highly repetitive sequences, the frequently experienced assembly dilemma with large modular NRPS genes occurred. Two independent PacBio sequencing attempts resulted in two versions of *mypA* with different lengths, 22,866 bp and 26,007 bp (with one additional NRPS module). Comprehensive PCR amplification and restriction digestion analysis of the repetitive region confirmed the shorter version, which fits the common colinearity rule for NRPS (Supplementary Figs. 1-3).

The domain analysis of these two *mypA* versions indicated the difference is one extra Leu-utilizing NRPS module 4* in the longer version and between module 4 and module 5 in the shorter version (Supplementary Fig. 2). In the long version, modules 4 and 4* are almost identical (Supplementary Fig. 1b). The high identity between the condensation (C) domains from modules 4, 4*, and 5 caused the situation to be more difficult (Supplementary Fig. 1b). However, the inconsistency between ten amino acid building blocks and eleven NRPS modules suggested a deviation from the collinearity rule in MPs biosynthesis. To verify whether the redundant module was an artifact that resulted from the assembly of highly repetitive DNA sequences, we thus took a closer look at this region and PCR amplified several fragments within and beyond the repetitive region (Supplementary Fig. 2). The sequences of these fragments were eventually determined by a series of walking sequencing and restriction digest analyses, which excludes the presence of the additional module and confirms the shorter version to be the correct one without module skipping during MP biosynthesis (Supplementary Figs. 2 and 3).

The α-ketobutyric acid moiety formation in MP biosynthesis

Frequently, the acyl substrates are adenylated by a fatty acyl-AMP ligase (FAAL) and the activated acyl-adenylates will be installed onto the concomitant acyl carrier protein (ACP) for further biosynthesis.[2] However, the FAAL gene has not been observed in the MP BGC. Nevertheless, the acyl-CoA could also be directly utilized to acylate the following amino acid by the starter C domain without the aid of FAAL and ACP.[3] However, the direct use of acyl-CoA by the starter C domain in MypA is unlikely since the initial building block in MPs is always the α-ketobutyric moiety, which is in contrast to the broad substrate spectrum of such particular C domains.[3, 4] It is worth noting that other uncommon strategies exist in the NRPS-mediated lipopeptide chain initiation. For instance, fatty acid synthase (FAS)-like enzymes are involved in the formation of the C6 fatty acid side chain of calcium-dependent antibiotics (CDAs).[5] Type II FAS genes are also present in the MPs BGC, *mypJ* has been found to encode a KAS III homologous protein, while the deduced product of the accompanying gene *mypI* is a discrete ACP. The active site analysis of KAS III proteins indicated that MypJ shows low similarity to DpsC and CerJ, which exhibited acyl activation and ester formation activity respectively, but more similar to ZhuH in R1128 biosynthesis (Supplementary Table 4).[6-8] Therefore, it is hypothesized that the holo-MypI is either malonylated by the malonyl-CoA-acyl carrier protein transacylase (MCAT) FabD from fatty acid biosynthesis or functions as a self-priming ACP that widely exists in fatty acid and type II PKS biosynthesis.[9, 10] Subsequently, MypJ extends the acyl chain by a molecule of acetyl-CoA to form the acetoacetyl-*S*-ACP species. The acetoacetyl-*S*-MypI is probably initially reduced to butyryl-*S*-MypI by reductases from the type II fatty acid biosynthesis. Whether the butyryl-*S*-MypI was oxidized before or after it is transferred onto the NRPS assembly line by C1 is yet unclear. Moreover, the origin of the α-keto group remained inexplicable. Nevertheless, a P450 oxidase encoding gene *mypP* is assumed to be responsible for the cryptic α-keto group formation since no other obvious oxidation steps are needed in MP biosynthesis.

To our knowledge, the N-terminal α-ketobutyric acid moiety of MPs is unique and so far not observed in other lipopeptides. The origin and activation of this four-carbon chain are still elusive. The most common concomitant FAAL and ACP genes for NRPS lipoinitiation were not observed here.[2] Also, the alternative direct use of acyl-CoA by the starter C domain in MypA is unlikely since the constant α-ketobutyric moiety in MPs is in contrast to the broad substrate spectrum of such special C domains.[3, 4]

Marfey's analysis

As shown in Supplementary Figs. 25, 38, 52, and 64, the reference for 5-methyl-norleucine was only available as an isomer and therefore just used to distinguish 5-methyl-norleucine from N-methyl-leucine. The 4-MePro comprised in MP B was found to be L-configured by Marfeys' analysis (Supplementary Fig. 36). For the 4-PrPro in MP D and homonorleucine residue in MP E, L-configuration has been proposed due to the lack of epimerase domains in respective NRPS modules in MP BGC albeit no reference was available for experimental proof of the stereochemistry.

Crystal structure of DnaN in complex with MP A

The crystal structures of msDnaN in complex with MP A, GM, and CGM, respectively, were refined to R/R_{free} values of 22/26 %, 17/19 % and 17/18 %, respectively (Supplementary Table 7). The MP A complex crystallized in space group C2 with one dimeric ring in the asymmetric unit, while the GM / CGM complexes crystallized in space group C222 with one protein chain in the asymmetric unit and the dimer formed by crystal symmetry. All ligands were clearly defined in the electron density map (Supplementary Fig. 6). The tilting of MP A away from the protein in the region around Pro8 can be attributed to the longer 5-methyl-l-norleucine residue of MP A at position 6. This interpretation is also supported by the crystallographic B-factors of the ligands being generally the highest for Pro8 and low for the linear part and the adjacent residues (Supplementary Fig. 10).

Supplementary Figures

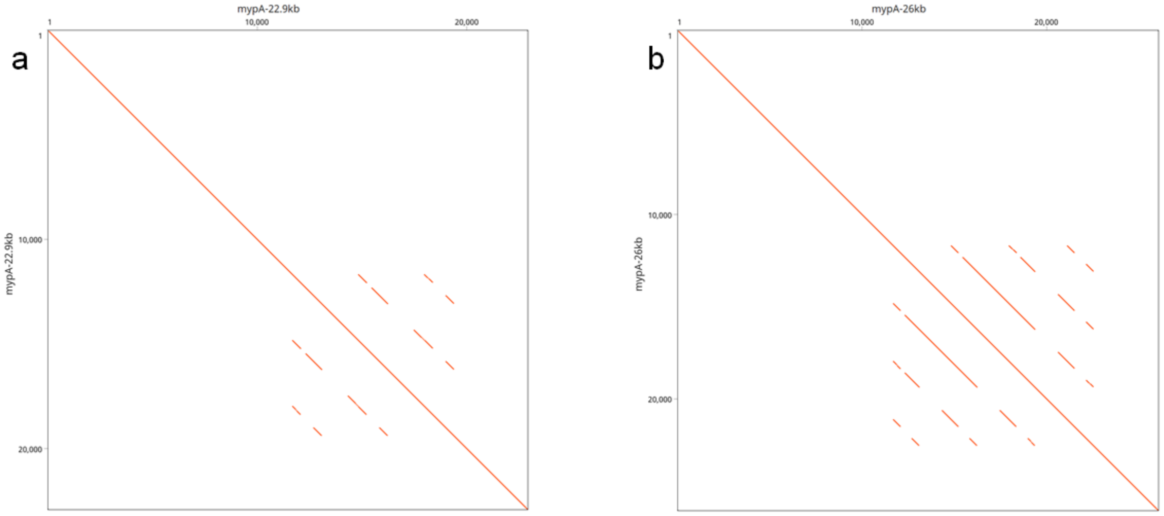

Supplementary Figure 1. Dot plots of two versions f mypA genes. The repetitive regions are shown by dot plots of *mypA*-22.9kb (a) and *mypA*-26kb (b) gene sequences against themselves, respectively. Red diagonal lines are the regions of similarity between two sequences. In this self-comparison context, they would be repetitive regions in the gene.

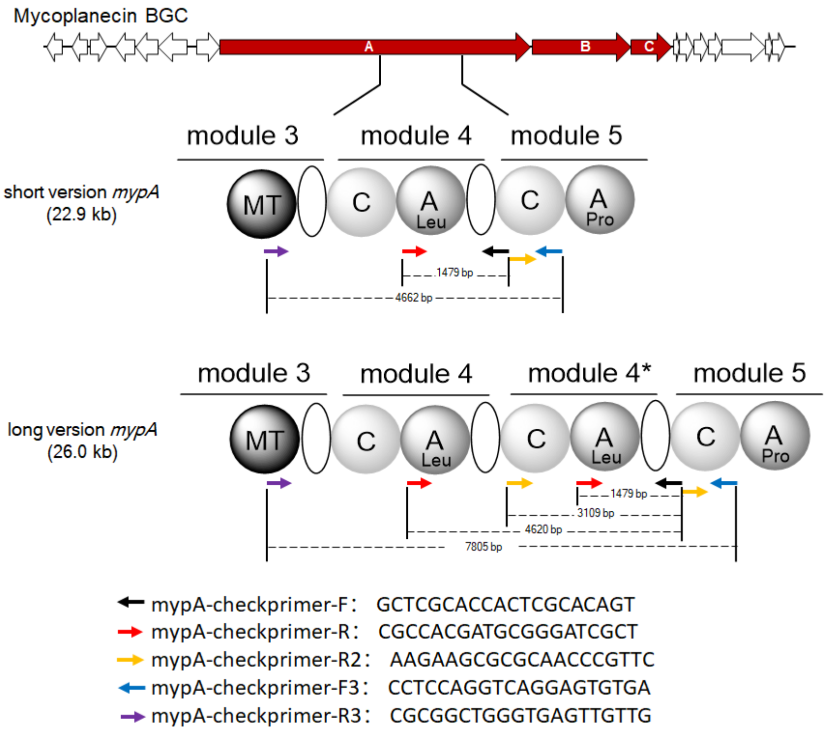

Supplementary Figure 2. Design of a combination of PCR amplification to verify the mypA gene sequence. Three sets of primers were designed verify the suspicious repetitive DNA region of *mypA*. The PCR amplification with the primer pair mypA-checkprimer-F/R should only give one 1479 bp fragment if *mypA* is the short version but two fragments should appear with the additional 4620 bp one if *mypA* is the long version. With the second pair of primers mypA-checkprimer-F/R2, the PCR will give no band with the short version *mypA* while a 3109 bp band should appear with the long version *mypA*. The third primer set mypA-checkprimer-F3/R3 beyond the questionable region should give more confirmative information as different sizes of amplicons (4662 bp for short *mypA* and 7805 bp for long *mypA*) will appear. The primer sequences of mypA-checkprimer-R (red) and mypA-checkprimer-R2 (orange) occur twice in the 26kb version of mypA.

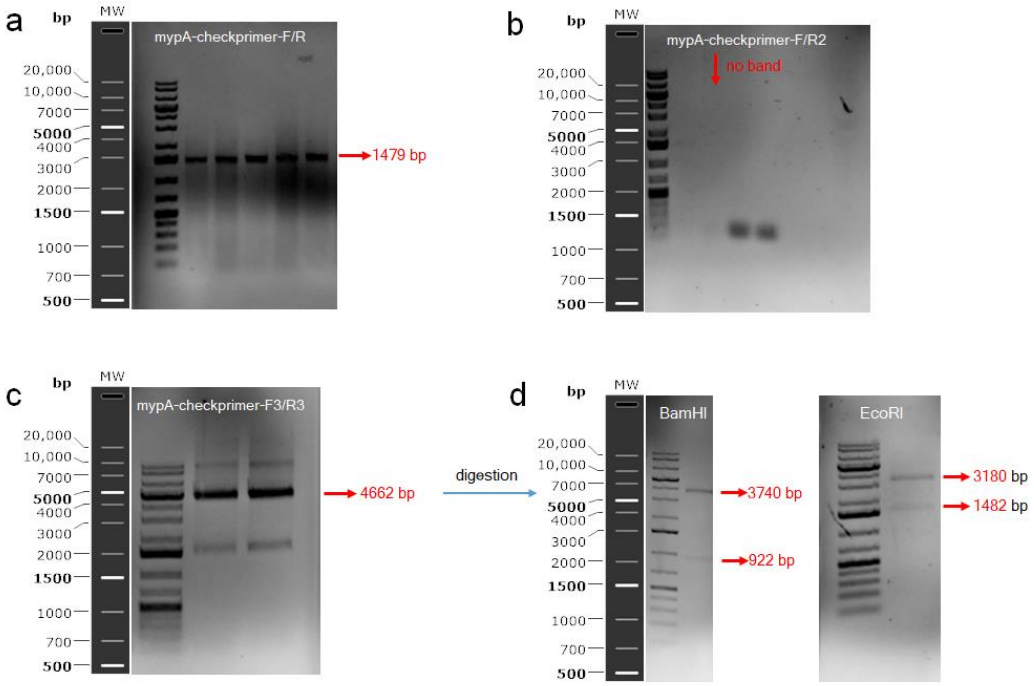

Supplementary Figure 3. PCR amplified fragments from the genomic DNA of *A. awajinensis* subsp. *mycoplanecinus* subsp. nov. ATCC 33919 using a combination of primers confirm the mypA gene should be the 22.9 kb version. a) Amplification using mypA-checkprimer-F and R only delivered one 1479 bp fragment which agrees with the 22.9 kb version but not two fragments including the 4620 bp one present in the 26 kb version. b) Amplification using mypA-checkprimer-F and R2 delivered no band which agrees with the 22.9 kb version but not the 3109 bp band in the *mypA*-26 kb version. c) Amplification using mypA-checkprimer-F3 and R3 delivered the 4662 bp band which agrees with the 22.9 kb version but not the 7805 bp band in the *mypA*-26 kb version. d) Restriction enzyme digestion using *Bam*HI and *Eco*RI showed correct bands, respectively.

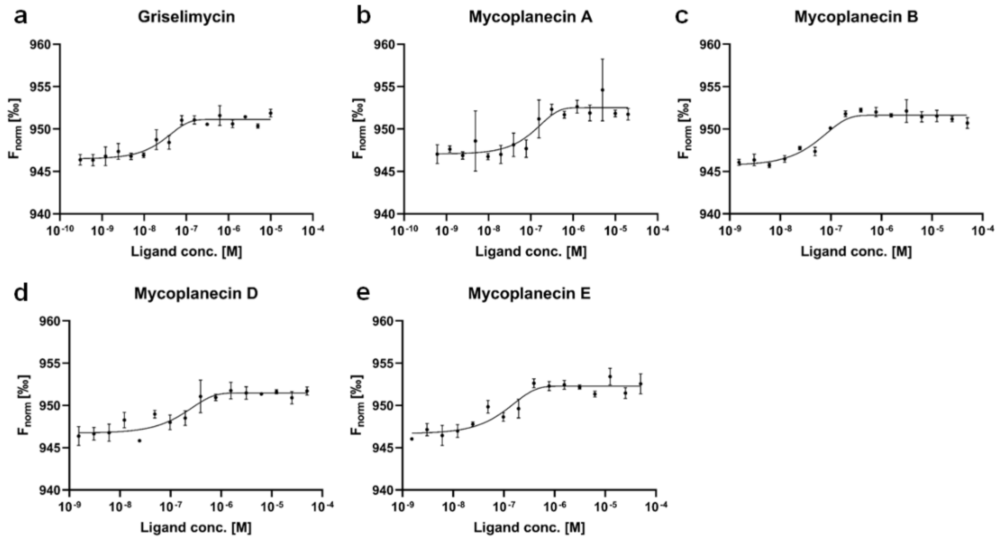

Supplementary Figure 4. MST dose-response curves for the binding interaction between BLUE-NHS labeled MsDnaN and different ligands. Experiment using 50 nM labeled DnaN and Griselimycin was titrated from 10,000 to 0.3 nM, K_D-value was determined to be 6.5±5.9 nM, MP A titrated from 20,000 to 0.6 nM, K_D-value 95.4±58.0 nM, MP B titrated from 50,000 to 1.5 nM, K_D-value 24.4±11.9 nM, MP D titrated from 50,000 to 1.5 nM, K_D-value 134.2±69.3 nM, MP E titrated from 50,000 to 1.5 nM, K_D-value 64.4 ±33.2 nM. The K_D-values are derived from three independent experiments (n=3) for each of the congeners.

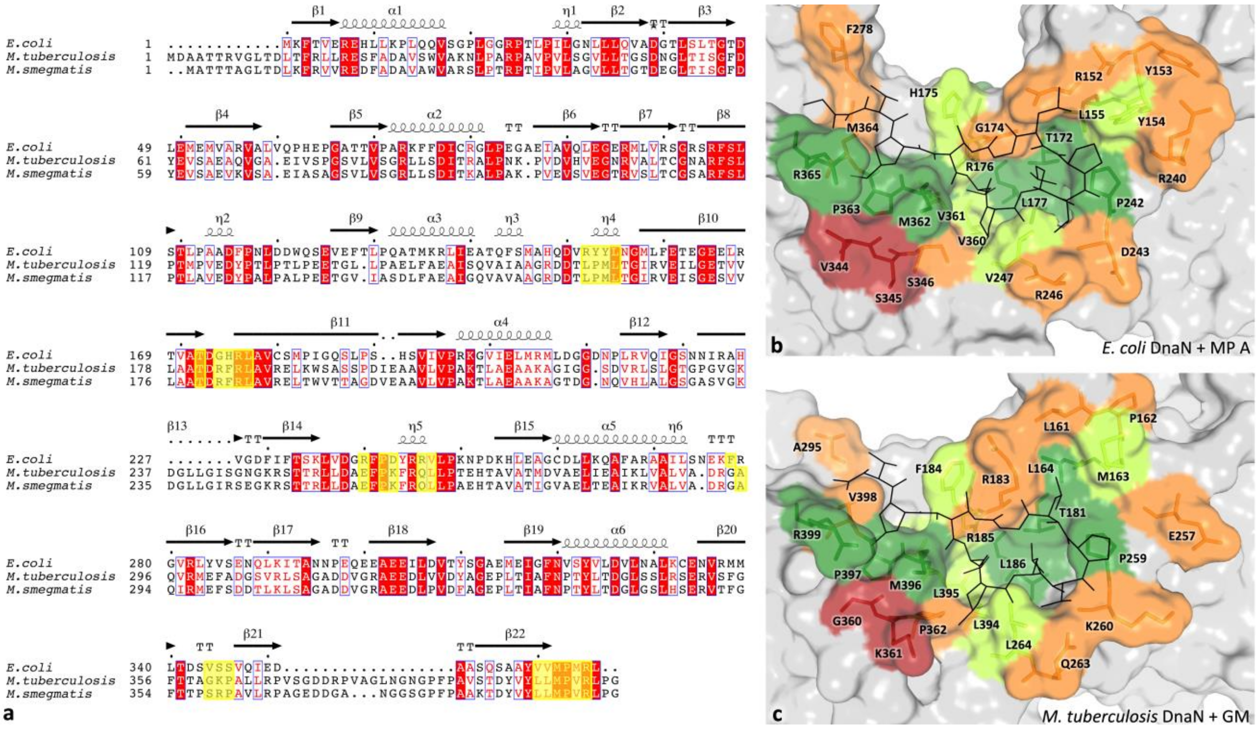

Supplementary Figure 5. **Comparison of *E. coli* and mycobacterial DnaNs. a Sequence alignment (Clustal Omega) of DnaN from *E. coli*, *M. tuberculosis* and *M. smegmatis*, formatted with ESPRIPT.[11, 12] The secondary structure annotation has been taken from ecDnaN in complex with MP A. Both mycobacterial proteins share 29 % sequence identity with ecDnaN and 76 % with eachother. The ligand binding site (highlighted in yellow) has been defined after superimposition of all analyzed ecDnaN or mycobacterial DnaN structures (single-chain level) as all residues in positions where at least in one of the structures the distance between a ligand atom and an atom of a residue is not further than 4 Å. b Surface representation of *E. coli* DnaN in complex with MP A. The residues of the binding site are indicated by labels and colored according to their conservation. Green: Identical in all analyzed proteins. Lime: Conservative substitution. Orange: Major difference between *E. coli* and mycobacterial DnaN. Red: Differences between all structures. c Same as b, but shown for *M. tuberculosis* DnaN in complex with GM.**

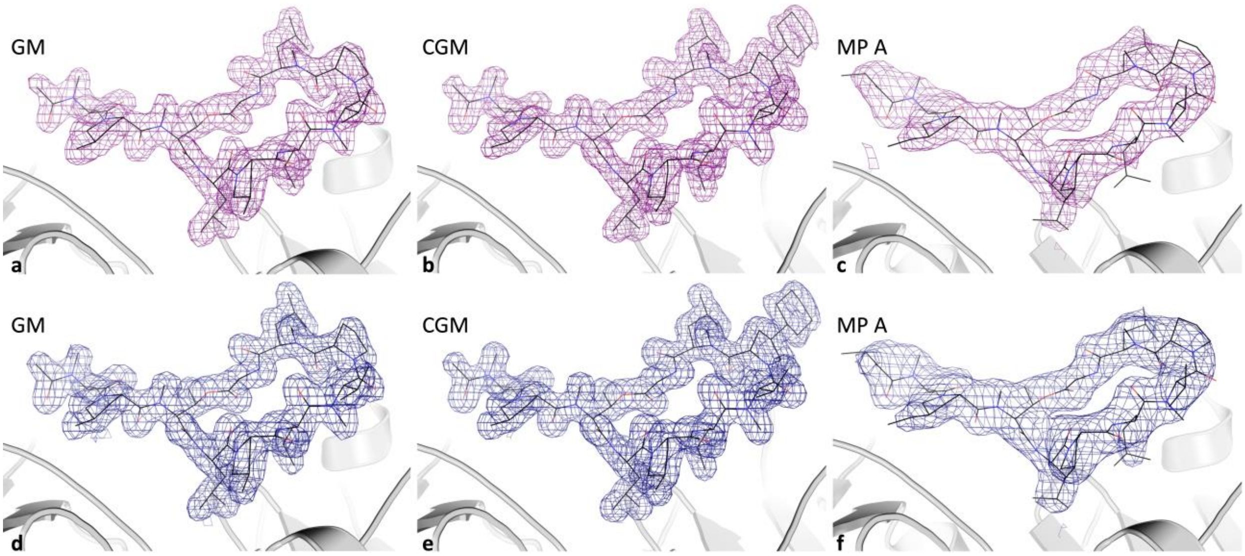

Supplementary Figure 6. Electron density maps of the ligands in the ecDnaN co-structures. a – c Omit maps (Phenix composite omit map tool) for GM, CGM and MP A (purple meshes). **d - f** Refined $2mF_{obs}$-DF_{calc} electron density maps for GM, CGM and MP A are shown as (blue meshes). All electron density maps are shown at a contour level of $\sigma = +1$.

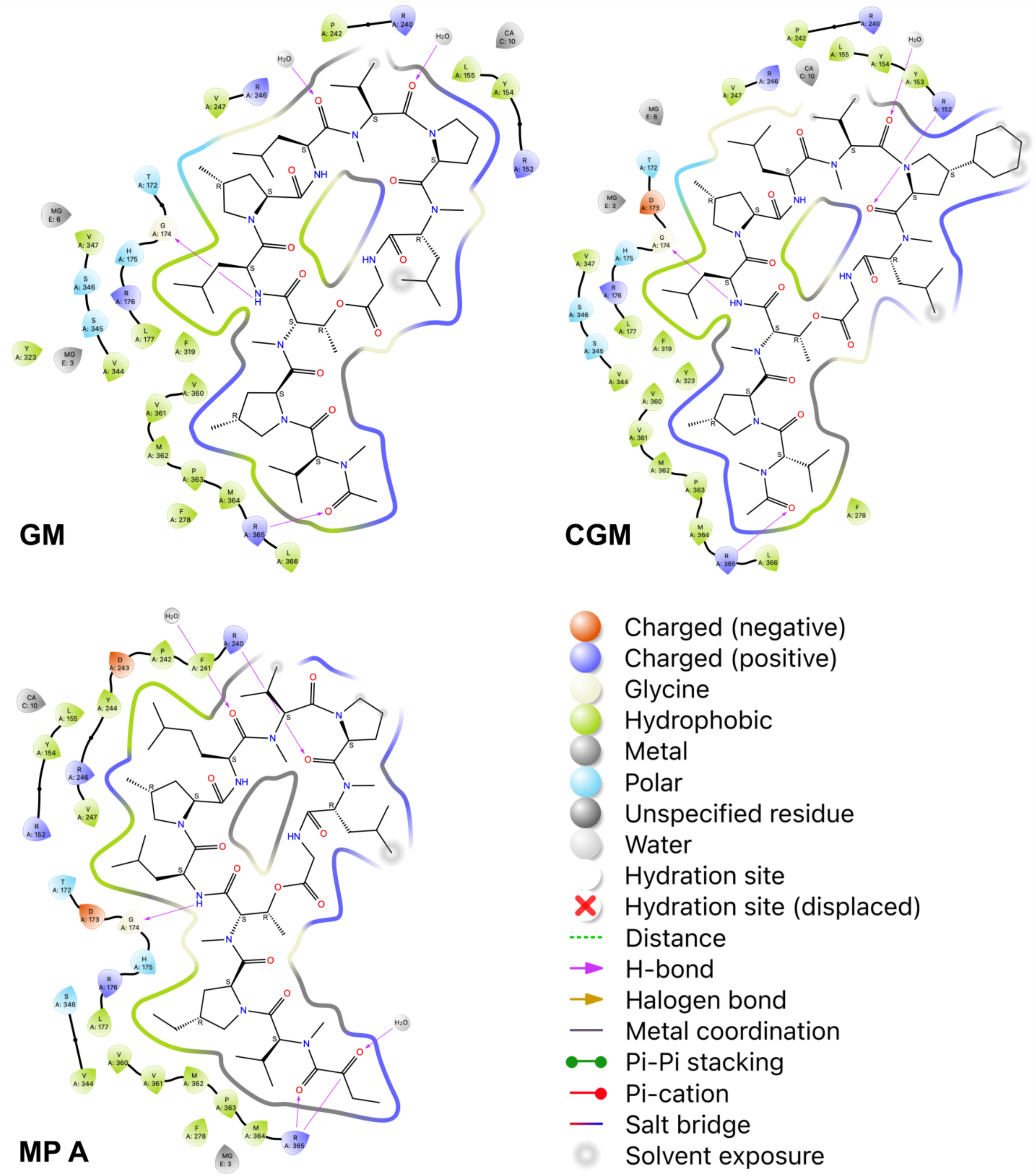

Supplementary Figure 7. Ligand interaction diagrams. Ligand interaction diagrams for GM, CGM and MP A generated from the corresponding ecDnaN co-crystal structures by Maestro from the Schrödinger2020 suite (Schrödinger, LLC).

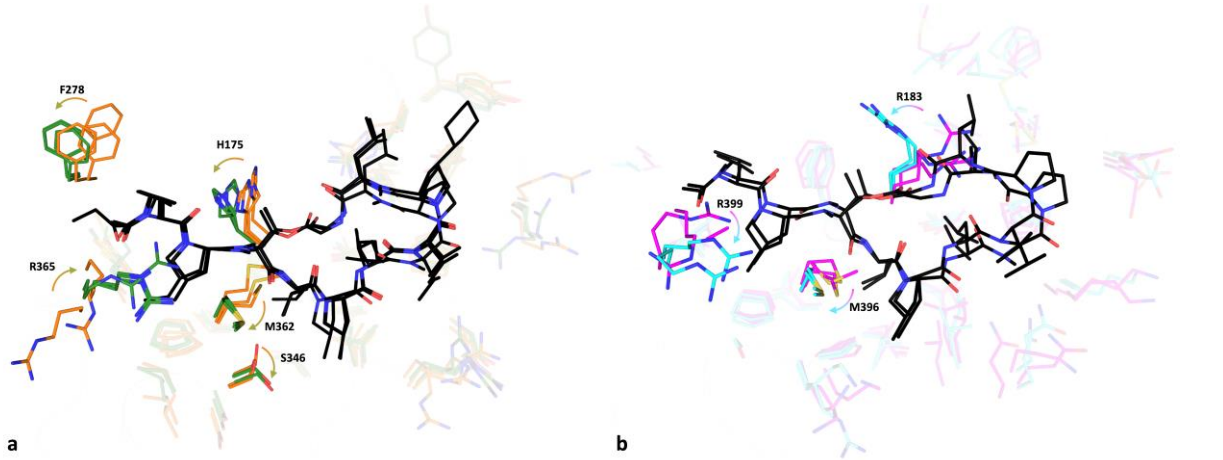

Supplementary Figure 8. Conformational changes upon ligand binding. a Superimposition of the ecDnaN co-crystal structures with structures of uncomplexed ecDnaN available from the PDB (1MMI, 4K3S, 4PNV).[13-15] Only the compounds (black) and the side chains of the binding-site (ligand-free: orange, complexed: green) are shown. Residues undergoing significant conformational changes upon ligand-binding are highlighted and side chain movements are indicated by arrows. b Superimposition of the previously published M. tuberculosis (5AGU) and M. smegmatis (5AH2) DnaN/GM complexes with structures of uncomplexed M. tuberculosis DnaN available from the PDB (3P16, 3RB9, 4TR7).[16-19] Only the compounds (black) and the side chains of the binding-site (ligand-free: magenta, complexed: cyan) are shown. Residues undergoing significant conformational changes upon ligand-binding are highlighted and side chain movements are indicated by arrows. Structures were superimposed on single-chain level (monomeric DnaN).

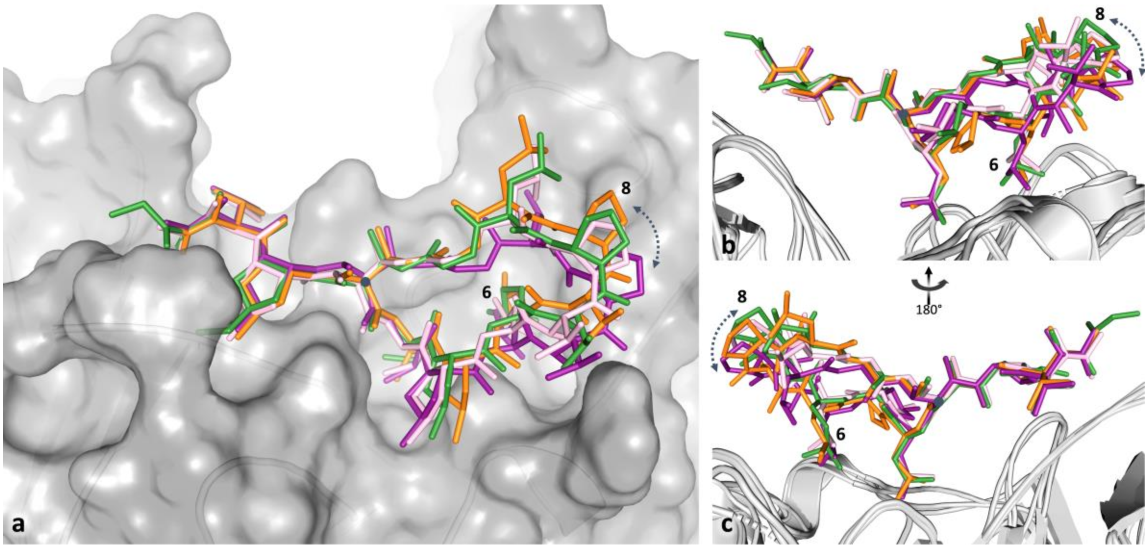

Supplementary Figure 9. Binding pose comparison of MP A and GM in the ecDnaN complexes with GM bound to mycobacterial DnaNs. a Superimposition of the ecDnaN co-crystal structures containing MPA (green) and GM (orange), with the complexes of GM and *M. tuberculosis* DnaN (purple, PDB: 5AGU) and *M. smegmatis* DnaN (pink, PDB: 5AH2). The protein surface of ecDnaN is shown in grey. A dashed double arrow indicates a tilt of the macrocycle in the comparison of 7-10°. (Pivot point shown as grey dot.) **b** Side view of the superposition from A with the protein shown as cartoon. **c** Same as panel b, but turned 180° around the Y axis. Structures were superimposed on single-chain level (monomeric DnaN).

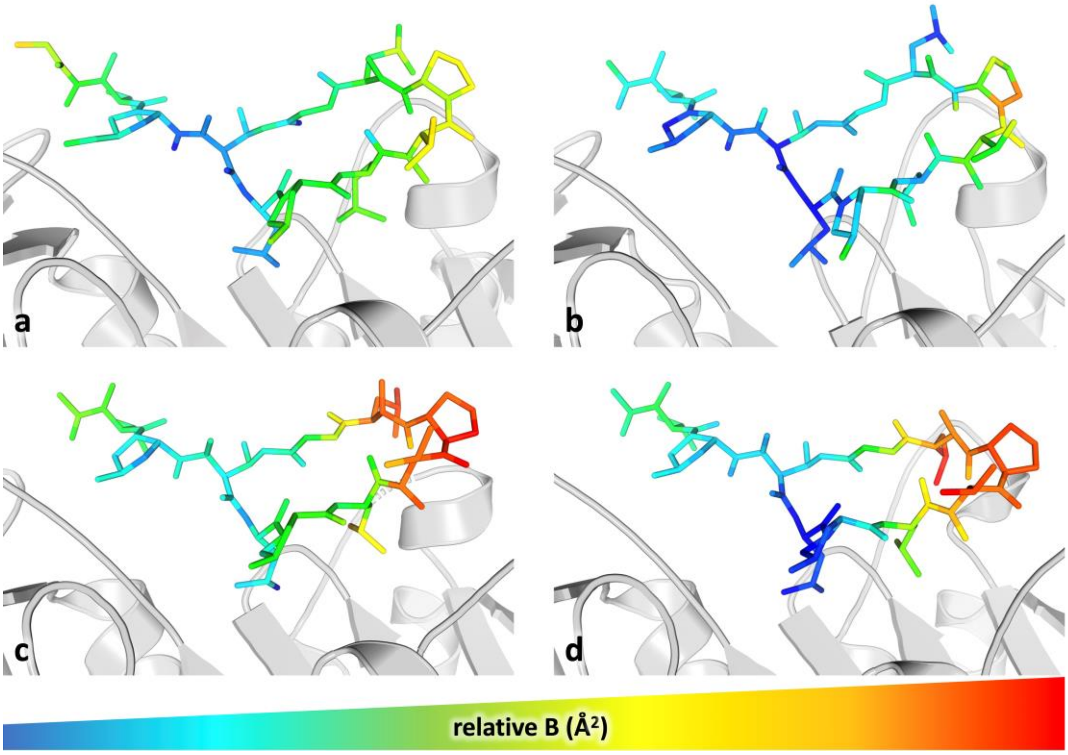

Supplementary Figure 10. Relative crystallographic B-factors of MP A and GM in the complexes ecDnaN and the mycobacterial proteins. Ligand atoms are coloured according to their crystallographic B-factors from low (blue) to high(red). a MP A bound to ecDnaN (PDB: 8CIZ). b GM bound to ecDnaN (PDB: 8CIX). c GM bound to *M. smegmatis* DnaN (PDB: 5AH2). d GM bound to *M. tuberculosis* DnaN (PDB: 5AGU).

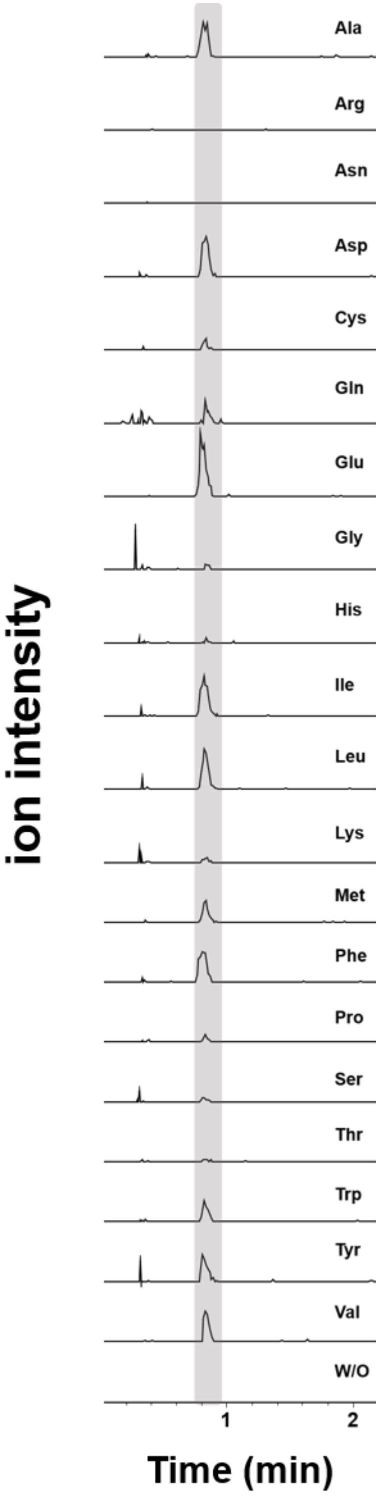

Supplementary Figure 11. *In vitro* transamination of purified MypL$_C$. Transamination was performed in the presence of purified MypL$_C$, PLP, 4-methyl-2-oxohexanoic acid, proteinogenic amino acids as amino donors respectively *in vitro*, and was detected by LC-MS. The expected homoisoleucine product mass peak was indicated in grey.

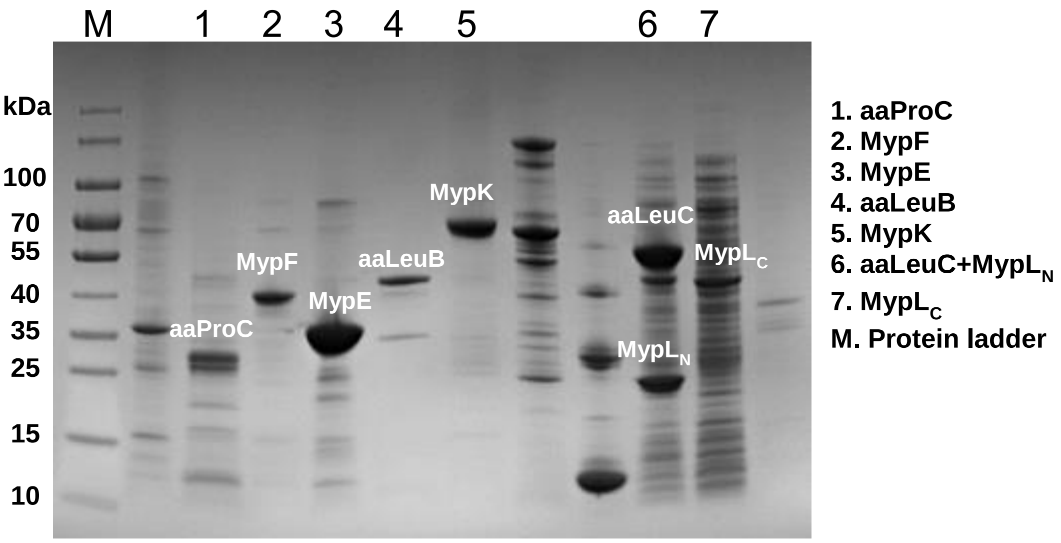

Supplementary Figure 12. SDS-PAGE gel of recombinant proteins involved in this study.

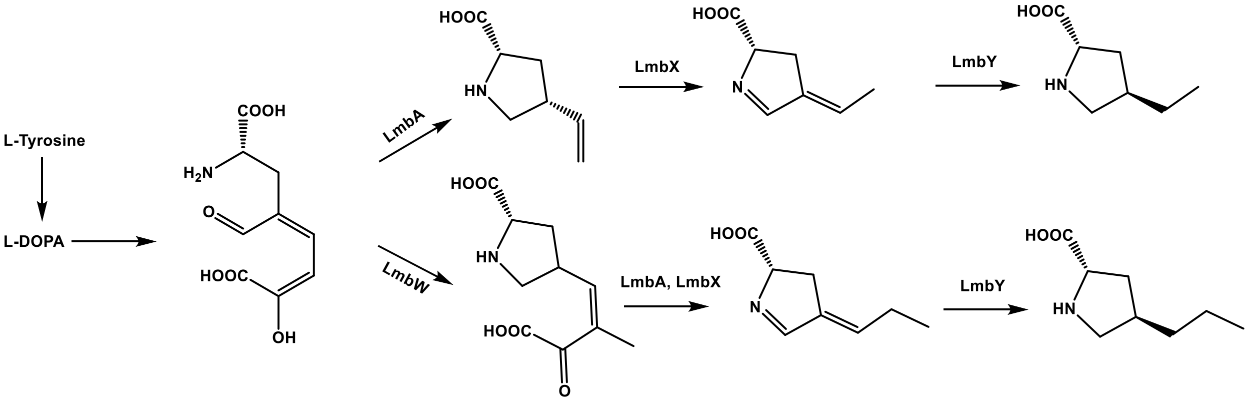

Supplementary Figure 13. Biosynthetic routes to 4-EtPro and 4-PrPro in lincomycins biosynthesis.[20]

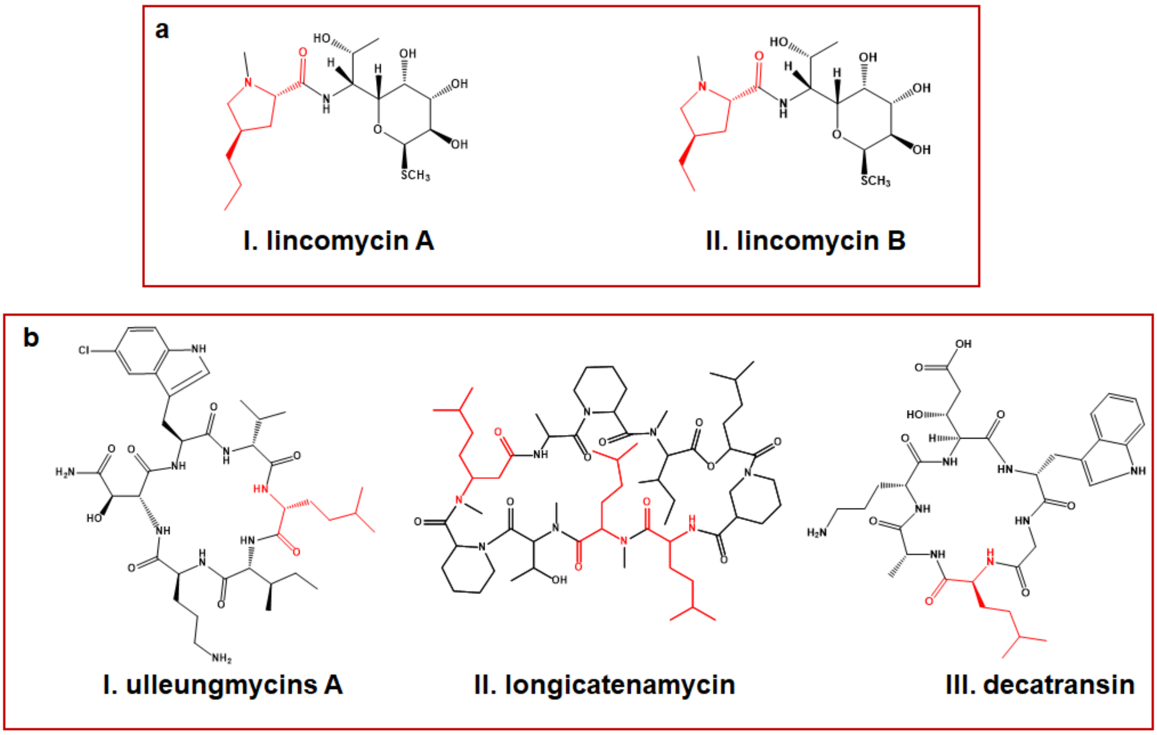

Supplementary Figure 14. Examples of natural products containing 4-ethylproline, 4-propylproline and homoleucine. a Compounds incorporating the 4-alkyl-L-proline. I: lincomycin A comprised trans-4-propyl-L-proline, II: lincomycin B comprised trans-4-ethyl-L-proline. b Compounds incorporating the homoleucine. I, II, III ulleungmycins A, longicatenamycin and decatransin was incorporated with homoleucine respectively.[21-24]

Structure elucidation of mycoplanecins using NMR and Marfey's analysis

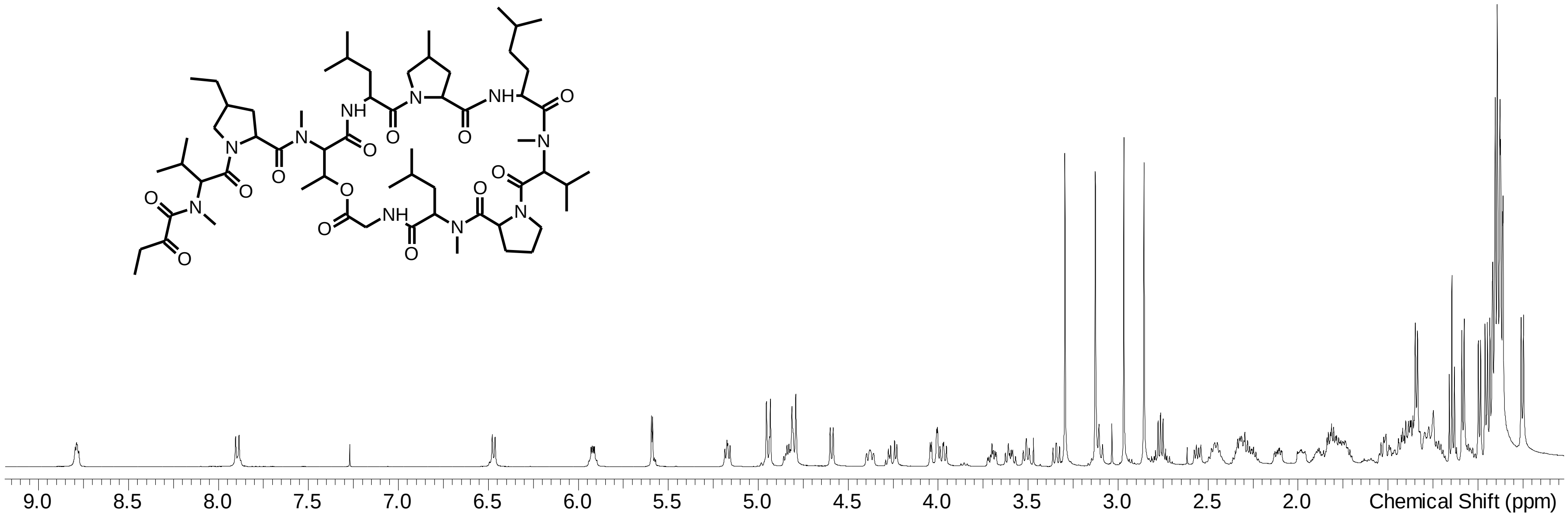

Supplementary Figure 15. 1H-spectrum of MP A in CDCl3 at 500 MHz.

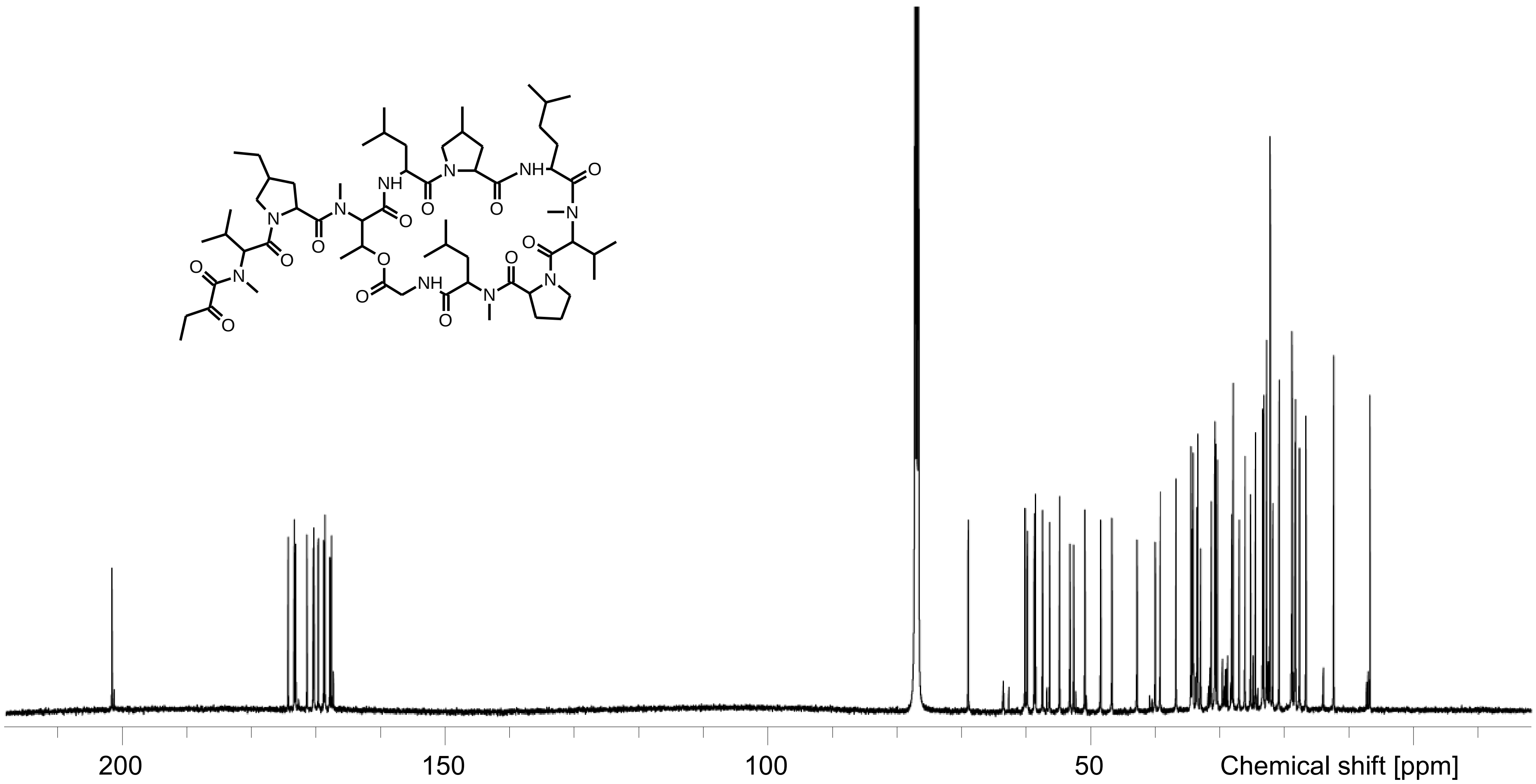

Supplementary Figure 16. ^{13}C-spectrum of MP A in $CDCl_3$ at 125 MHz.

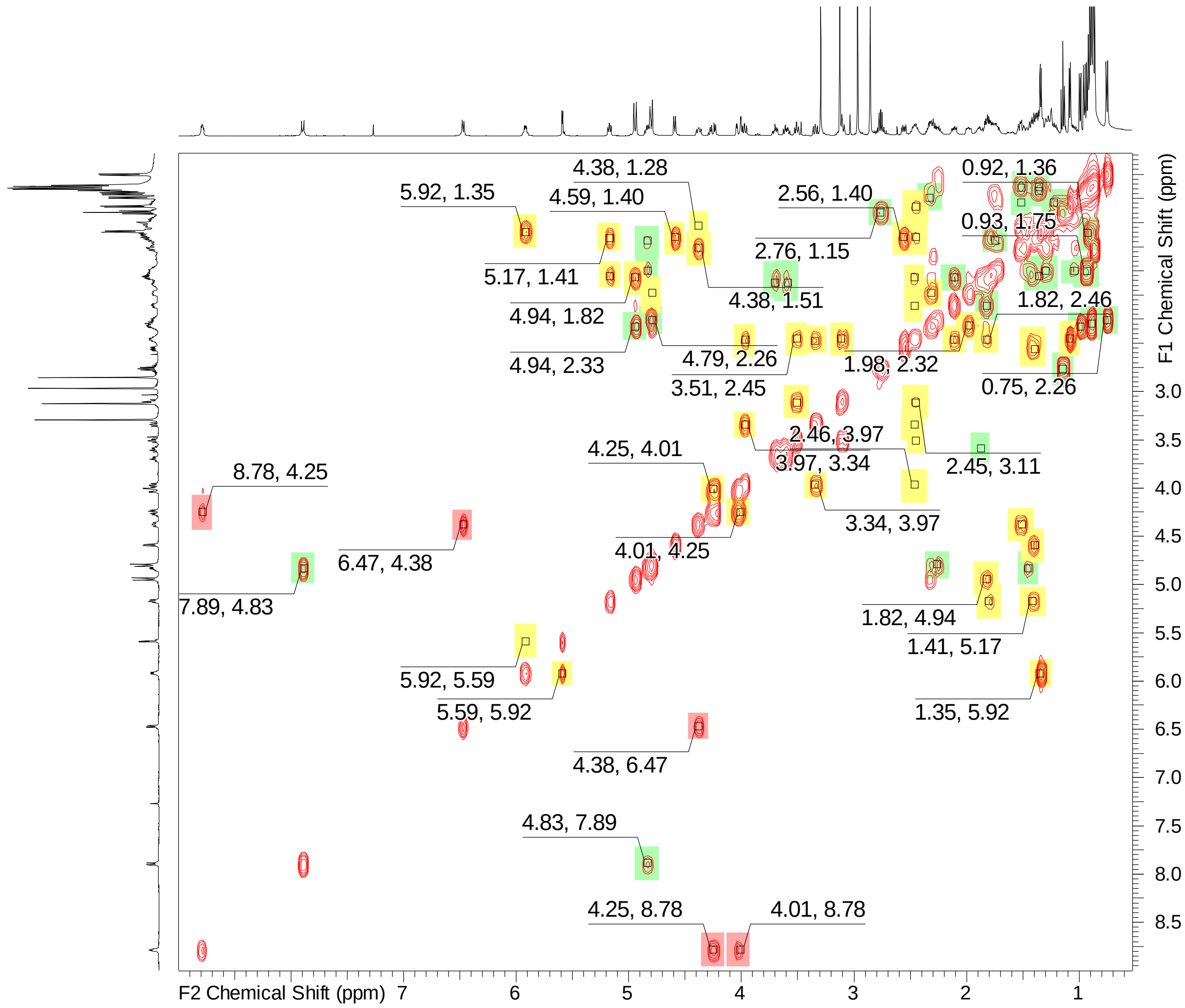

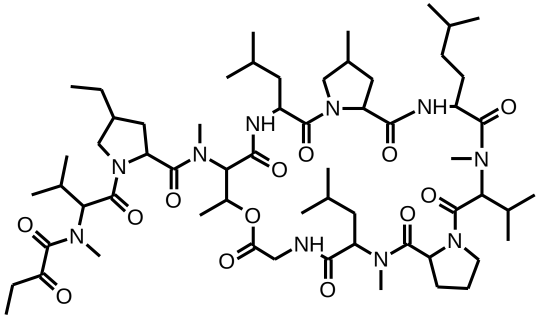

Supplementary Figure 17. COSY-spectrum of MP A in CDCl3 at 500 MHz.

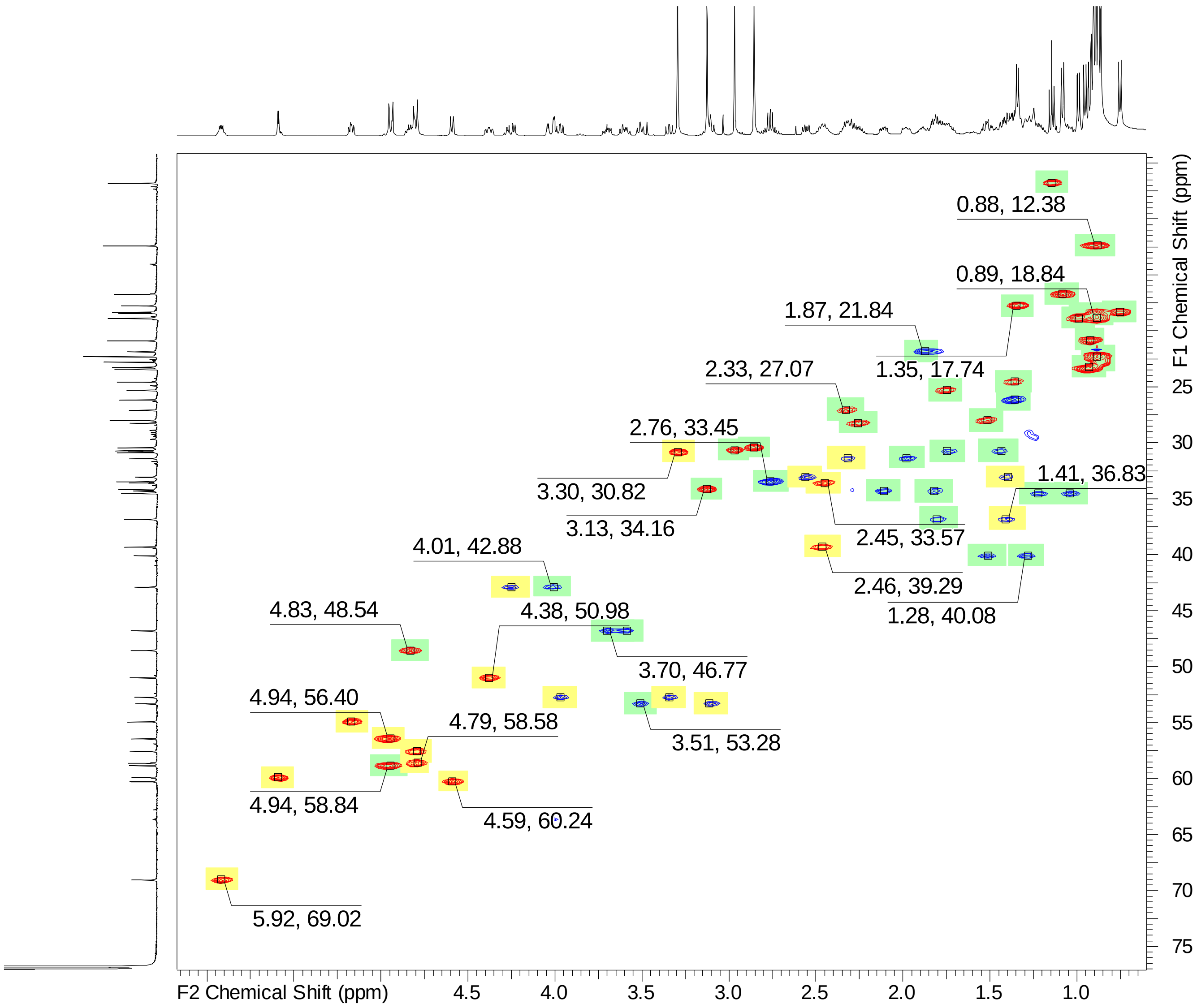

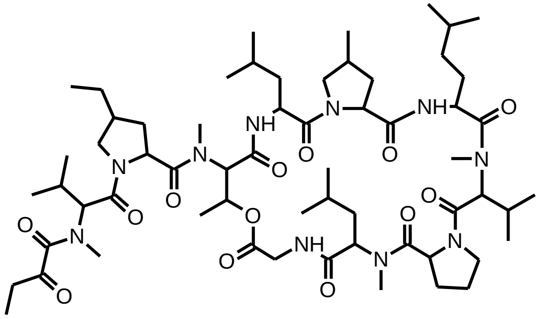

Supplementary Figure 18. HSQC-spectrum of MP A in $CDCl_3$ at 500 MHz (^1H)/125 MHz (^{13}C).

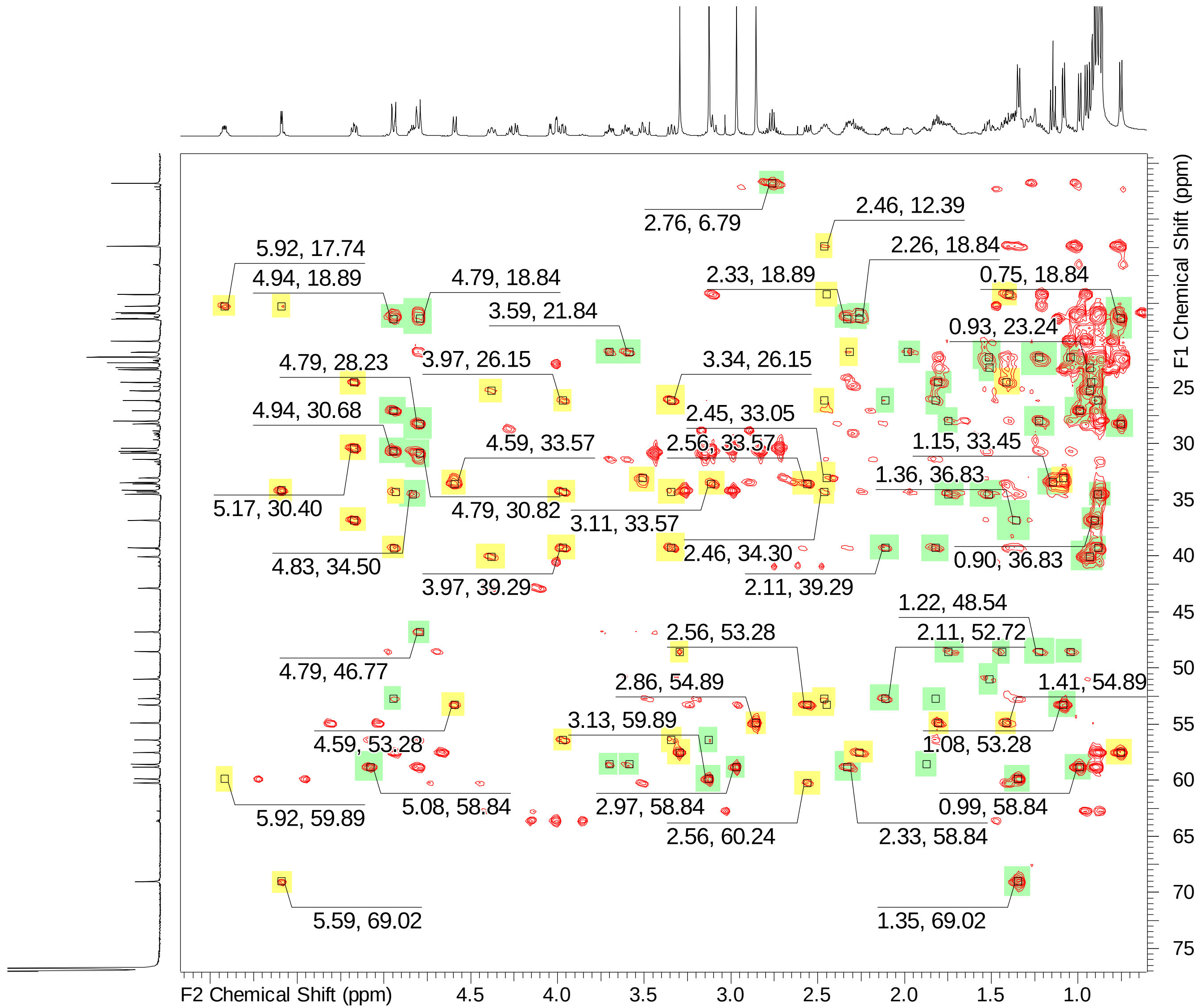

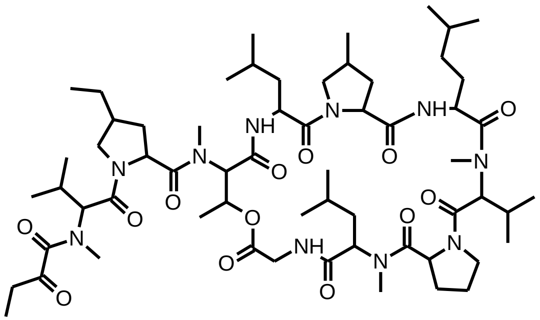

Supplementary Figure 19. HMBC-spectrum of MP A in CDCl3 at 500 MHz (1H)/125 MHz (13C).

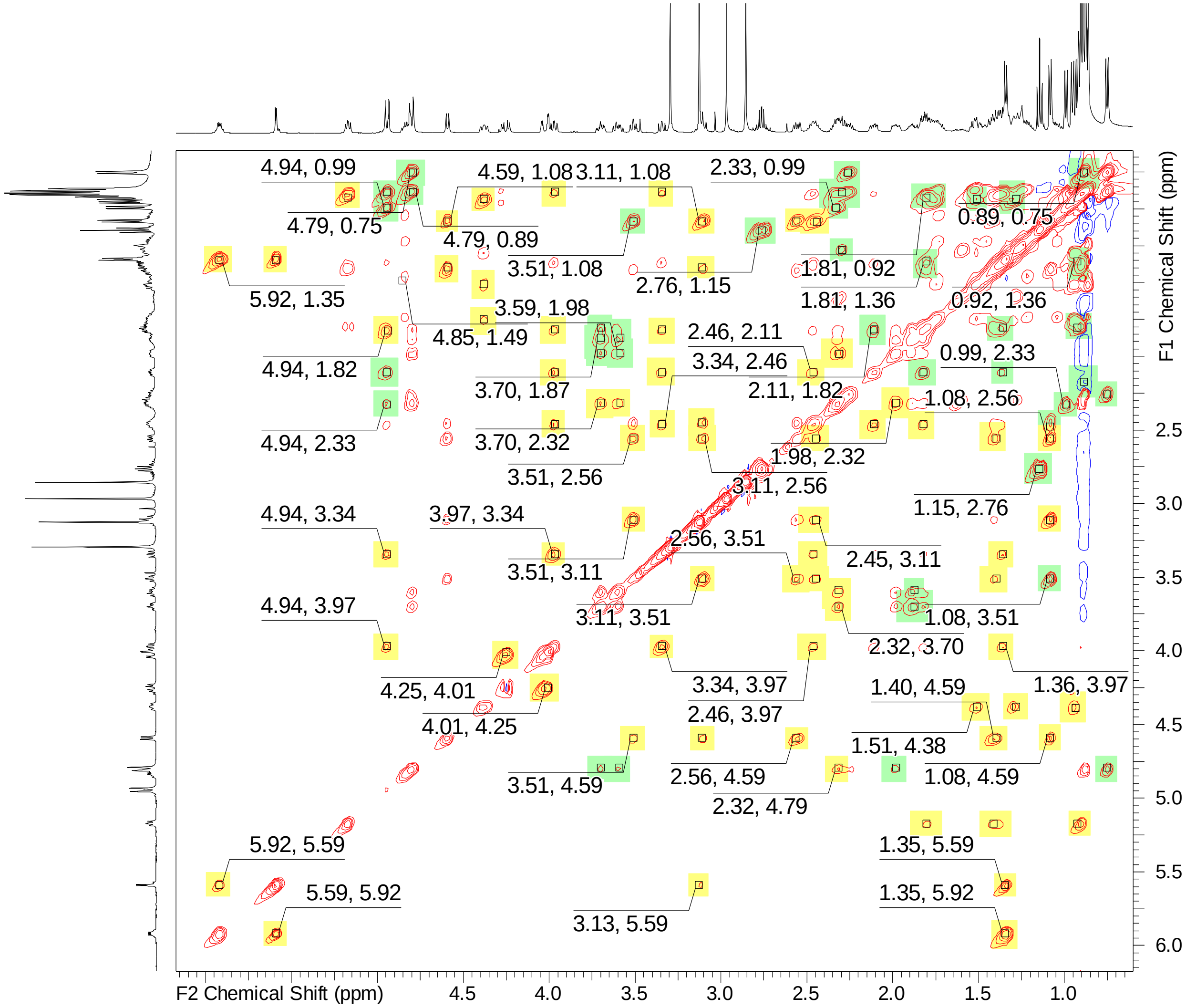

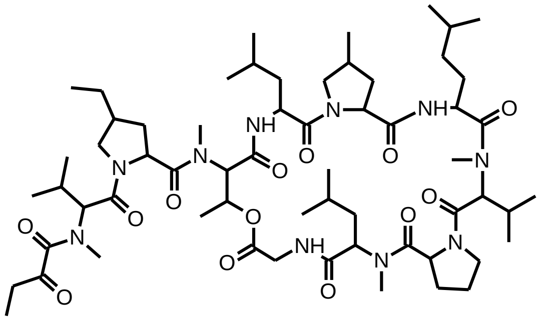

Supplementary Figure 20. TOCSY-spectrum of MP A in CDCl3 at 500 MHz.

Marfey's derivatization:

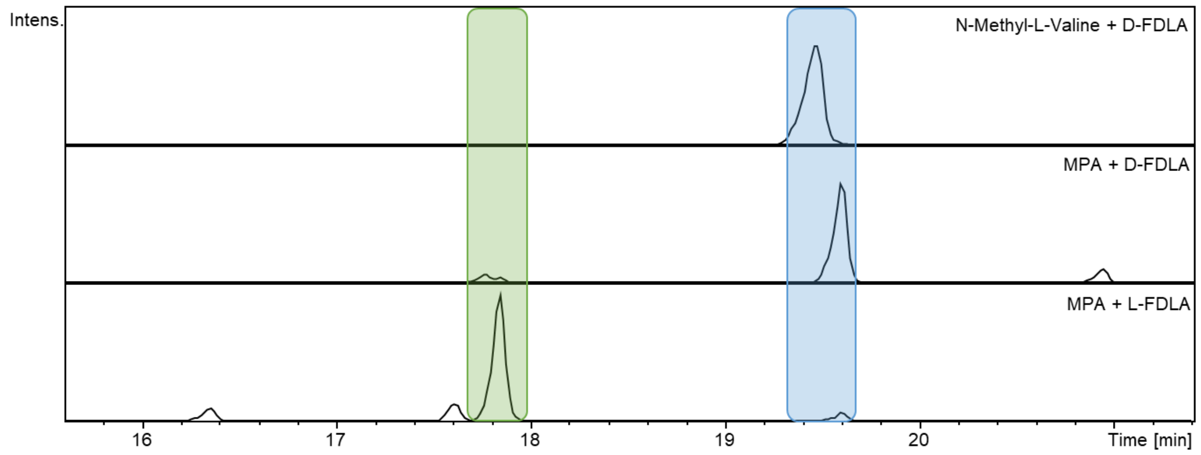

Supplementary Figure 21. Marfey's derivatization of reference N-Methyl-L-Valine (upper chromatogram) with D-FDLA and MP A with both D-FDLA (middle chromatogram) and L-FDLA (lower chromatogram). Retention time comparison represented as EICs; D-L-FDLA/L-D-FDLA highlighted in blue and L-L-FDLA/D-D-FDLA in green.

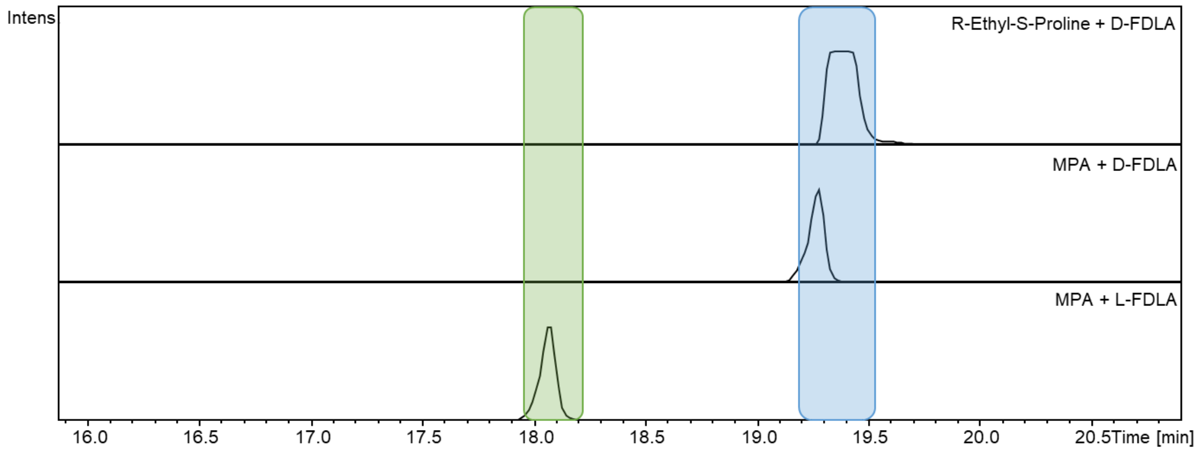

Supplementary Figure 22. Marfey's derivatization of reference R-Ethyl-S-Proline (upper chromatogram) with D-FDLA and MP A with both D-FDLA (middle chromatogram) and L-FDLA (lower chromatogram). Retention time comparison represented as EICs; D-L-FDLA/L-D-FDLA highlighted in blue and L-L-FDLA/D-D-FDLA in green.

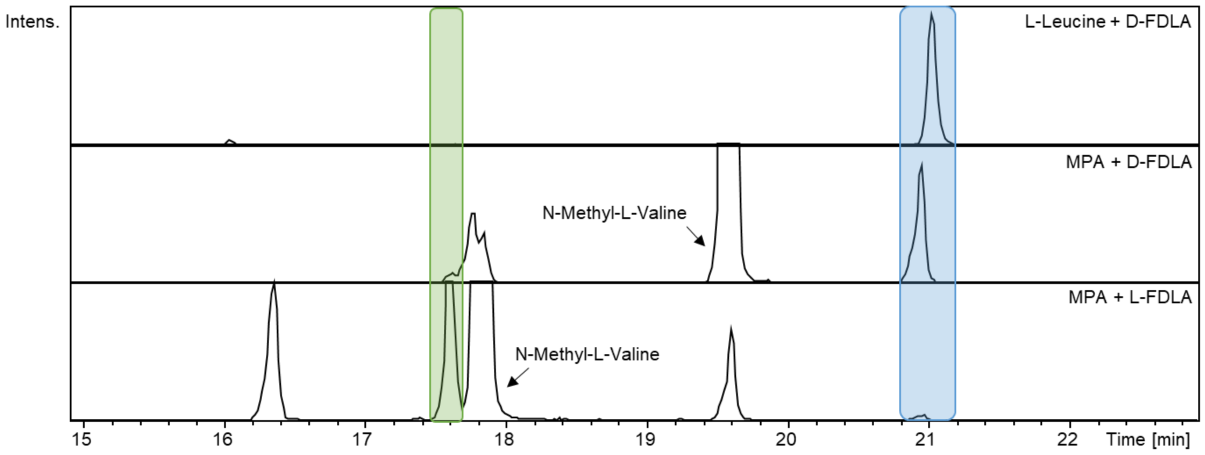

Supplementary Figure 23. Marfey's derivatization of reference L-Leucine (upper chromatogram) with D-FDLA and MP A with both D-FDLA (middle chromatogram) and L-FDLA (lower chromatogram). Retention time comparison represented as EICs; D-L-FDLA/L-D-FDLA highlighted in blue and L-L-FDLA/D-D-FDLA in green.

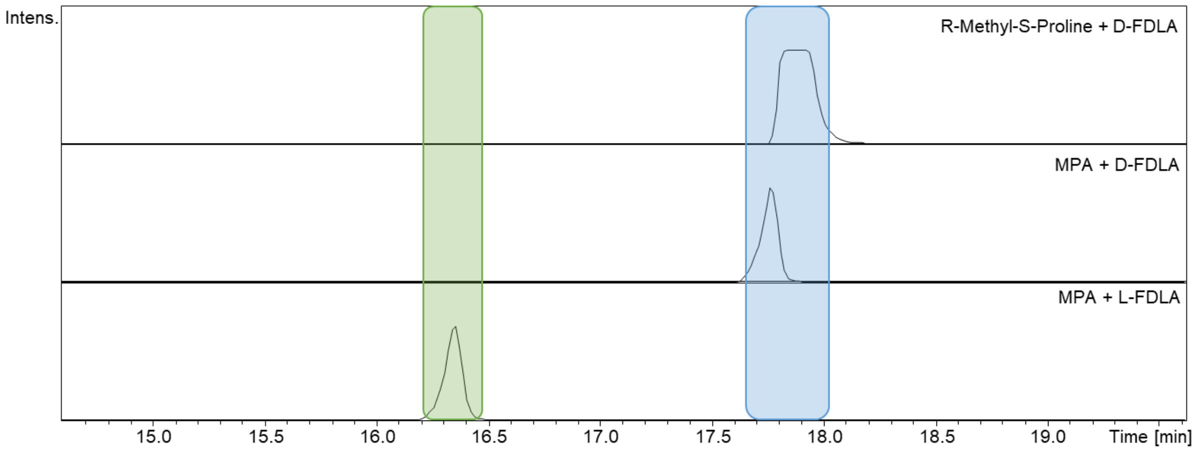

Supplementary Figure 24. Marfey's derivatization of reference R-Methyl-S-Proline (upper chromatogram) with D-FDLA and MP A with both D-FDLA (middle chromatogram) and L-FDLA (lower chromatogram). Retention time comparison represented as EICs; D-L-FDLA/L-D-FDLA highlighted in blue and L-L-FDLA/D-D-FDLA in green.

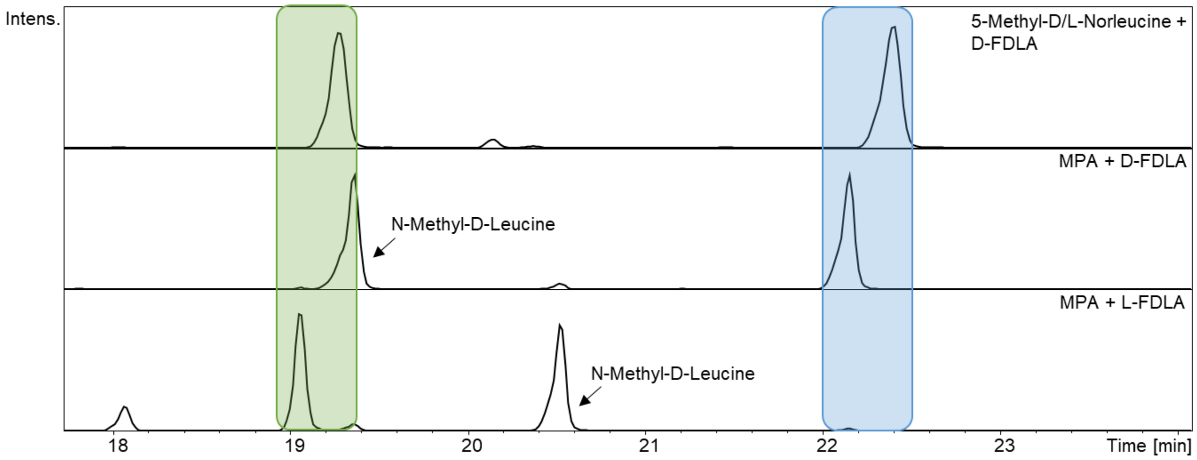

Supplementary Figure 25. Marfey's derivatization of reference 5-Methyl-D/L-Norleucine (upper chromatogram) with D-FDLA and MP A with both D-FDLA (middle chromatogram) and L-FDLA (lower chromatogram). Retention time comparison represented as EICs; D-L-FDLA/L-D-FDLA highlighted in blue and L-L-FDLA/D-D-FDLA in green.

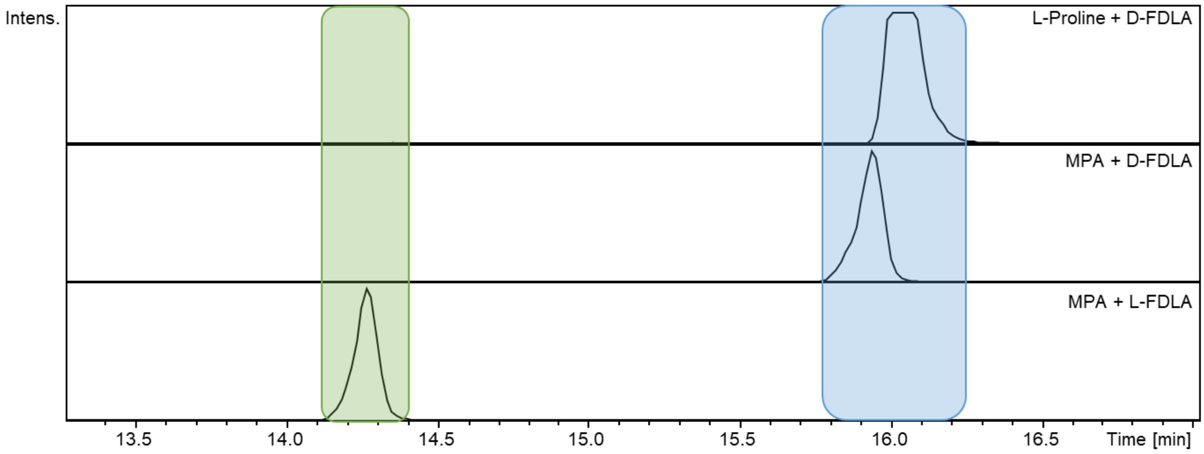

Supplementary Figure 26. Marfey's derivatization of reference L-Proline (upper chromatogram) with D-FDLA and MP A with both D-FDLA (middle chromatogram) and L-FDLA (lower chromatogram). Retention time comparison represented as EICs; D-L-FDLA/L-D-FDLA highlighted in blue and L-L-FDLA/D-D-FDLA in green.

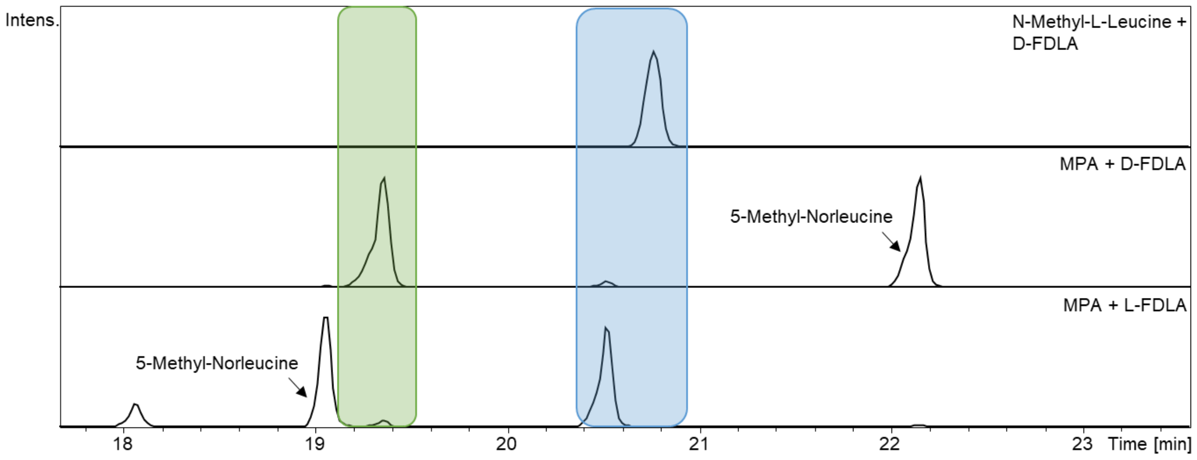

Supplementary Figure 27. Marfey's derivatization of reference N-Methyl-L-Leucine with D-FDLA (upper chromatogram) and MP A with both D-FDLA (middle chromatogram) and L-FDLA (lower chromatogram). Retention time comparison represented as EICs; D-L-FDLA/L-D-FDLA highlighted in blue and L-L-FDLA/D-D-FDLA in green.

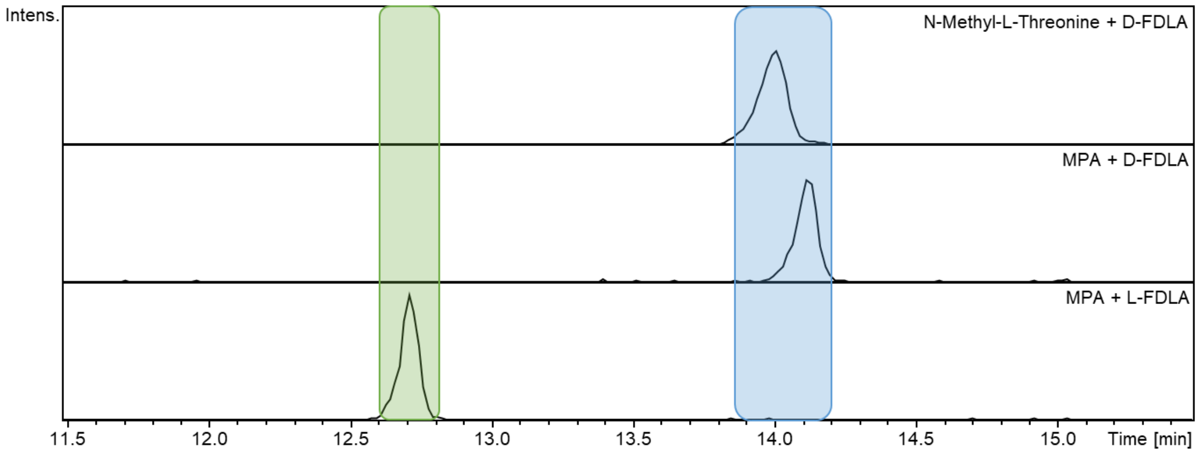

Supplementary Figure 28. Marfey's derivatization of reference N-Methyl-L-Threonine with D-FDLA (upper chromatogram) and MP A with both D-FDLA (middle chromatogram) and L-FDLA (lower chromatogram). Retention time comparison represented as EICs; D-L-FDLA/L-D-FDLA highlighted in blue and L-L-FDLA/D-D-FDLA in green.

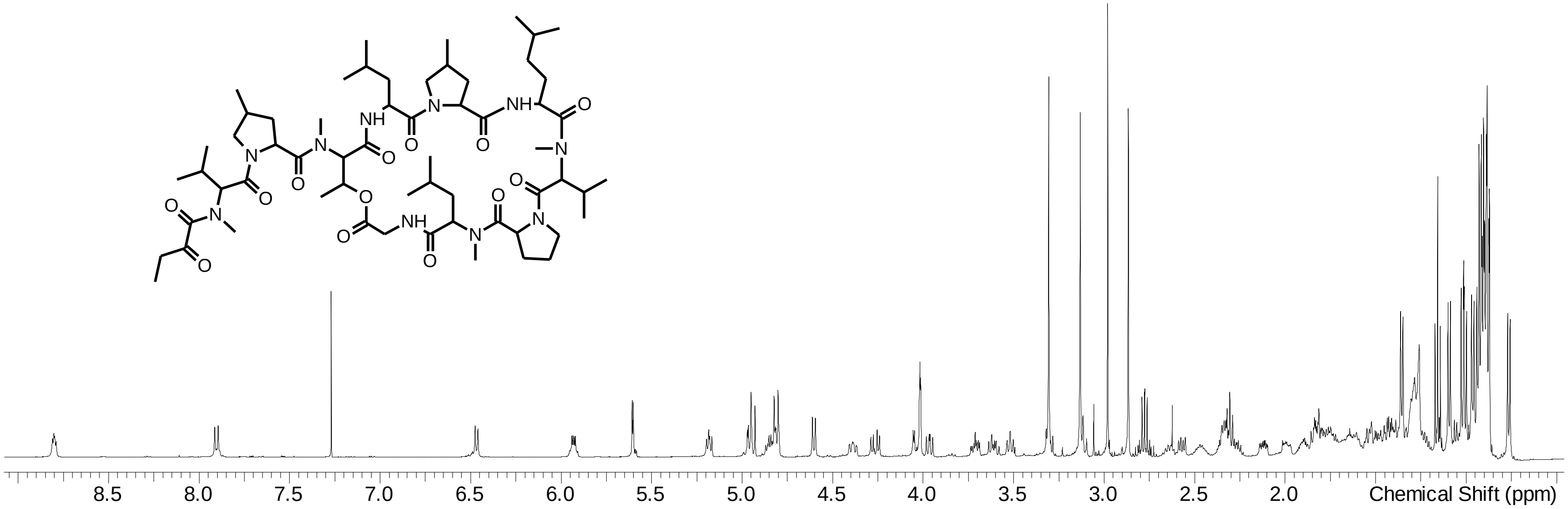

Supplementary Figure 29. ^{1}H-spectrum of MP B in $CDCl_3$ at 500 MHz.

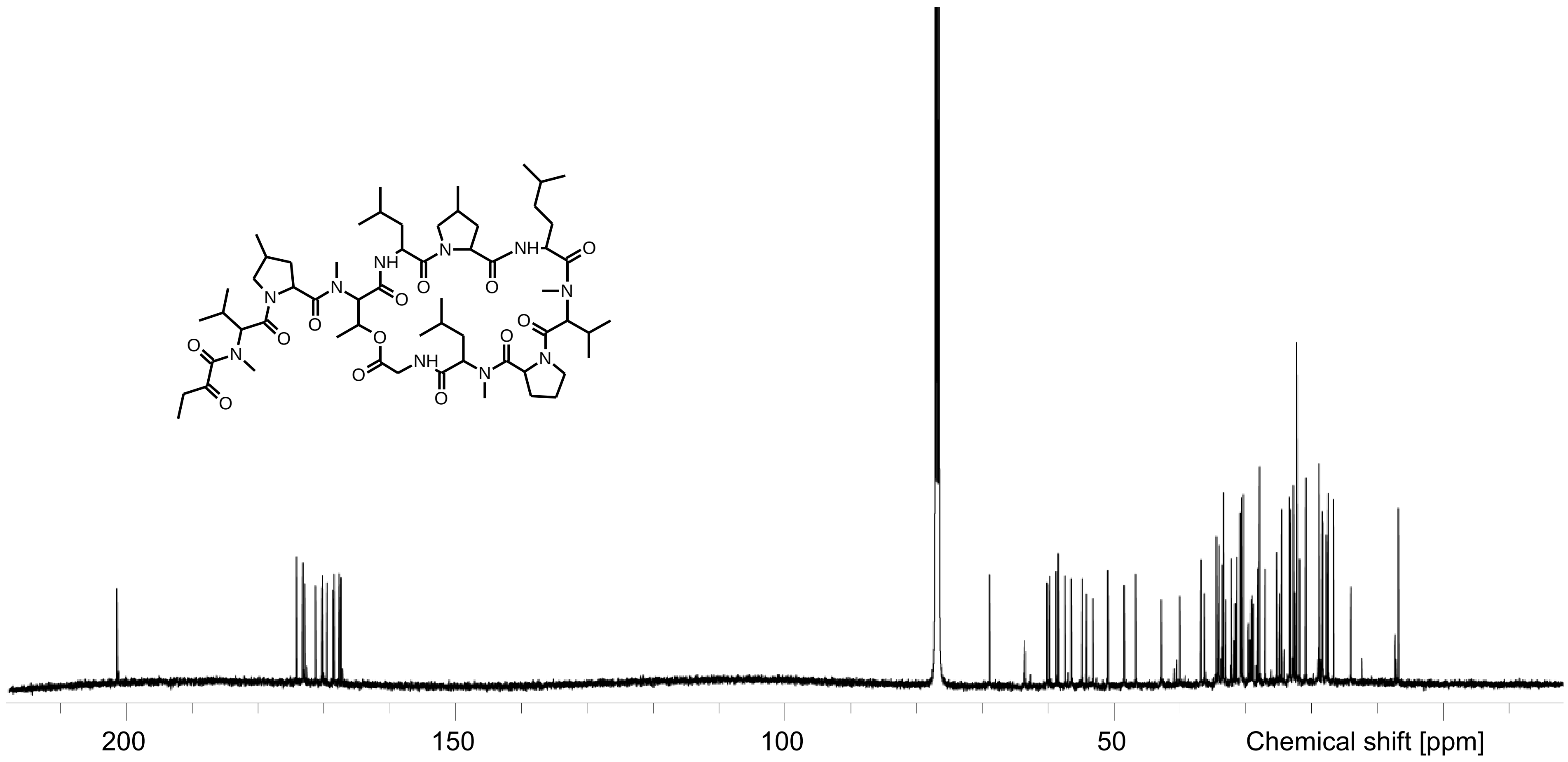

Supplementary Figure 30. 13C-spectrum of MP B in CDCl3 at 125 MHz.

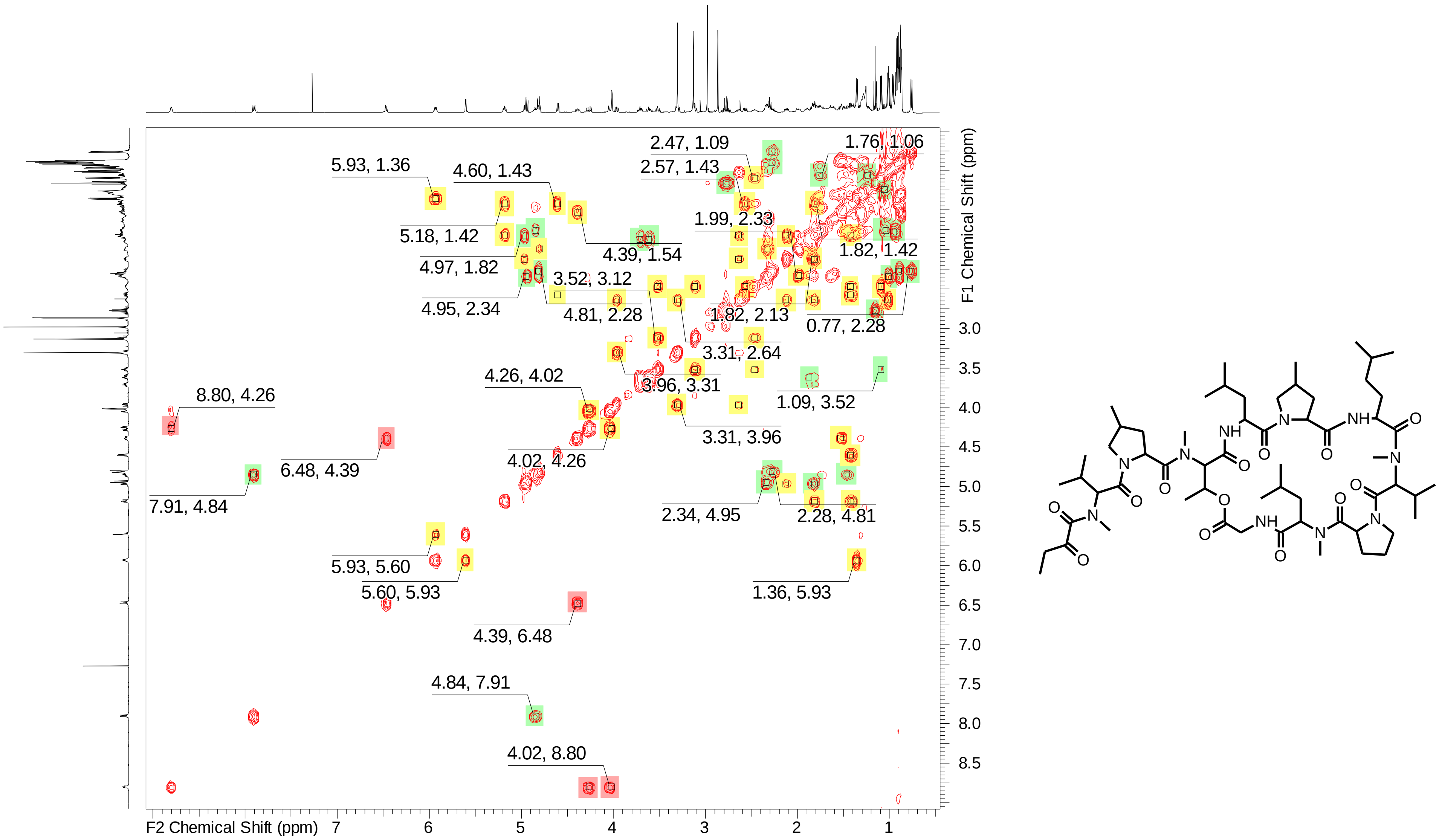

Supplementary Figure 31. COSY-spectrum of MP B in CDCl3 at 500 MHz.

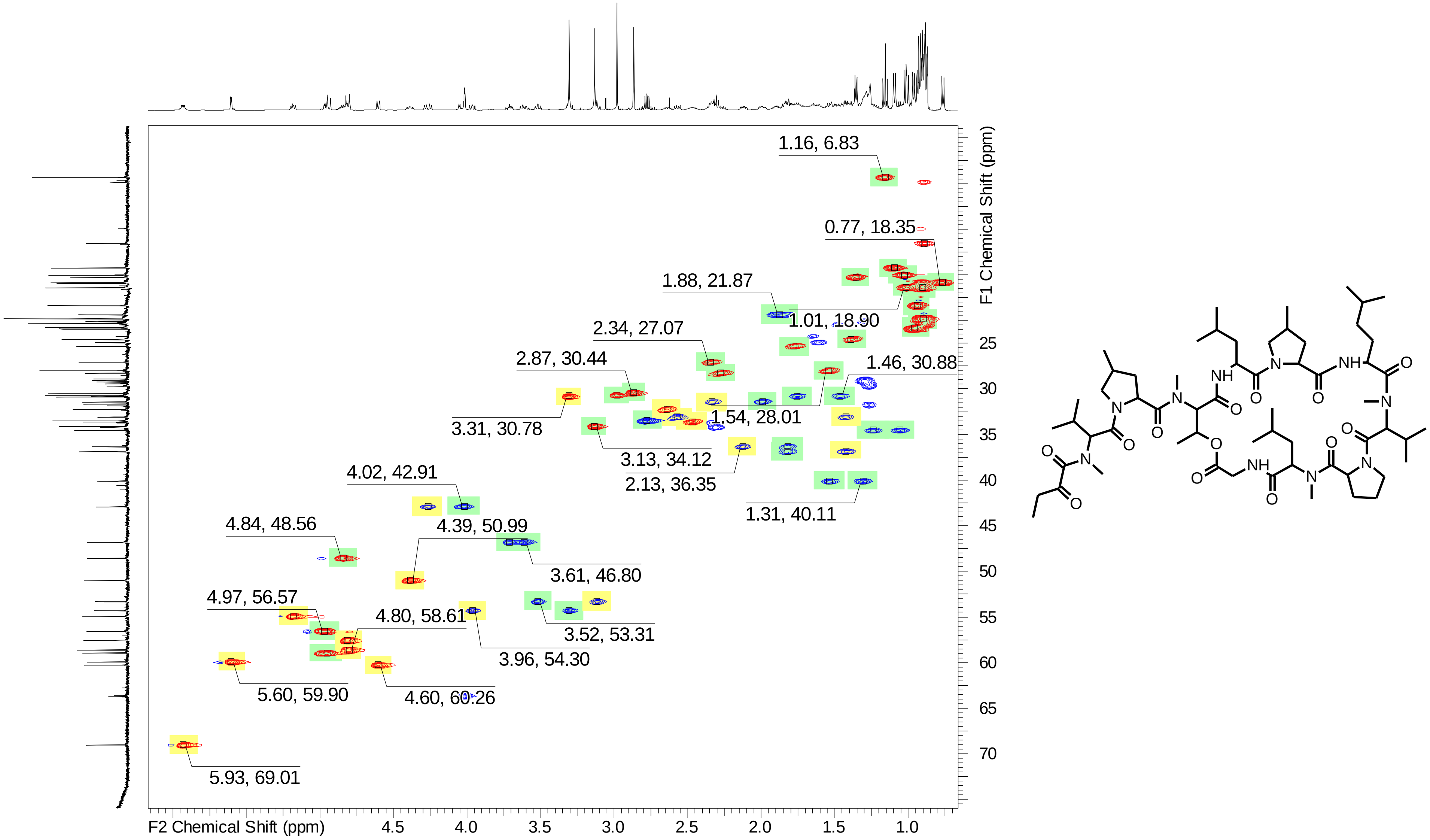

Supplementary Figure 32. HSQC-spectrum of MP B in $CDCl_3$ at 500 MHz (^{1}H)/125 MHz (^{13}C).

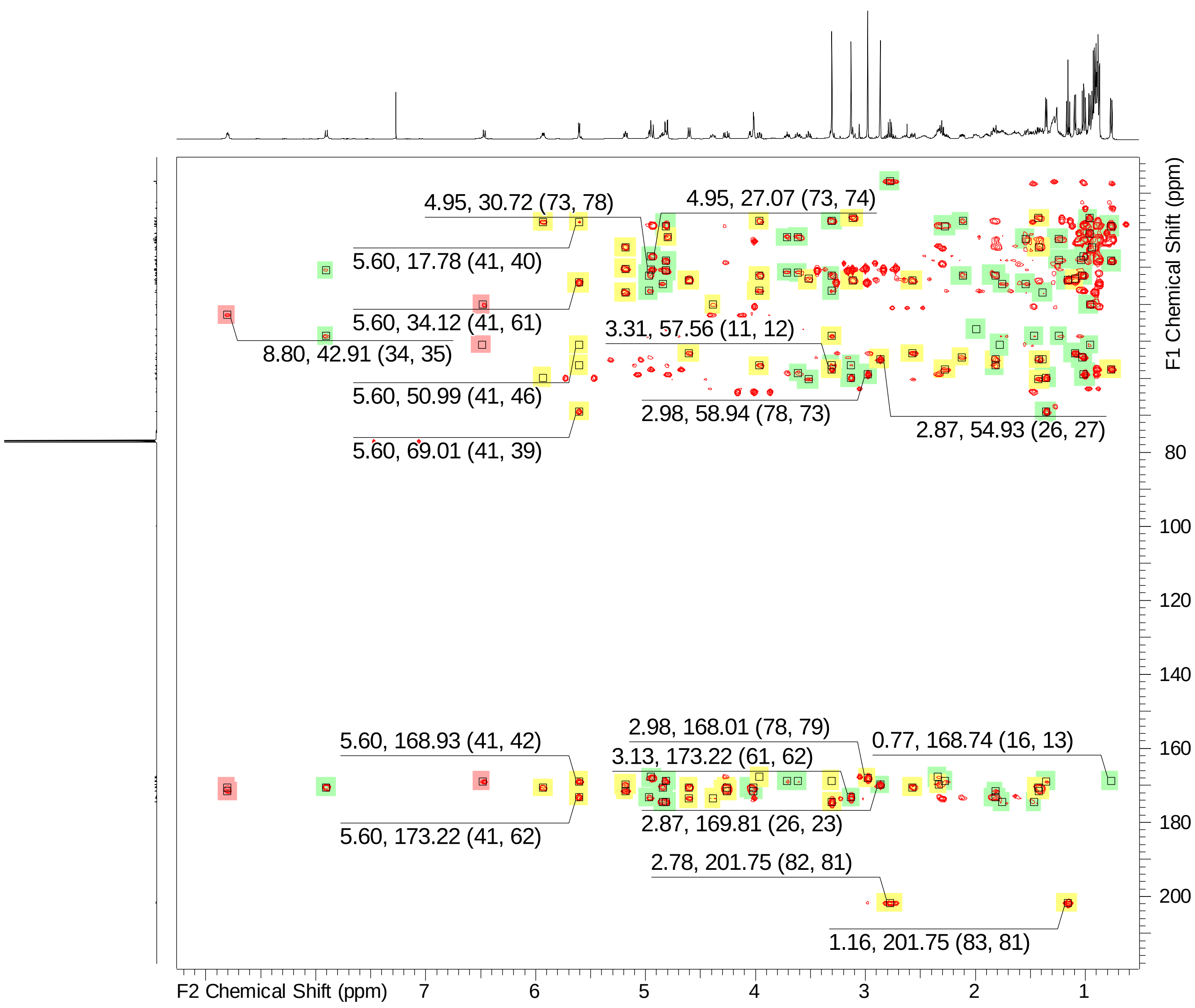

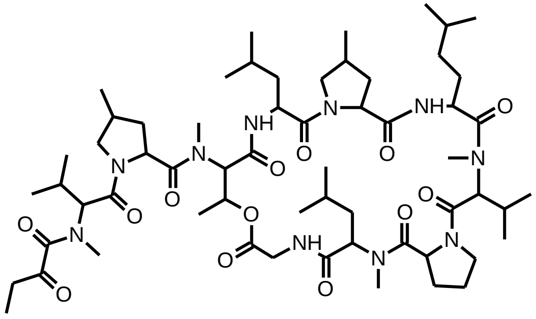

Supplementary Figure 33. HMBC-spectrum of MP B in $CDCl_3$ at 500 MHz (^1H)/125 MHz (^{13}C).

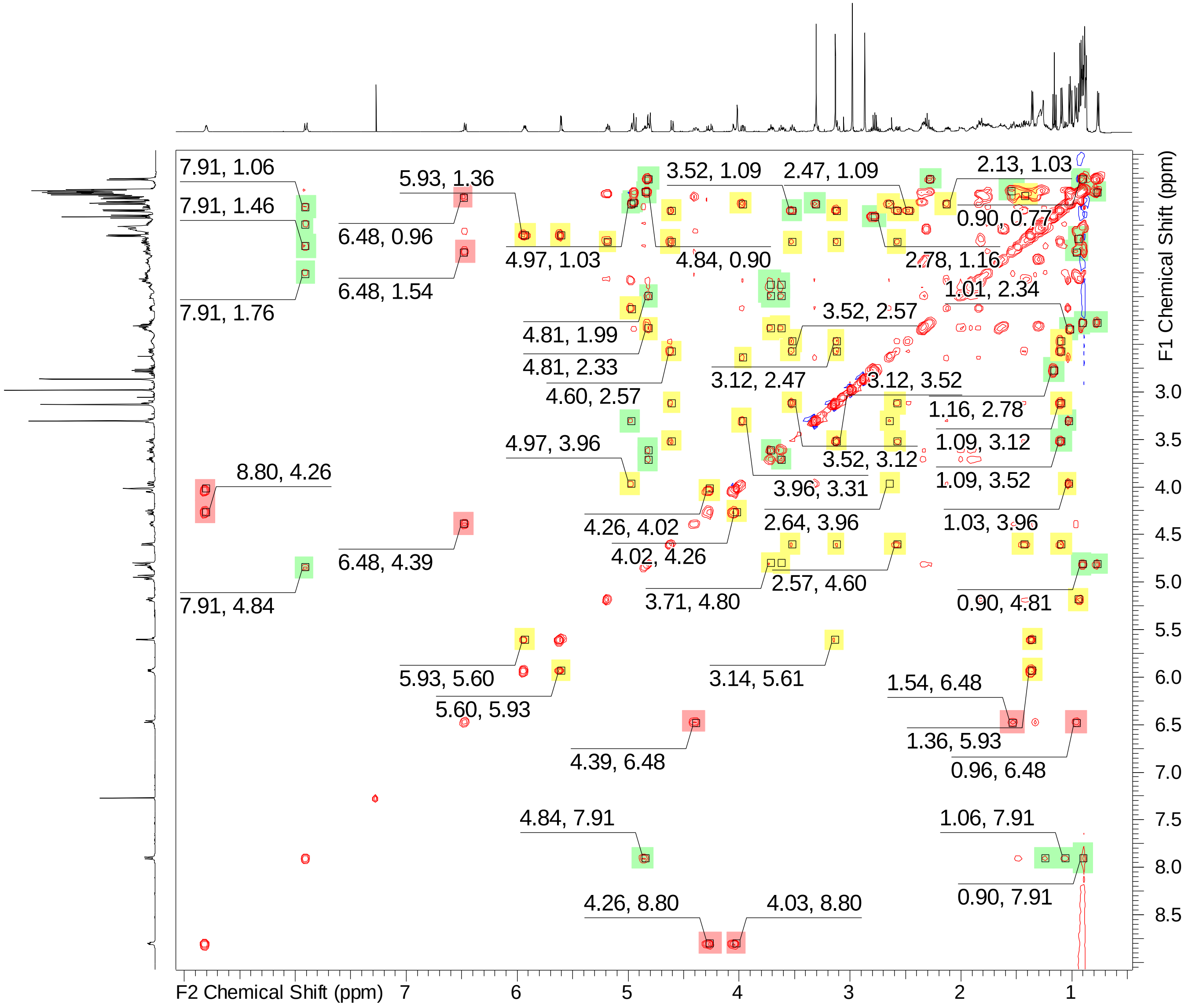

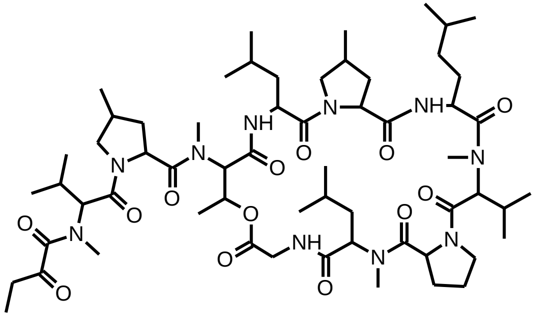

Supplementary Figure 34. TOCSY-spectrum of MP B in $CDCl_3$ at 500 MHz.

Marfey's derivatization:

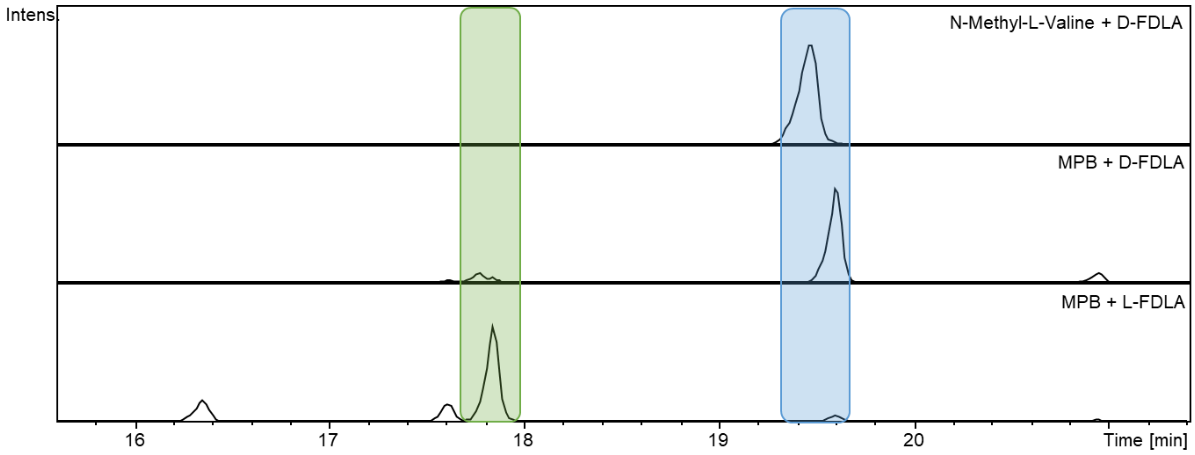

Supplementary Figure 35. Marfey's derivatization of reference N-Methyl-L-Valine with D-FDLA (upper chromatogram) and MP B with both D-FDLA (middle chromatogram) and L-FDLA (lower chromatogram). Retention time comparison represented as EICs; D-L-FDLA/L-D-FDLA highlighted in blue and L-L-FDLA/D-D-FDLA in green.

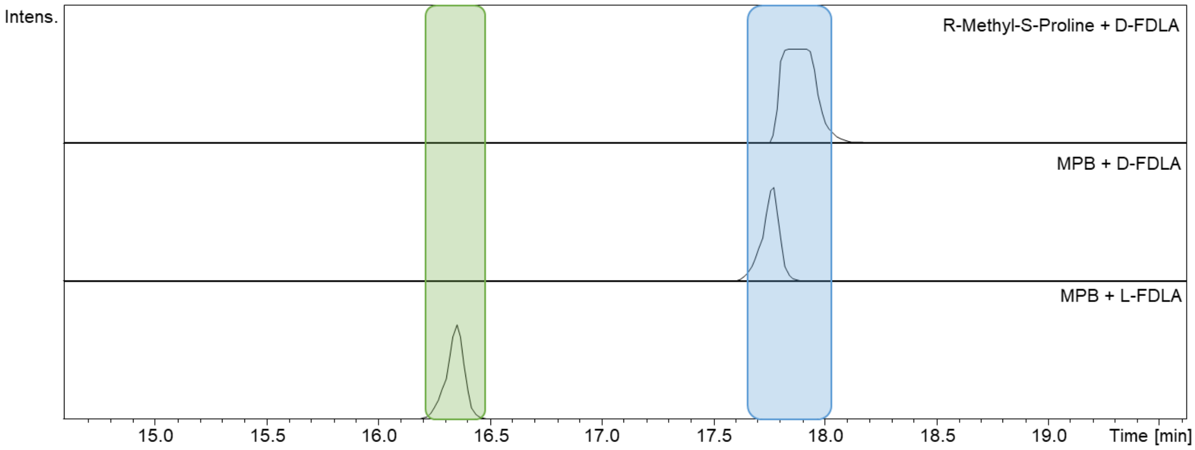

Supplementary Figure 36. Marfey's derivatization of reference R-Methyl-S-Proline with D-FDLA (upper chromatogram) and MP B with both D-FDLA (middle chromatogram) and L-FDLA (lower chromatogram). Retention time comparison represented as EICs; D-L-FDLA/L-D-FDLA highlighted in blue and L-L-FDLA/D-D-FDLA in green.

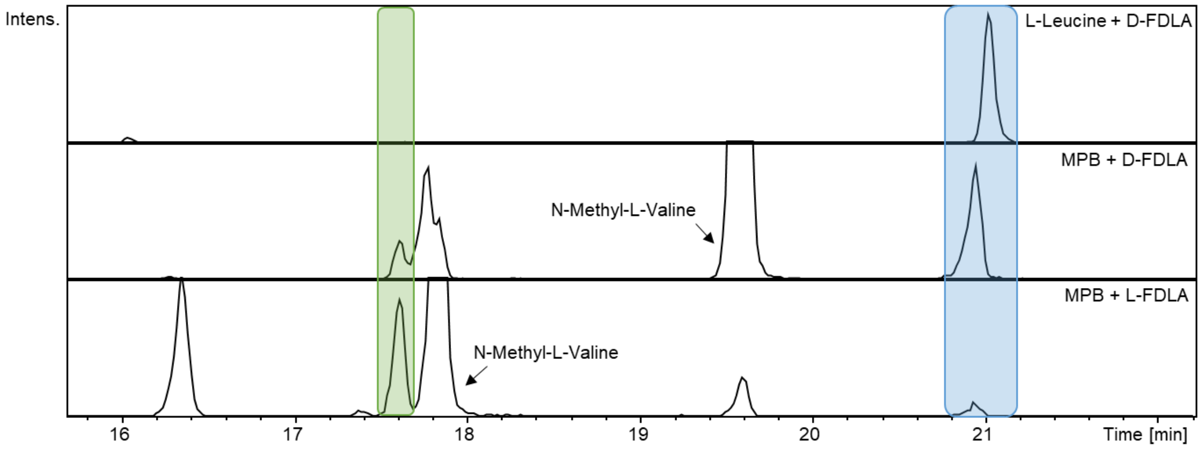

Supplementary Figure 37. Marfey's derivatization of reference L-Leucine with D-FDLA (upper chromatogram) and MP B with both D-FDLA (middle chromatogram) and L-FDLA (lower chromatogram). Retention time comparison represented as EICs; D-L-FDLA/L-D-FDLA highlighted in blue and L-L-FDLA/D-D-FDLA in green.

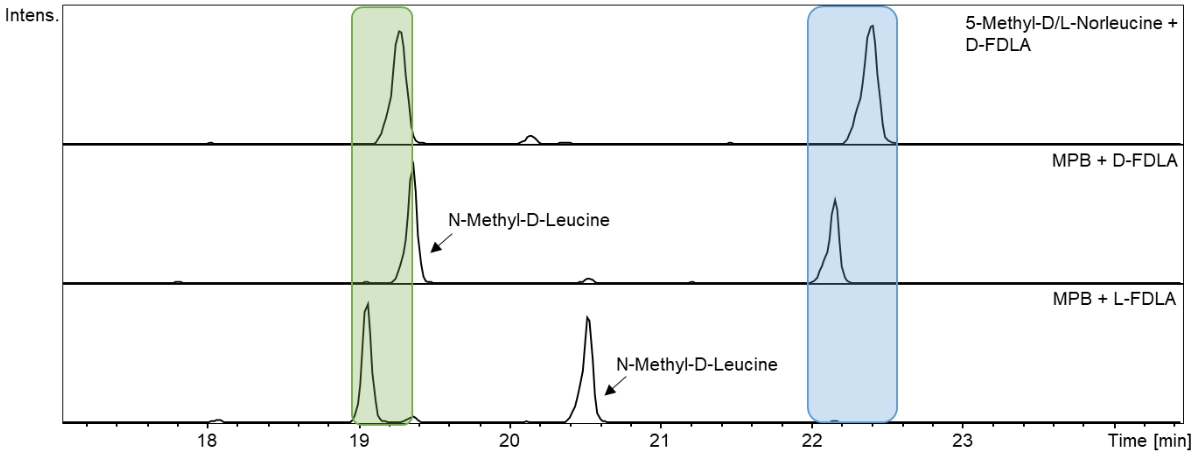

Supplementary Figure 38. Marfey's derivatization of reference 5-Methyl-D/L-Norleucine with D-FDLA (upper chromatogram) and MP B with both D-FDLA (middle chromatogram) and L-FDLA (lower chromatogram). Retention time comparison represented as EICs; D-L-FDLA/L-D-FDLA highlighted in blue and L-L-FDLA/D-D-FDLA in green.

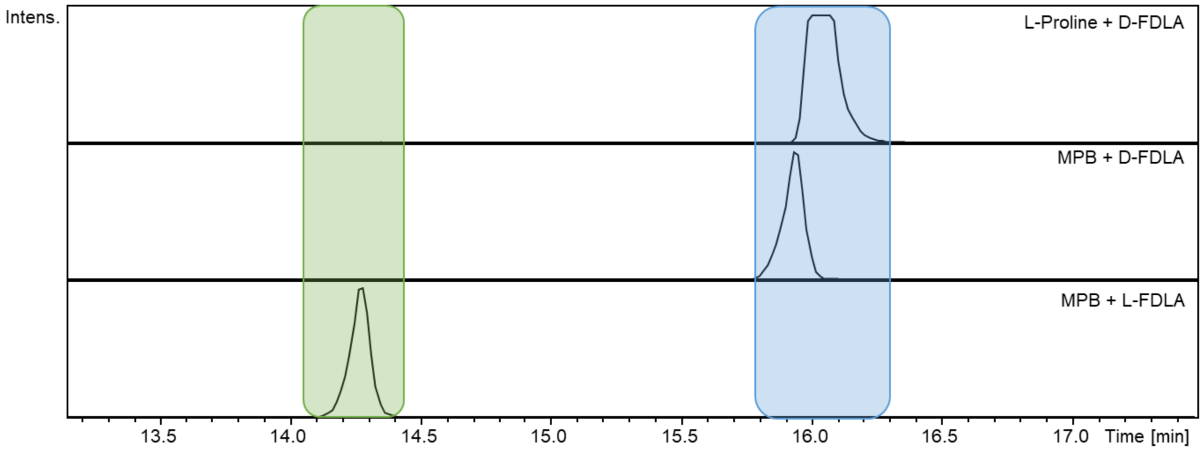

Supplementary Figure 39. Marfey's derivatization of reference L-Proline with D-FDLA (upper chromatogram) and MP B with both D-FDLA (middle chromatogram) and L-FDLA (lower chromatogram). Retention time comparison represented as EICs; D-L-FDLA/L-D-FDLA highlighted in blue and L-L-FDLA/D-D-FDLA in green.

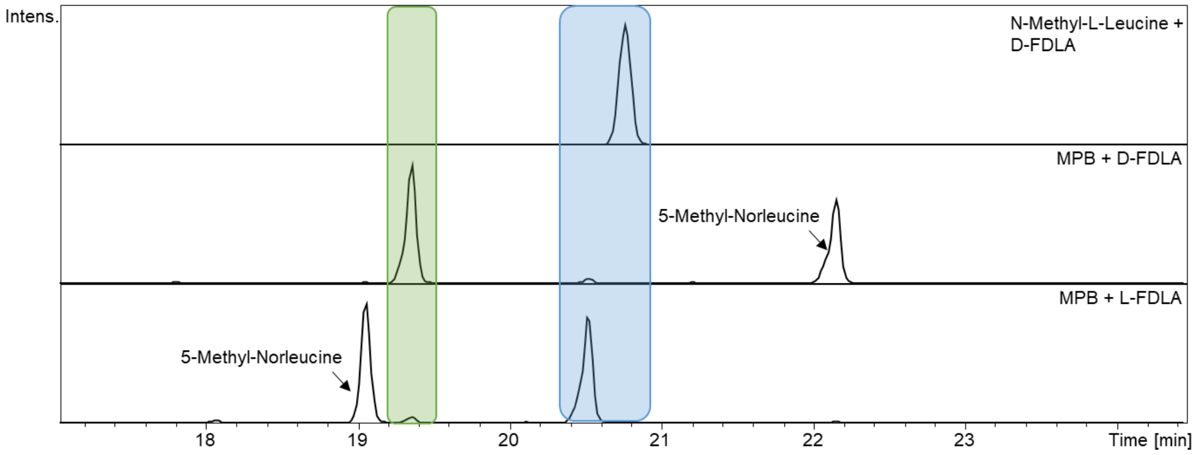

Supplementary Figure 40. Marfey's derivatization of reference N-Methyl-L-Leucine with D-FDLA (upper chromatogram) and MP B with both D-FDLA (middle chromatogram) and L-FDLA (lower chromatogram). Retention time comparison represented as EICs; D-L-FDLA/L-D-FDLA highlighted in blue and L-L-FDLA/D-D-FDLA in green.

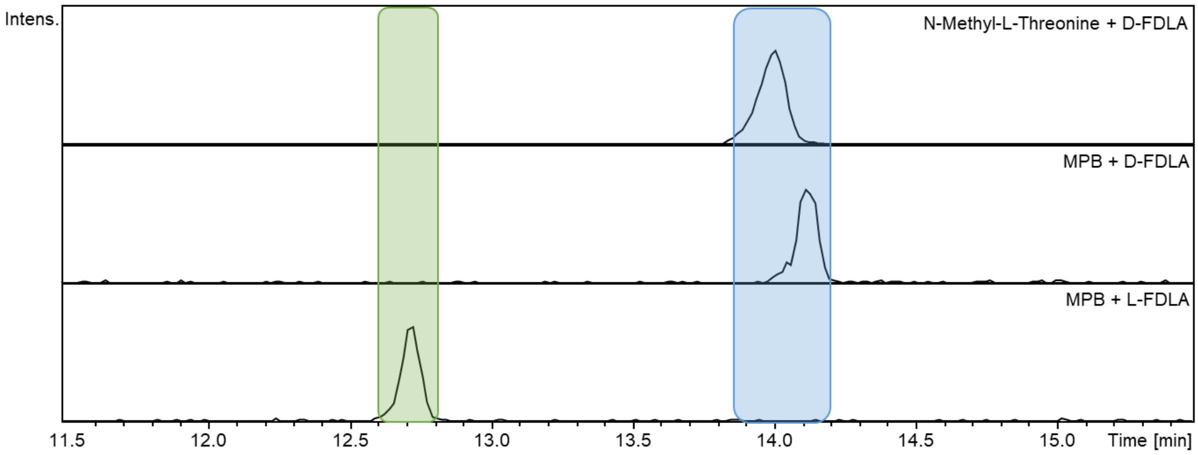

Supplementary Figure 41. Marfey's derivatization of reference N-Methyl-L-Threonine with D-FDLA (upper chromatogram) and MP B with both D-FDLA (middle chromatogram) and L-FDLA (lower chromatogram). Retention time comparison represented as EICs; D-L-FDLA/L-D-FDLA highlighted in blue and L-L-FDLA/D-D-FDLA in green.

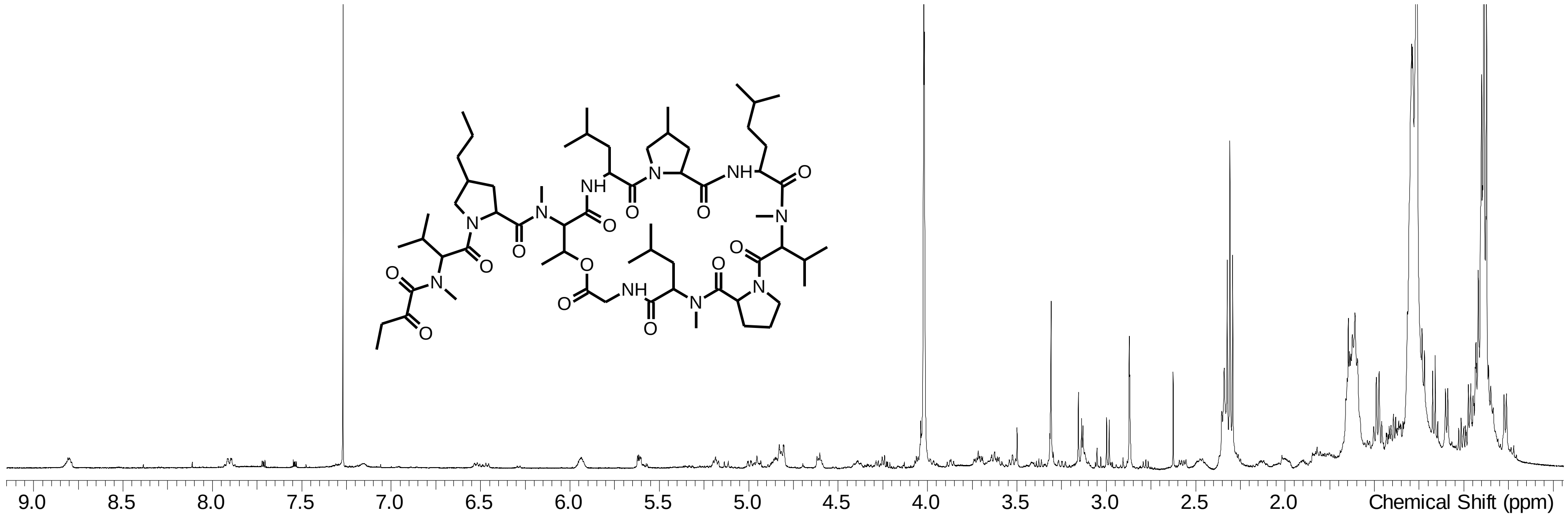

Supplementary Figure 42. ^{1}H-spectrum of MP D in $CDCl_3$ at 500 MHz.

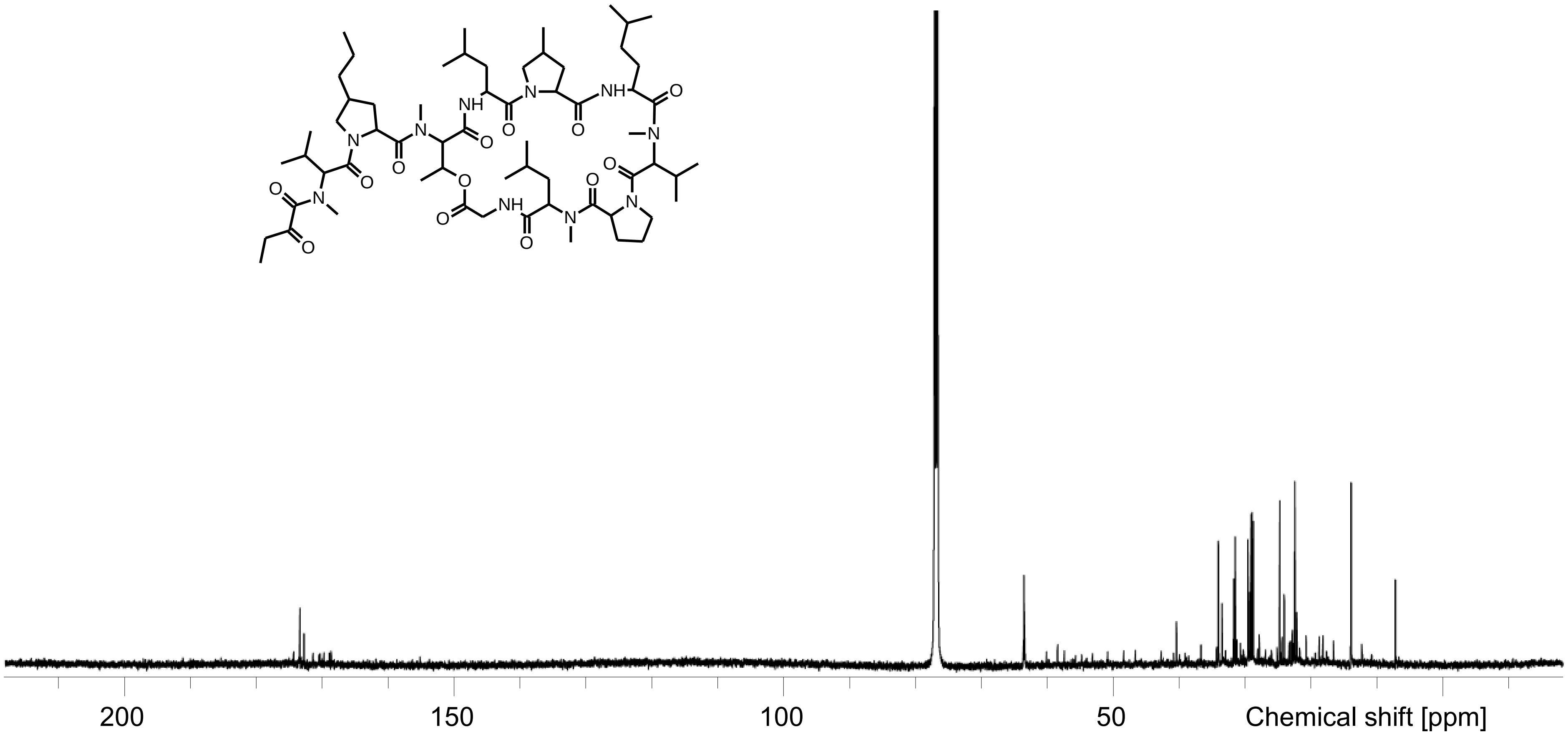

Supplementary Figure 43. ^{13}C-spectrum of MP D in $CDCl_3$ at 125 MHz.

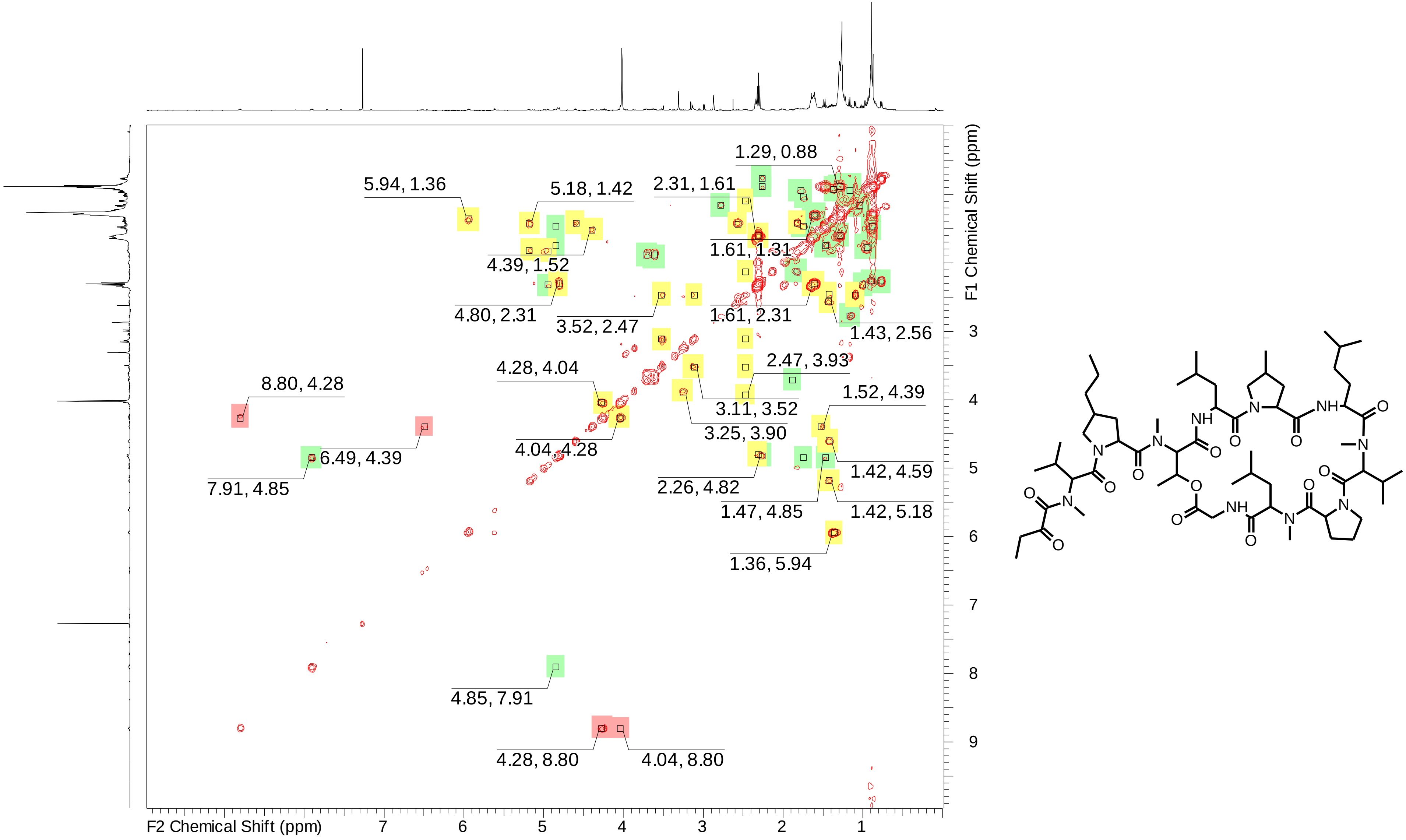

Supplementary Figure 44. COSY-spectrum of MP D in $CDCl_3$ at 500 MHz.

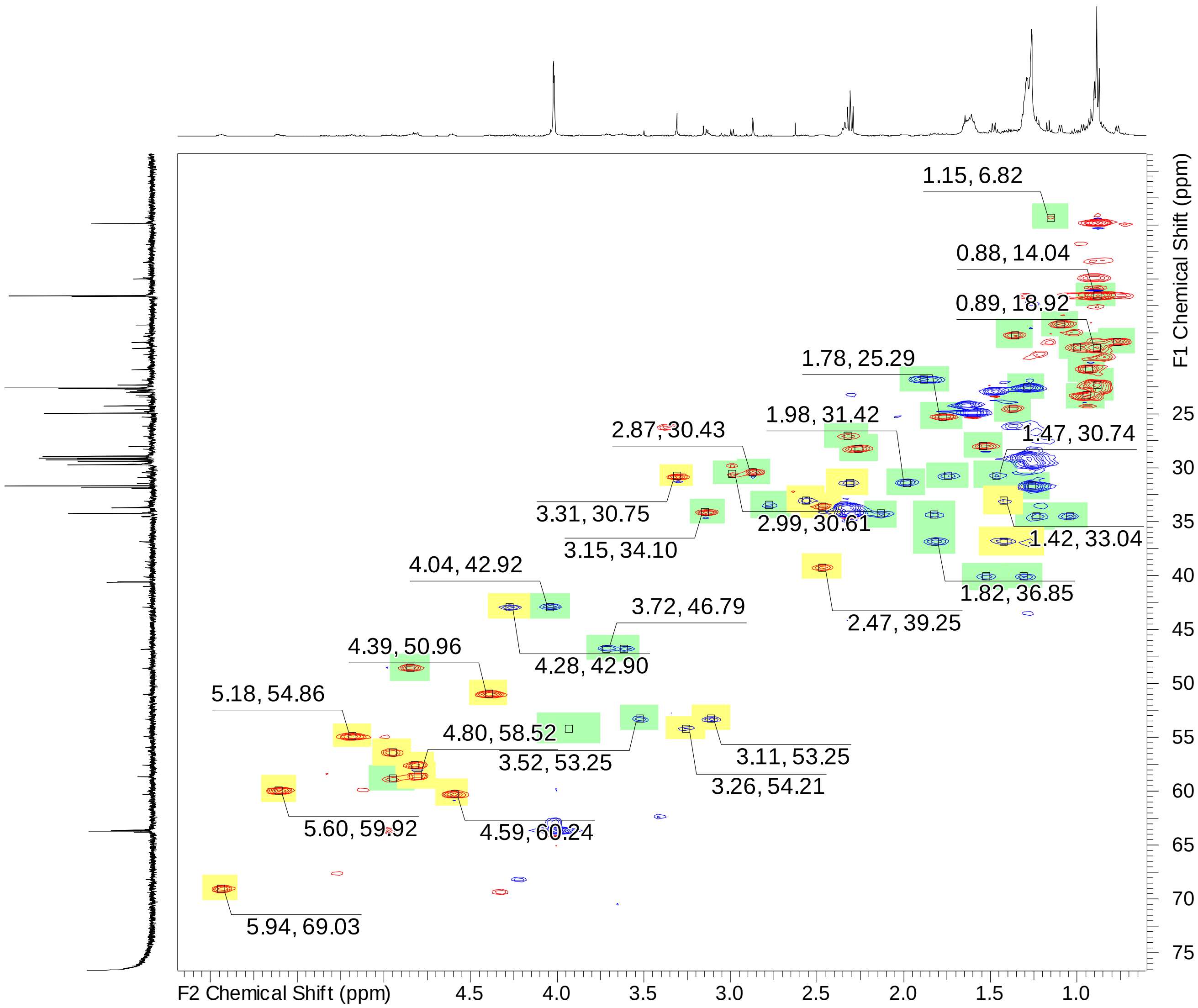

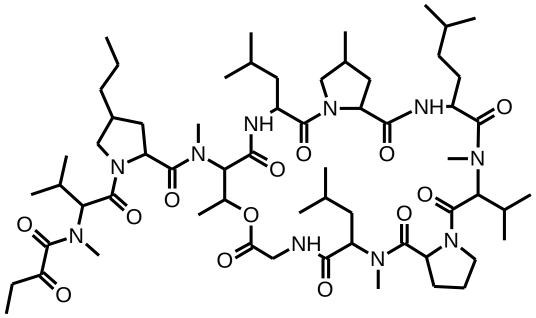

Supplementary Figure 45. HSQC-spectrum of MP D in CDCl3 at 500 MHz (1H)/125 MHz (13C).

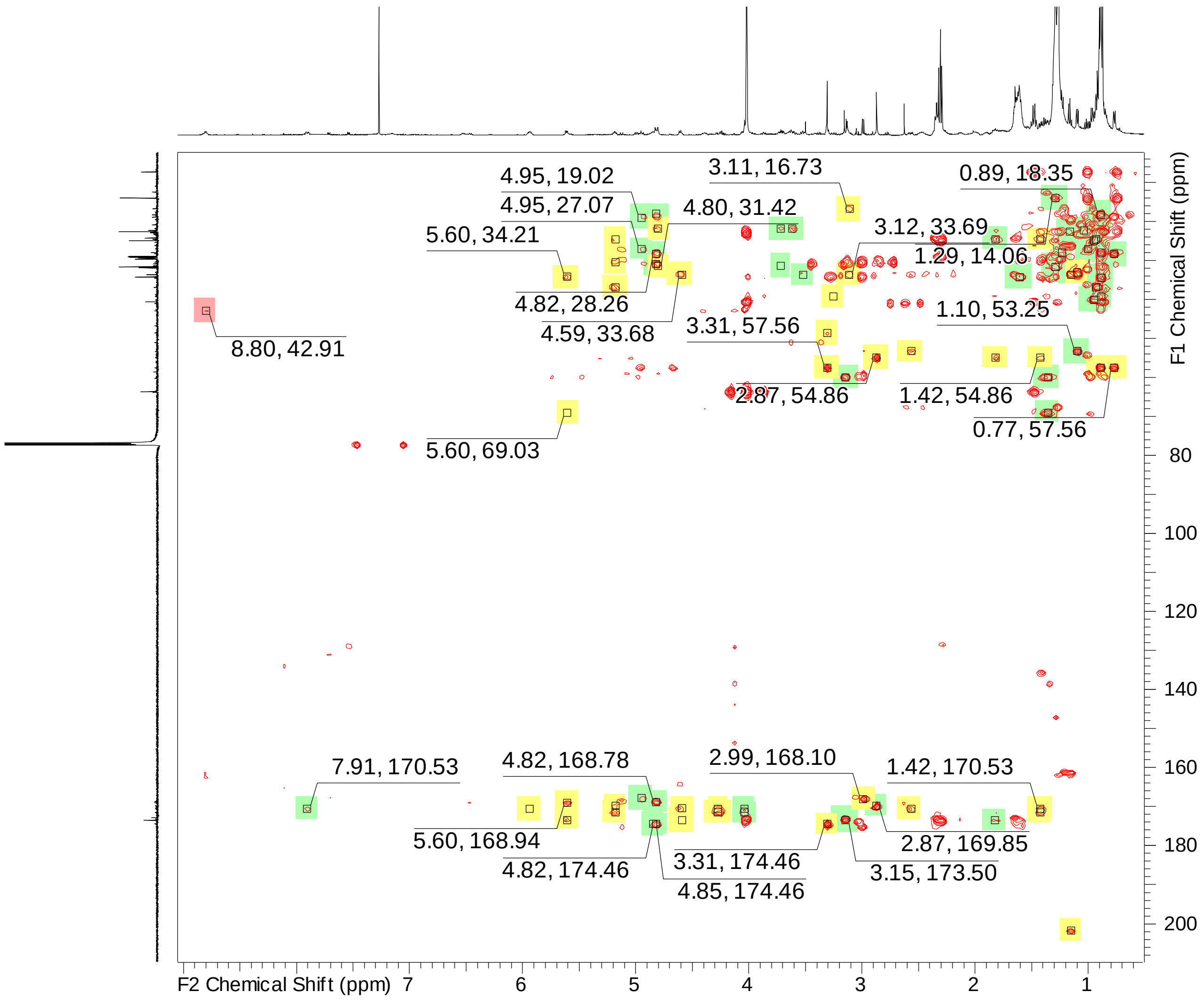

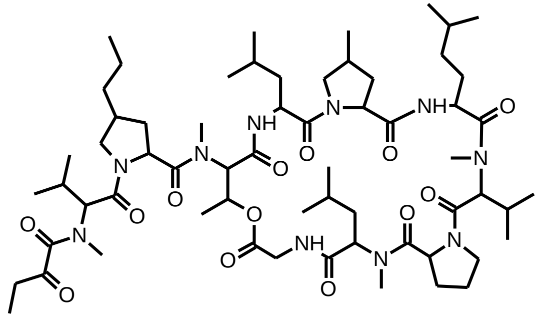

Supplementary Figure 46. HMBC-spectrum of MP D in CDCl3 at 500 MHz (1H)/125 MHz (13C).

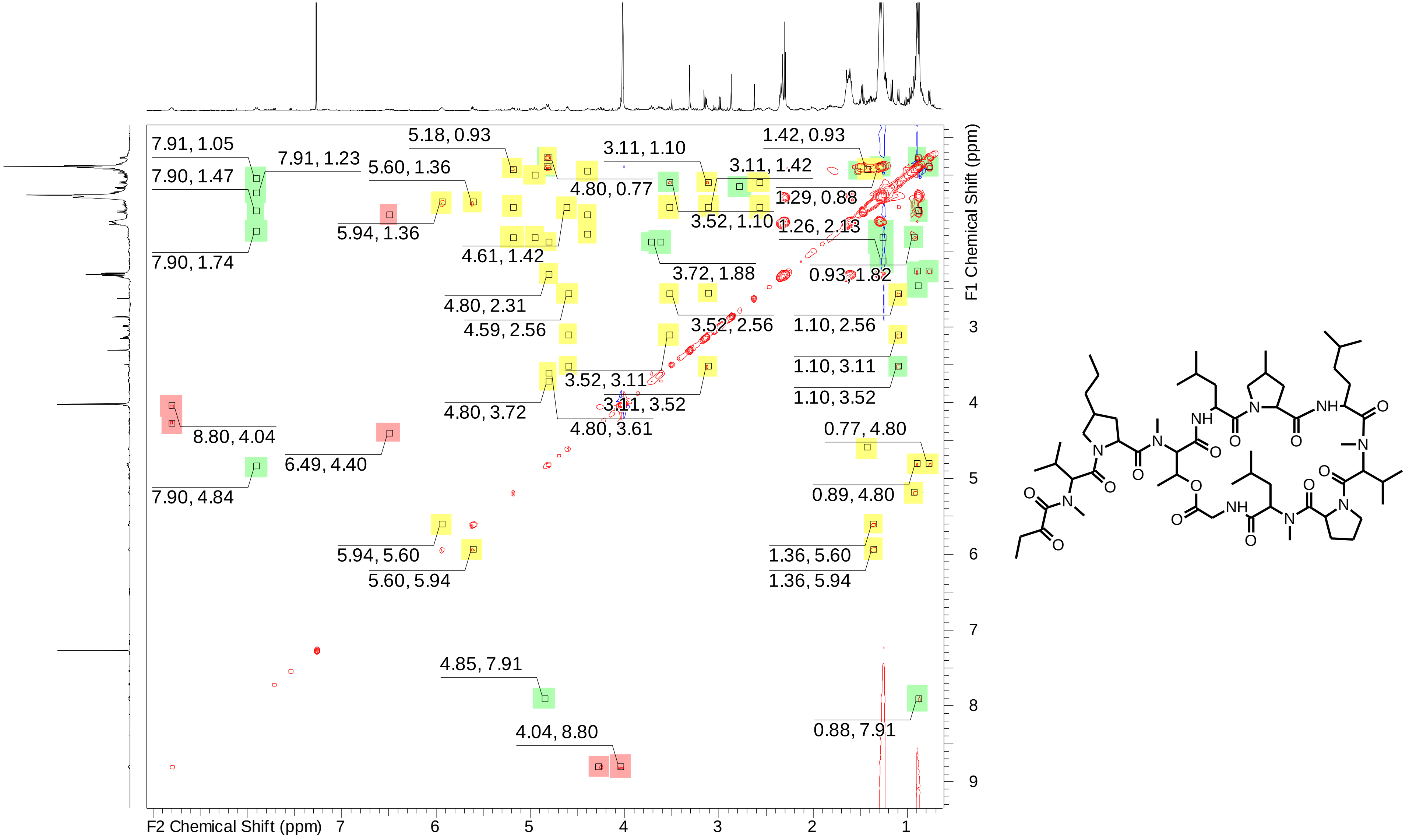

Supplementary Figure 47. TOCSY-spectrum of MP D in $CDCl_3$ at 500 MHz.

Marfey's derivatization:

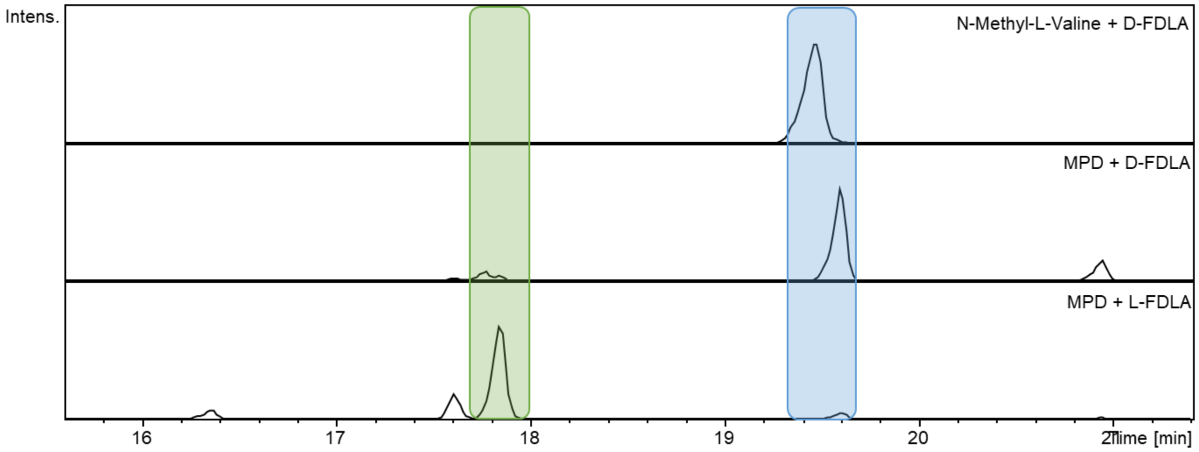

Supplementary Figure 48. Marfey's derivatization of reference N-Methyl-L-Valine (upper chromatogram) with D-FDLA and MP D with both D-FDLA (middle chromatogram) and L-FDLA (lower chromatogram). Retention time comparison represented as EICs; D-L-FDLA/L-D-FDLA highlighted in blue and L-L-FDLA/D-D-FDLA in green.

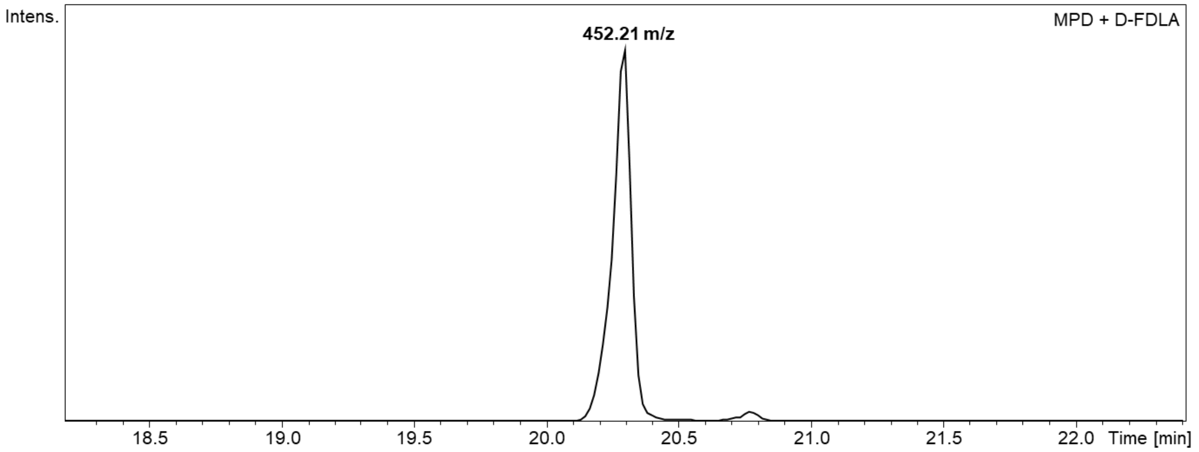

Supplementary Figure 49. Marfey's derivatization of MP D with D-FDLA. Represented as EICs (452.21 m/z), no reference Propyl-L-Proline was available.

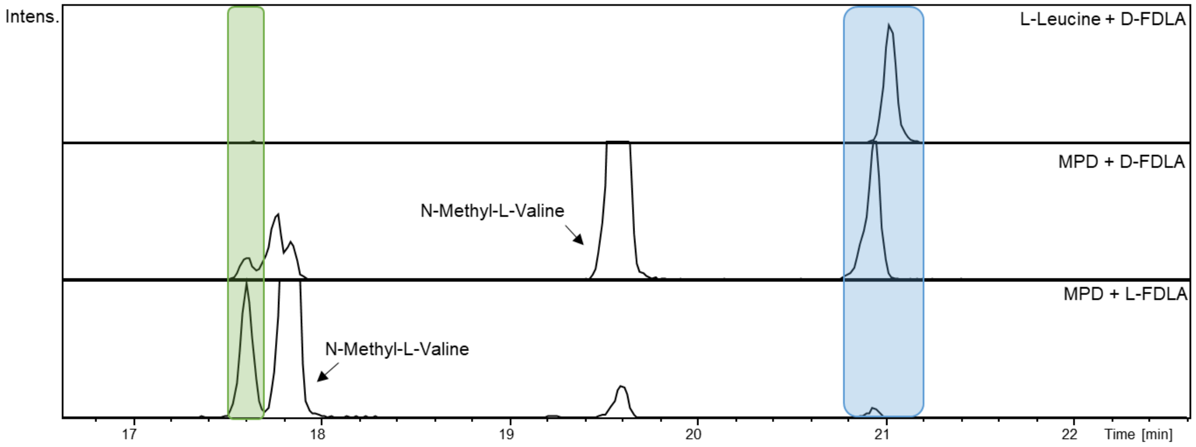

Supplementary Figure 50. Marfey's derivatization of reference L-Leucine with D-FDLA (upper chromatogram) and MP D with both D-FDLA (middle chromatogram) and L-FDLA (lower chromatogram). Retention time comparison represented as EICs; D-L-FDLA/L-D-FDLA highlighted in blue and L-L-FDLA/D-D-FDLA in green.

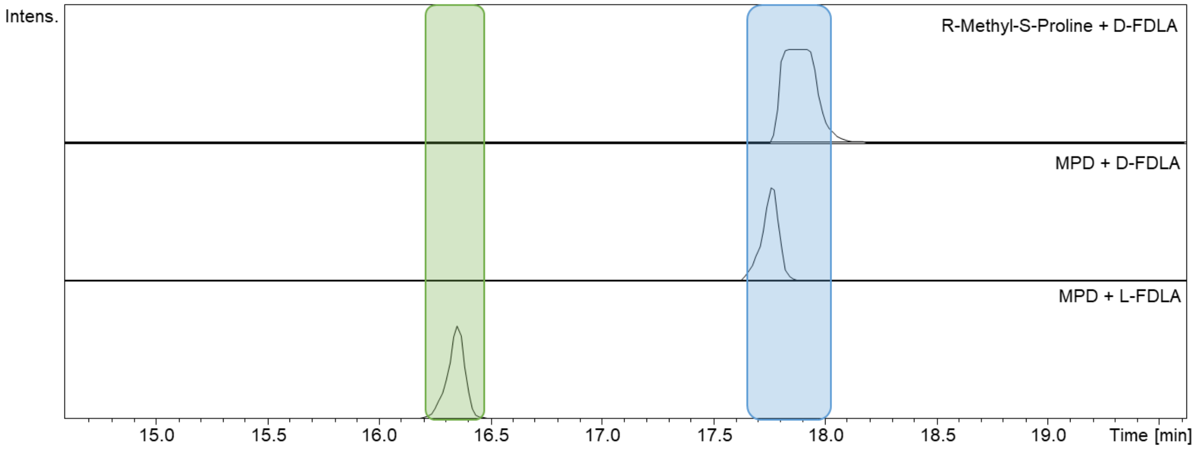

Supplementary Figure 51. Marfey's derivatization of reference R-Methyl-S-Proline (upper chromatogram) with D-FDLA and MP D with both D-FDLA (middle chromatogram) and L-FDLA (lower chromatogram). Retention time comparison represented as EICs; D-L-FDLA/L-D-FDLA highlighted in blue and L-L-FDLA/D-D-FDLA in green.

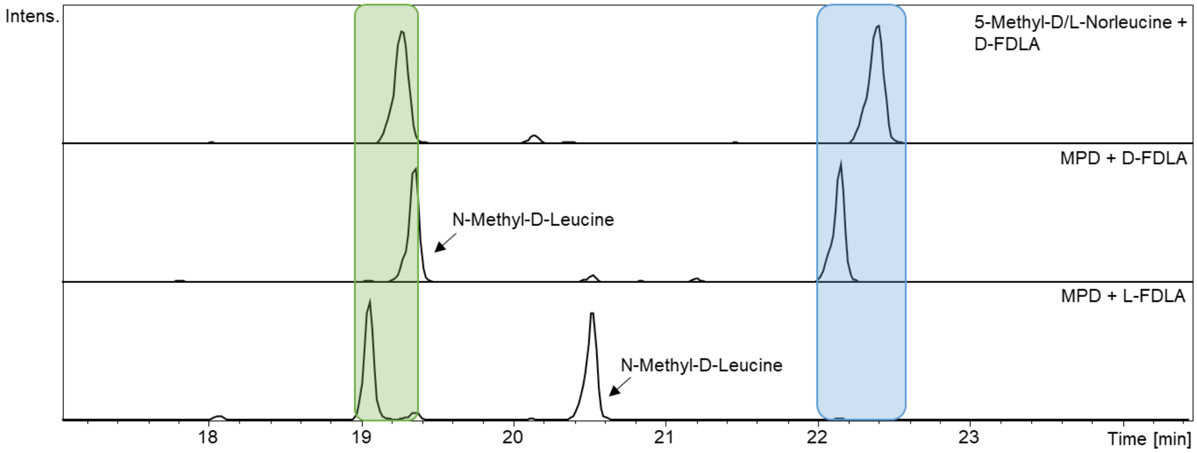

Supplementary Figure 52. Marfey's derivatization of reference 5-Methyl-D/L-Norleucine with D-FDLA (upper chromatogram) and MP D with both D-FDLA (middle chromatogram) and L-FDLA (lower chromatogram). Retention time comparison represented as EICs; D-L-FDLA/L-D-FDLA highlighted in blue and L-L-FDLA/D-D-FDLA in green.

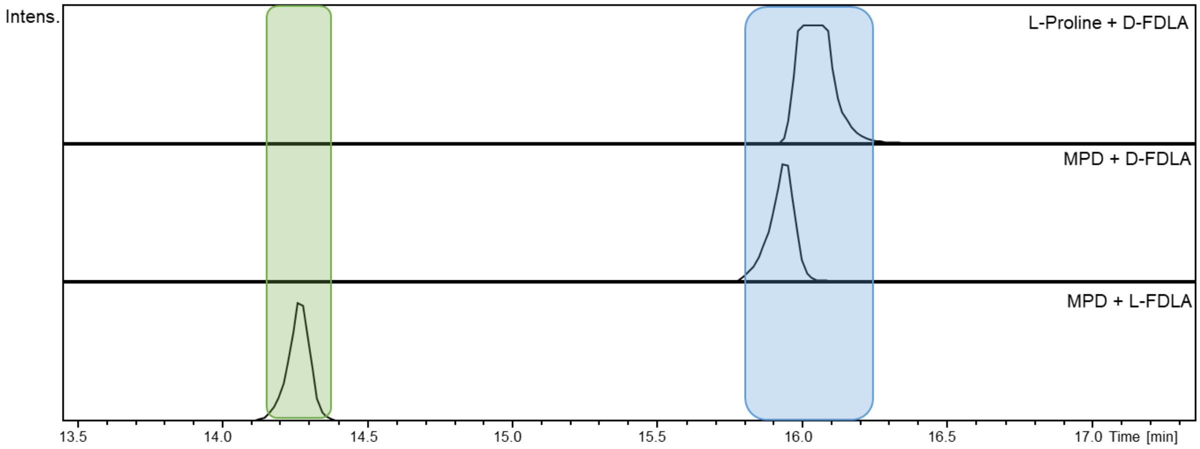

Supplementary Figure 53. Marfey's derivatization of reference L-Proline (upper chromatogram) with D-FDLA and MP D with both D-FDLA (middle chromatogram) and L-FDLA (lower chromatogram). Retention time comparison represented as EICs; D-L-FDLA/L-D-FDLA highlighted in blue and L-L-FDLA/D-D-FDLA in green.

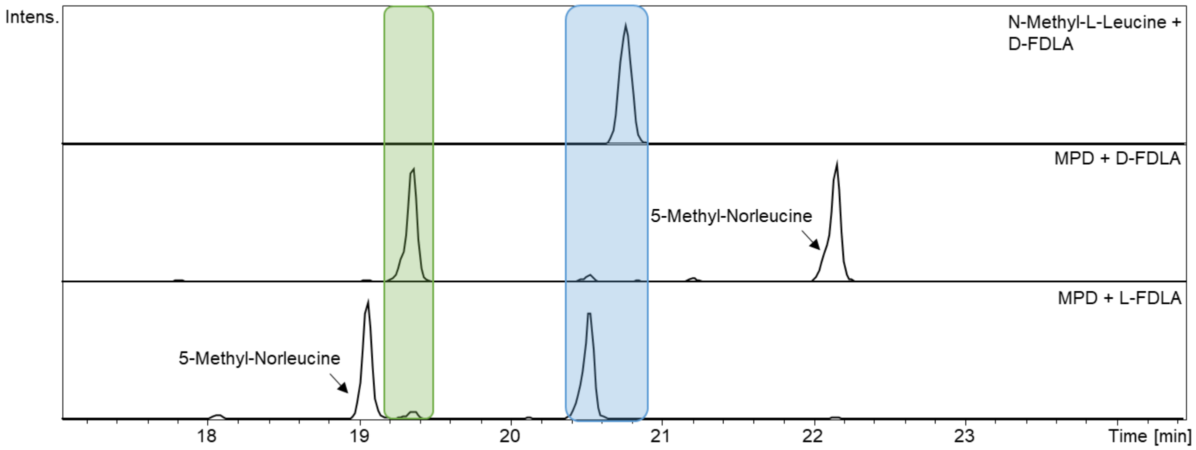

Supplementary Figure 54. Marfey's derivatization of reference N-Methyl-L-Leucine with D-FDLA (upper chromatogram) and MP D with both D-FDLA (middle chromatogram) and L-FDLA (lower chromatogram). Retention time comparison represented as EICs; D-L-FDLA/L-D-FDLA highlighted in blue and L-L-FDLA/D-D-FDLA in green.

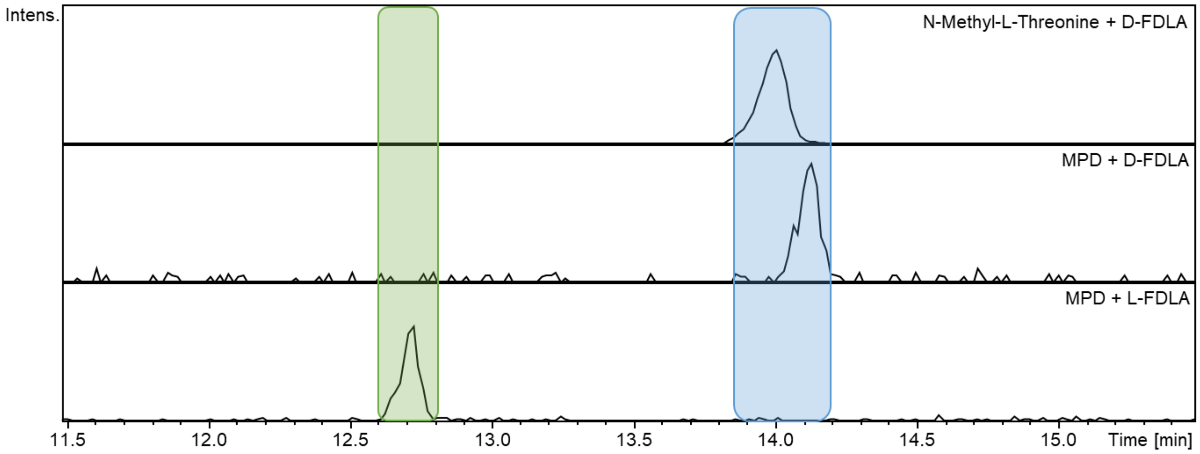

Supplementary Figure 55. Marfey's derivatization of reference N-Methyl-L-Threonine with D-FDLA (upper chromatogram) and MP D with both D-FDLA (middle chromatogram) and L-FDLA (lower chromatogram). Retention time comparison represented as EICs; D-L-FDLA/L-D-FDLA highlighted in blue and L-L-FDLA/D-D-FDLA in green.

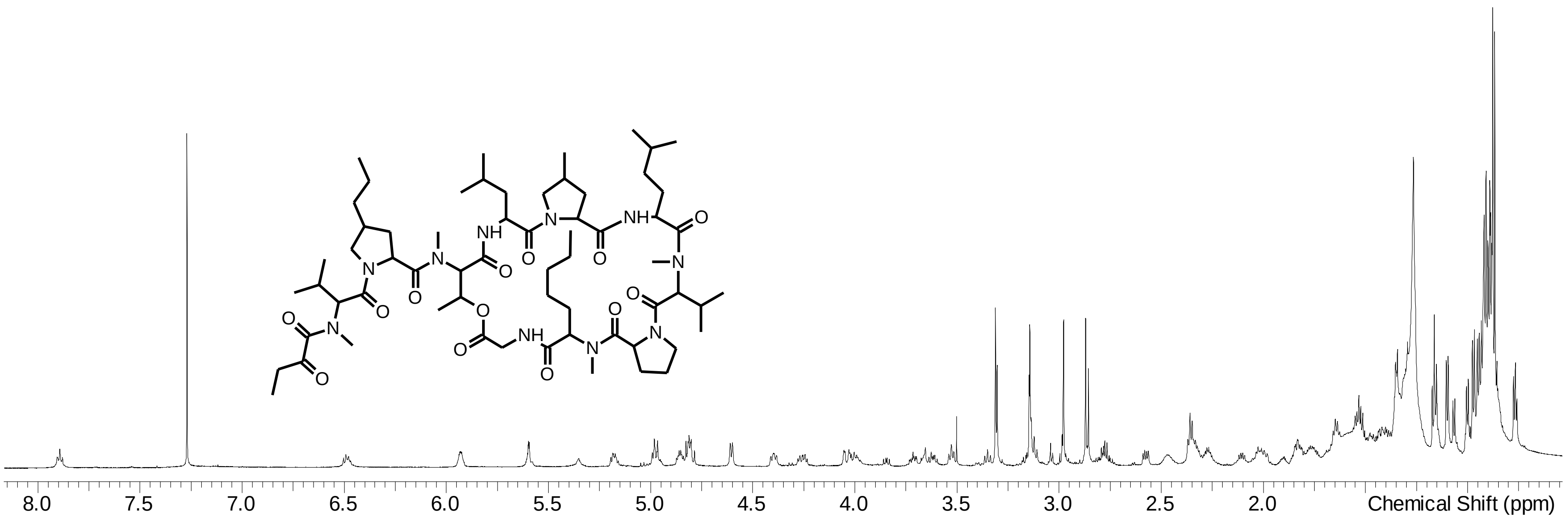

Supplementary Figure 56. 1H-spectrum of MP E in CDCl3 at 700 MHz.

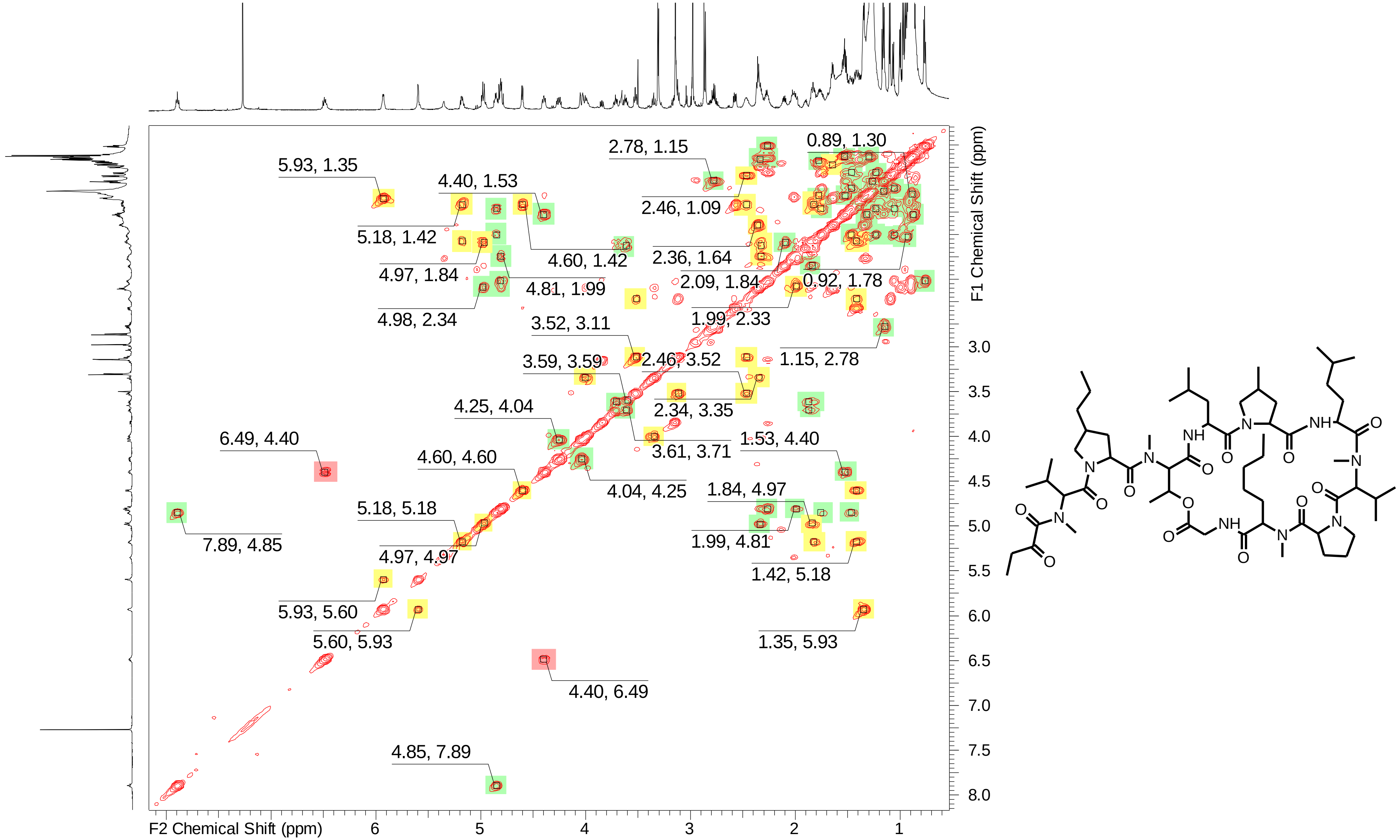

Supplementary Figure 57. COSY-spectrum of MP E in CDCl3 at 700 MHz.

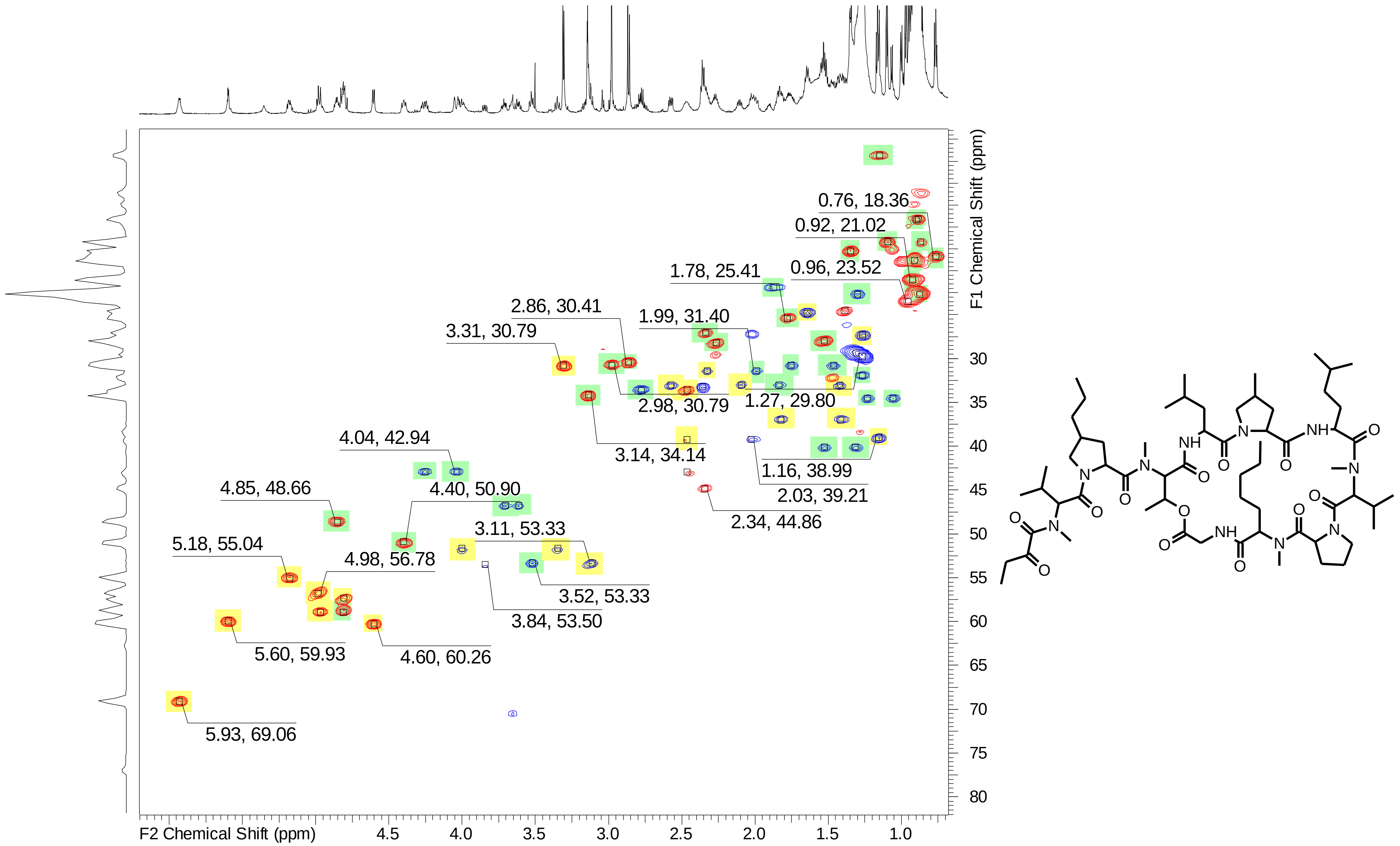

Supplementary Figure 58. HSQC-spectrum of MP E in CDCl3 at 700 MHz (1H)/175 MHz (13C).

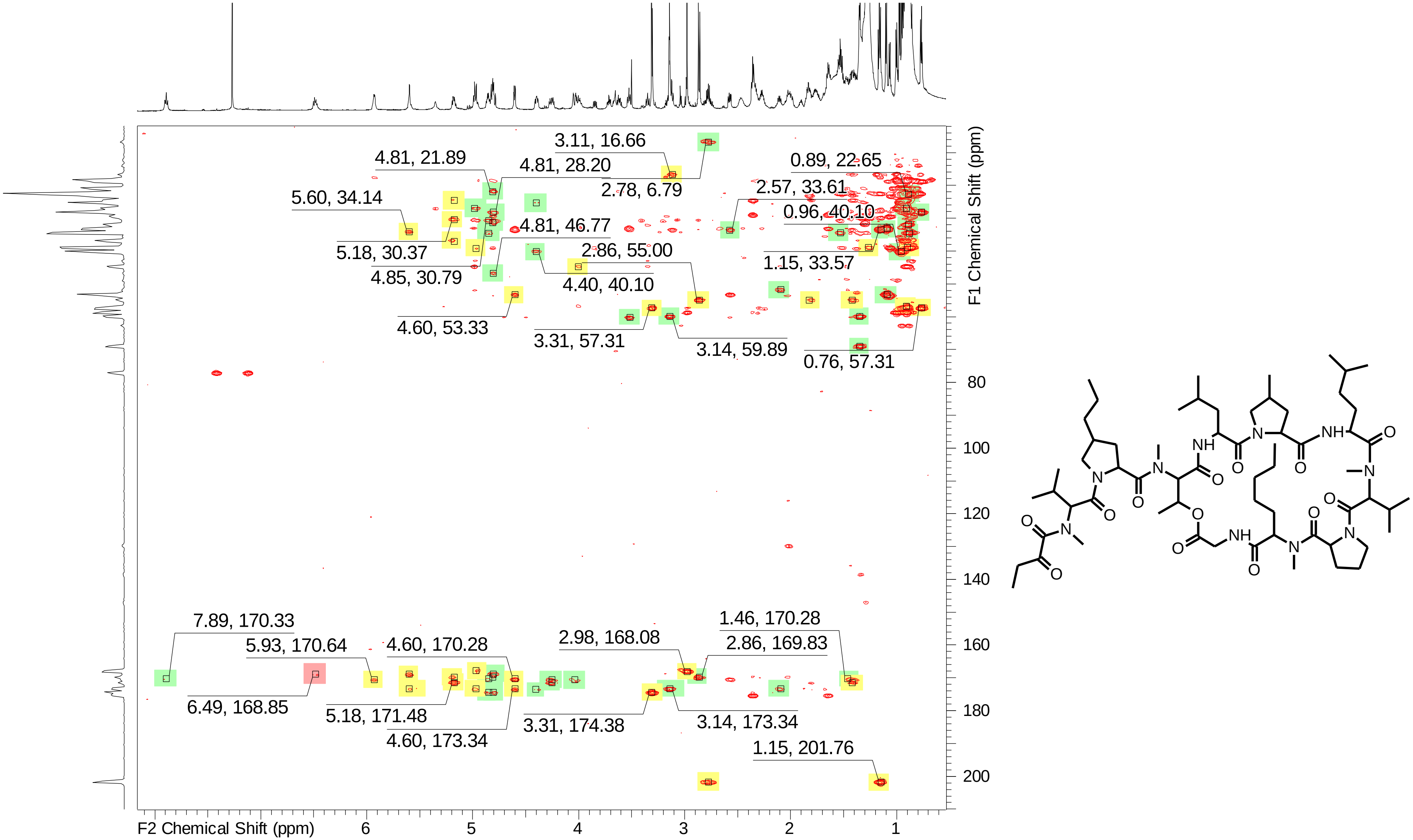

Supplementary Figure 59. HMBC-spectrum of MP E in CDCl3 at 700 MHz (1H)/175 MHz (13C).

Marfey's derivatization:

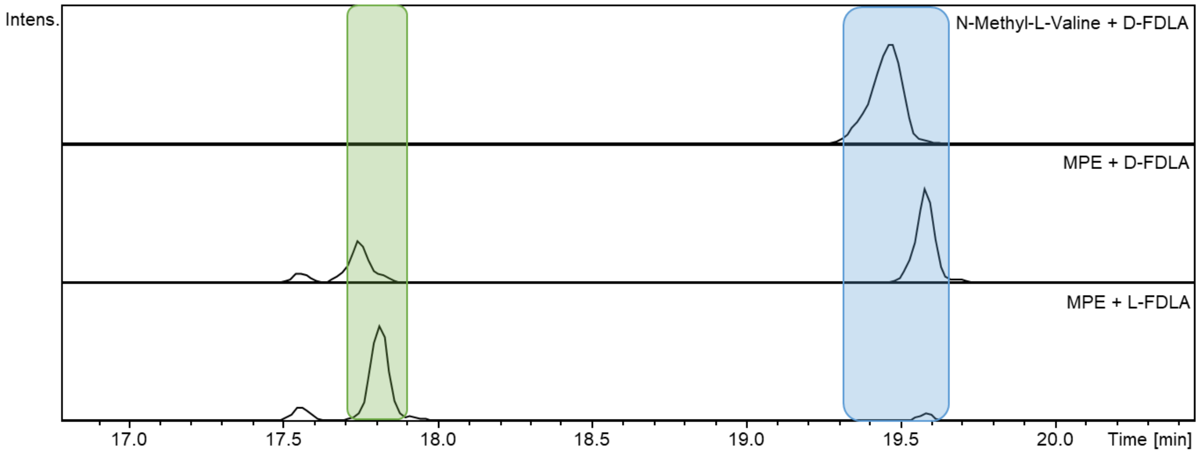

Supplementary Figure 60. Marfey's derivatization of reference N-Methyl-L-Valine (upper chromatogram) with D-FDLA and MP E with both D-FDLA (middle chromatogram) and L-FDLA (lower chromatogram). Retention time comparison represented as EICs; D-L-FDLA/L-D-FDLA highlighted in blue and L-L-FDLA/D-D-FDLA in green.

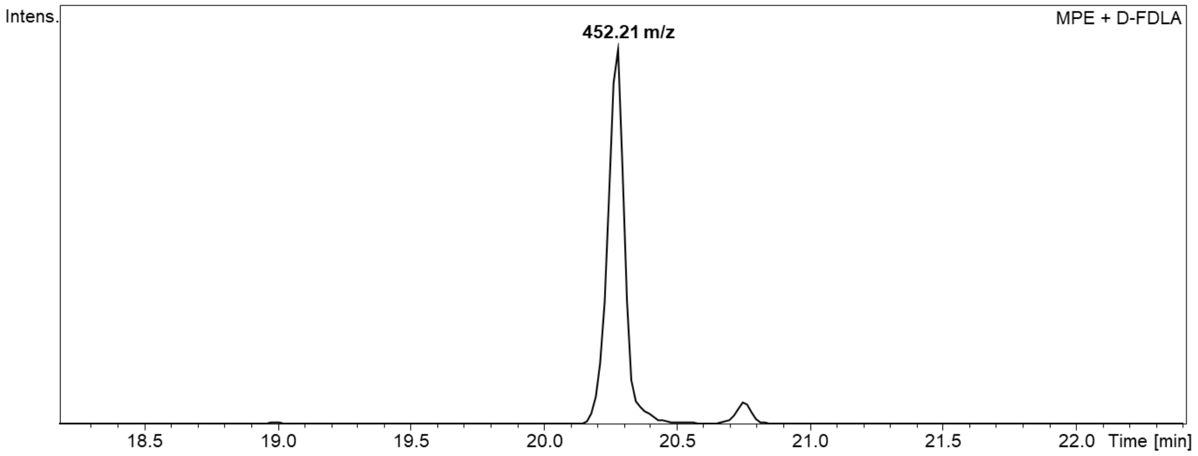

Supplementary Figure 61. Marfey's derivatization of MP E with D-FDLA. Represented as EICs (452.21 m/z), no reference Propyl-L-Proline was available.

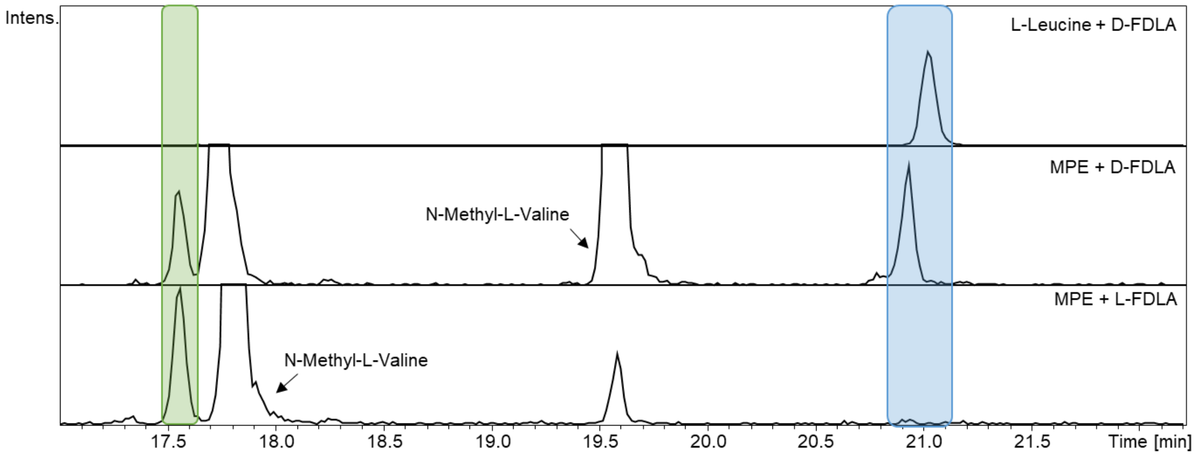

Supplementary Figure 62. Marfey's derivatization of reference L-Leucine with D-FDLA (upper chromatogram) and MP E with both D-FDLA (middle chromatogram) and L-FDLA (lower chromatogram). Retention time comparison represented as EICs; D-L-FDLA/L-D-FDLA highlighted in blue and L-L-FDLA/D-D-FDLA in green.

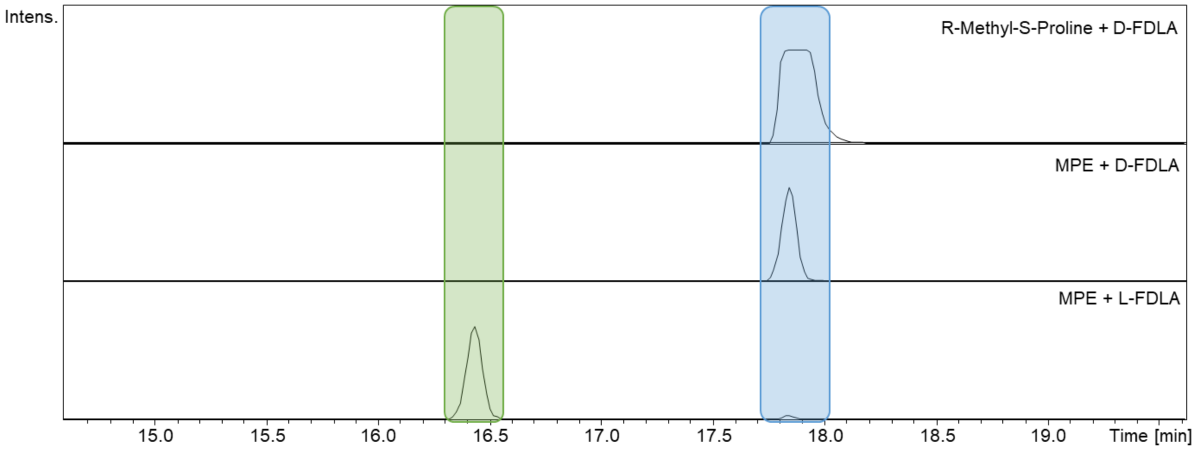

Supplementary Figure 63. Marfey's derivatization of reference R-Methyl-S-Proline (upper chromatogram) with D-FDLA and MPE with both D-FDLA (middle chromatogram) and L-FDLA (lower chromatogram). Retention time comparison represented as EICs; D-L-FDLA/L-D-FDLA highlighted in blue and L-L-FDLA/D-D-FDLA in green.

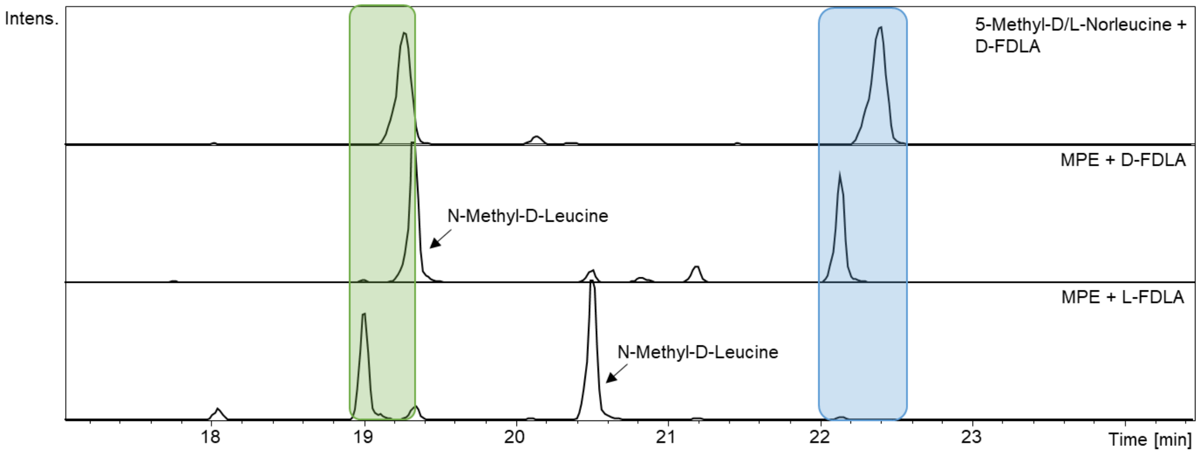

Supplementary Figure 64. Marfey's derivatization of reference 5-Methyl-D/L-Norleucine with D-FDLA (upper chromatogram) and MP E with both D-FDLA (middle chromatogram) and L-FDLA (lower chromatogram). Retention time comparison represented as EICs; D-L-FDLA/L-D-FDLA highlighted in blue and L-L-FDLA/D-D-FDLA in green.

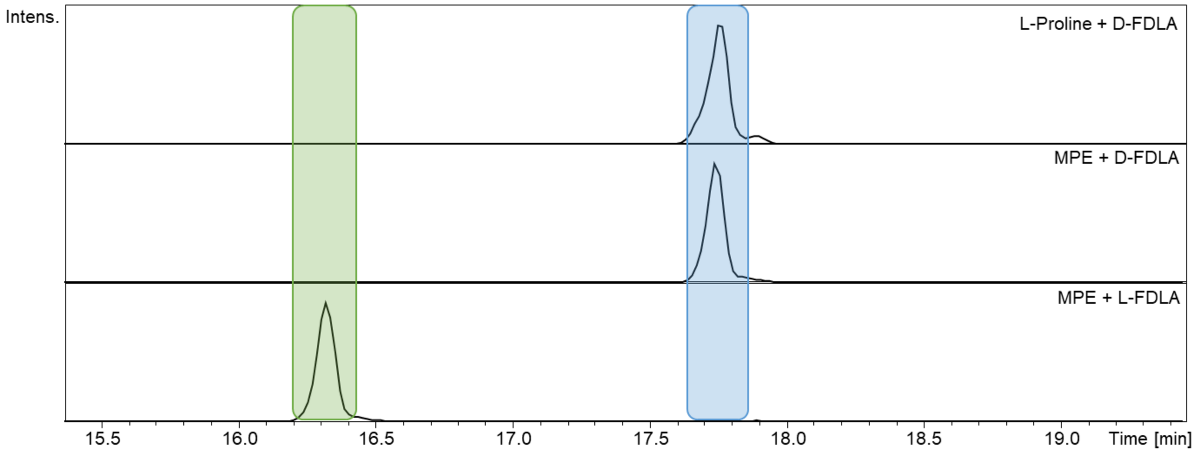

Supplementary Figure 65. Marfey's derivatization of reference L-Proline (upper chromatogram) with D-FDLA and MP E with both D-FDLA (middle chromatogram) and L-FDLA (lower chromatogram). Retention time comparison represented as EICs; D-L-FDLA/L-D-FDLA highlighted in blue and L-L-FDLA/D-D-FDLA in green.

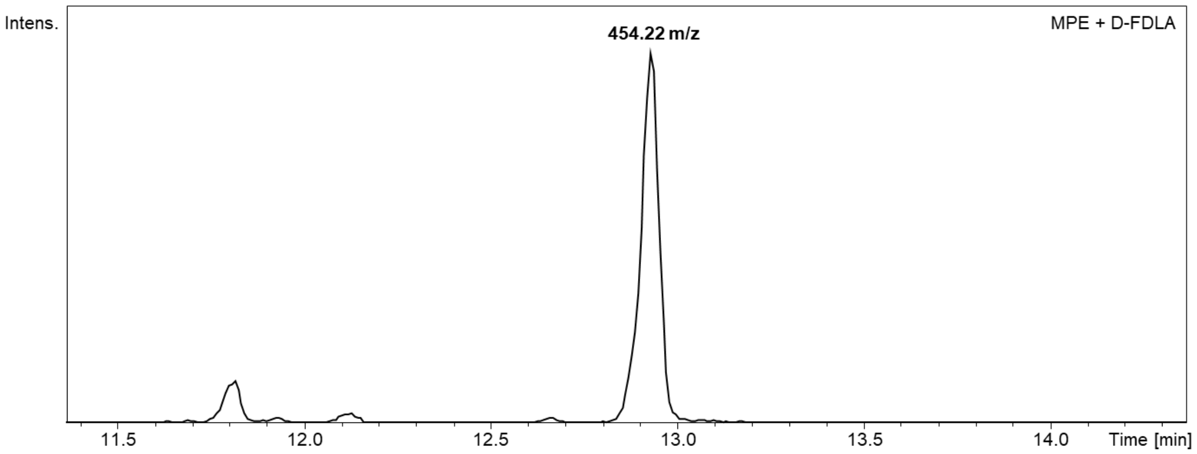

Supplementary Figure 66. Marfey's derivatization of MP E with D-FDLA measured with a more apolar gradient. Represented as EICs (454.22 m/z), no reference N-Methyl-D/L-heptanoic acid was available.

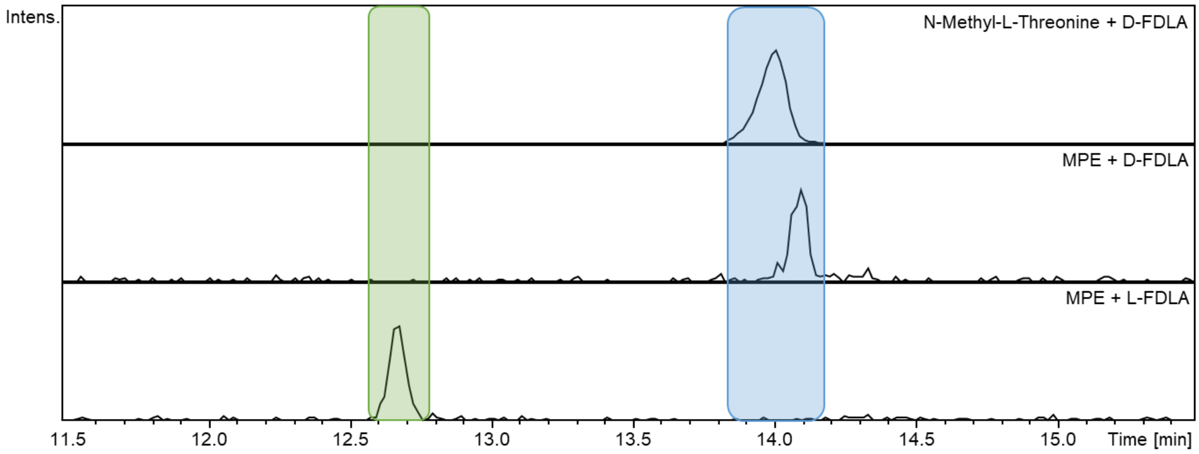

Supplementary Figure 67. Marfey's derivatization of reference N-Methyl-L-Threonine (upper chromatogram) with D-FDLA and MP E with both D-FDLA (middle chromatogram) and L-FDLA (lower chromatogram). Retention time comparison represented as EICs; D-L-FDLA/L-D-FDLA highlighted in blue and L-L-FDLA/D-D-FDLA in green.

Supplementary Tables

Supplementary Table 1. All homologs of GriE and GriF found in biosynthetic gene clusters (BGCs) in all species of the phylum *Actinobacteria* which were used in sequence similarity networks in this study.

Genome_cluster	Type	BGC Class	Cluster_start	Cluster_end	GriE_coords	_GriF_coords
BEWB01000004_c6	1	Betalactone Discrete NRPS	510782	538378	515361, 516149	514204, 515295
BMUX01000001_c1	1	Betalactone Discrete NRPS	837322	865359	841853, 842644	840729, 841832
BMVC01000001_c1	1	Betalactone Discrete NRPS	837128	865165	841659, 842450	840535, 841638
CP034353_c8	1	Betalactone Discrete NRPS	1677831	1705607	1700156, 1700944	1701010, 1702101
CP045547_c9	1	Betalactone Discrete NRPS	1886730	1914486	1909101, 1909889	1909880, 1911046
CP054920_c8	1	Betalactone Discrete NRPS	1645958	1673770	1668319, 1669107	1669173, 1670264
JADWMQ010000101_c0	1	Betalactone Discrete NRPS	0	18359	12908, 13696	13762, 14853
KZ195577_c11	1	Betalactone Discrete NRPS	1705967	1733724	1728291, 1729079	1729145, 1730236
LIXJ01000008_c0	1	Betalactone Discrete NRPS	63603	107240	105408, 106217	104296, 105444
NEVD01000016_c0	1	Betalactone Discrete NRPS	29316	57073	51640, 52428	52494, 53585
NSKH01000004_c5	1	Betalactone Discrete NRPS	359825	439360	394013, 394801	394819, 395904
SOAV01000003_c1	1	Betalactone Discrete NRPS	198965	225241	221022, 221813	221828, 222952
SOPG01000001_c6	1	Betalactone Discrete NRPS	494201	521977	498865, 499653	497711, 498802
WHZR01000001_c6	1	Betalactone Discrete NRPS	1520282	1548047	1542678, 1543466	1543457, 1544623
WXVT01000001_c10	1	Betalactone Discrete NRPS	1928489	1956238	1950835, 1951623	1951689, 1952780
BMWJ01000007_c1	2	NRPS	179756	252843	222308, 223078; 216373, 217131	221154, 222257
JAGINS010000002_c6	2	NRPS	634405	707492	671022, 671780; 676957, 677727	675803, 676906
LWKZ01000003_c1	2	NRPS	174473	266030	250667, 251434	251503, 252606
ML123098_c1	2	NRPS	324460	396020	359429, 360187; 365364, 366134	364210, 365313
RPGY01000005_c0	2	NRPS	2	47993	17337, 18107; 11402, 12160	16183, 17286
CP011522_c32	3	NRPS	5127728	5203944	5145624, 5146388	5146445, 5147533
CP028719_c39	3	NRPS	7065888	7200318	7082988, 7083788	7083809, 7084894
CP032543_c12	3	NRPS	2936423	3009905	2994060, 2994824	2992885, 2993964
CP053189_c16	3	NRPS	1843849	1913056	1894934, 1895698	1893792, 1894877
FMCJ01000051_c0	3	NRPS	0	40355	21558, 22322	20413, 21567
JAEMGU010000001_c42	3	NRPS	6973436	7101656	6991179, 6991979	6992000, 6993085
KZ819167_c22	3	NRPS	4186860	4260348	4201942, 4202706	4202802, 4203881
MTKC01000003_c22	3	NRPS	5066973	5197017	5084779, 5085579	5085600, 5086685
MUNF01000351_c0	3	NRPS	0	25524	13908, 14708	12802, 13887
PDER01000012_c0	3	NRPS	144129	273112	162132, 162932	162953, 164038
PDES01000009_c0	3	NRPS	82384	211160	100387, 101187	101208, 102293
QBHW01000021_c4	3	NRPS	512664	589232	530631, 531395	531491, 532570

QQVZ01000002_c5	3	NRPS	537287	666984	554322, 555122	555143, 556228
WUEJ01000006_c1	3	NRPS	245124	321326	302835, 303599	301660, 302739
WWGH01000001_c12	3	NRPS	2930291	3006795	2988029, 2988793	2986884, 2988038
WWJD01000054_c0	3	NRPS	0	40355	21558, 22322	20413, 21567
JABFHJ010000001_c24	4	NRPS	4725011	4800277	4742055, 4742813	4742875, 4743957
JOBF01000033_c0	5	NRPS	1	203107	168686, 169486	169507, 170598
JOFN01000038_c0	5	NRPS	0	54041	37165, 37965	37986, 39077
AP023408_c28	6	discrete NRPS	5497636	5538330	5533034, 5533825	5531908, 5532921
BBNO01000004_c1	6	discrete NRPS	666527	712869	675796, 676587	676617, 677714
BLIO01000001_c24	6	discrete NRPS	5448132	5494212	5484306, 5485097	5483179, 5484282
CP023691_c25	6	discrete NRPS	3148326	3194488	3157568, 3158359	3158383, 3159486
CP050976_c28	6	discrete NRPS	3295694	3338498	3301651, 3302442	3302466, 3303569
MIGA01000055_c0	6	discrete NRPS	0	31935	21878, 22669	20751, 21854
BMRG01000014_c0	7	NRPS	68083	173432	80007, 80765	78904, 79977
JAGINV010000001_c4	7	NRPS	942060	1039636	1026931, 1027689	1027719, 1028792
CP011497_c20	8	NRPS	5387775	5436495	5412820, 5413611	5413647, 5414729
CP050974_c17	8	NRPS	6243443	6286839	6269120, 6269890	6269947, 6271029
PJNF01000001_c2	8	NRPS	552796	595944	578310, 579080	579137, 580219
CP029078_c12	9	NRPS	1005942	1134891	1045619, 1046410; 1051528, 1052304	1050392, 1051495
CP034687_c34	9	NRPS	7822138	7951207	7910740, 7911531; 7904846, 7905622	7905655, 7906758
FMCI01000220_c0	9	NRPS	6762	55784	42665, 43456; 36767, 37522	37576, 38679
WWIV01000231_c0	9	NRPS	6762	55784	42665, 43456; 36767, 37522	37576, 38679
BMSQ01000002_c1	10	NRPS	413388	489395	463666, 464442	464487, 465578
CP023690_c39	10	NRPS	7364786	7440020	7389515, 7390291	7388379, 7389470
CP030771_c32	10	NRPS	7308085	7382556	7332639, 7333415	7331503, 7332585
CP040916_c31	10	NRPS	7029502	7103198	7054285, 7055061	7053149, 7054240
JACHJD010000006_c2	10	NRPS	465022	541029	489976, 490752	488840, 489931
JOJM01000063_c0	10	NRPS	15070	90797	65116, 65913	65937, 67043
MJAH01000029_c0	11	NRPS	33121	99405	51798, 52580	50653, 51762
RSEG01000038_c0	11	NRPS	0	38440	18982, 19743	19815, 20915
JODE01000022_c0	12	NRPS	50449	179288	160188, 160985	161009, 162097
JODJ01000024_c0	12	NRPS	0	94756	75656, 76453	76477, 77565
RQJC01000010_c0	12	NRPS	6668	231088	78257, 79054	77151, 78233
JABQ01000048_c0	13	NRPS	0	79694	54200, 54997	53070, 54173
RJUU01000001_c11	14	NRPS	4356238	4439789	4372837, 4373631	4373682, 4374776
BCBX01000034_c0	15	NRPS	24102	146170	52008, 52784	52805, 53908
BCBX01000034_c1	15	NRPS	24102	98089	52008, 52784	52805, 53908
CP010407_c10	16	PKS/NRPS	740227	867884	778907, 779716	777795, 778898
JODW01000047_c0	16	PKS/NRPS	0	72267	53174, 53971	53992, 55104
QQVZ01000001_c13	16	PKS/NRPS	2850324	2976622	2917428, 2918231	2918228, 2919346
SSBJ01000014_c0	16	PKS/NRPS	1	177964	22069, 22863	20948, 22075
WBOF01000001_c14	17	PKS/NRPS	4870129	5053211	4960794, 4961555	4959649, 4960734
JAEKDR010000004_c27	18	PKS + discrete NRPS	7235958	7361097	7353495, 7354256	7352326, 7353417

JAEKDS010000004_c3	18	PKS + discrete NRPS	546146	671285	552988, 553749	553827, 554918
JADOUA010000001_c22	20	NRPS	5030741	5128718	5056107, 5056913	5056928, 5058019
BOML01000092_c0	21	NRPS	0	57271	5957, 6745	4827, 5966
CP021978_c30	22	ADEP	7148026	7211204	7165162, 7165965	7165968, 7167092
JACHJK010000013_c5	22	ADEP	263525	302204	288384, 289148	289196, 290311
MK047367_c0	22	ADEP	0	36600	3393, 4196	4199, 5323
BDBI01000010_c0	23	NRPS	0	33909	18719, 19480	19498, 20583
BDBN01000007_c0	23	NRPS	53230	100784	72068, 72829	70959, 72053
BDBR01000033_c0	23	NRPS	77930	116022	110910, 111680	111665, 112774
LJJF01000001_c20	25	NRPS	2318375	2361226	2335655, 2336452	2334540, 2335646
PDCM01000002_c42	26	NRPS	4067389	4163430	4083892, 4084659	4082735, 4083823
KP211414_c0	27	Griselimycin BGC_NRPS	0	66865	56431, 57240	57240, 58412
LLZH01000049_c0	28	Mycoplanecin BGC_NRPS	0	18661	6455, 7231	7240, 8343

Supplementary Table 2. Deduced functions of genes in the mycoplanecins biosynthetic gene cluster (genbank ccession number OR083095) from *Actinoplanes awajinensis* ATCC 33919.

Gene	Size (aa)	Predicted function in MP biosynthesis	Best match protein/organism	Best match accession no. (identity/similarity %)
mypR	375	Self-resistance	DNA polymerase III subunit beta[*Streptomyces sp.* CNH099]	WP_027752940.1 (44/62%)
tnp1	306		putative transposase [*Micromonospora sp.* CNZ280]	NII45199.1 (86/92%)
tnp2	374		IS30 family transposase [*Nonomuraea spiralis*]	WP_229825105.1 (86/92%)
mypM	396	*S*-adenosylmethionine synthetase	methionine adenosyltransferase [*Actinoplanes octamycinicus*]	WP_185045711.1 (96/98%)
mypL	575	N-terminal: isopropylmalate isomerase small subunit<br>C-terminal: aminotransferase	aminotransferase class I/II [*Streptomyces sp.* Root1304]	KQX58119.1 (44/59%)
mypK	537	2-hydroxy-2-isobutylsuccinic acid synthase	2-isopropylmalate synthase [*Streptomyces sp.* MJM8645]	WP_063351482.1 (58/72%)
mypP	396	Oxidase	cytochrome P450 [*Sphaerisporangium rubeum*]	WP_184987000.1(47/63%)
mypA	7621	NRPS (C-A-MT-T-C-A-T-C-A-MT-T-C-A-T-C-A-T-C-A-T-C)[a]	non-ribosomal peptide synthase [*Bradyrhizobium oligotrophicum*]	BAM86977.1 (53/66%)
mypB	3989	NRPS (A-MT-T-C-A-T-C-A-MT-T-E) [a]	nonribosomal peptide synthetase [*Streptomyces muensis*]	AKC91849.1 (52/63%)
mypC	1285	NRPS (C-A-T-TE) [a]	Dimodular nonribosomal peptide synthase [*Streptomyces sp.* ADI98-12]	RPK80042.1 (46/60%)
mypD	77	MbtH	MbtH family protein [*Amycolatopsis xylanica*]	WP_091286165.1 (63/75%)
mypE	262	Homoisoleucine hydroxylase	leucine hydroxylase [*Streptomyces sp.* DSM 40835]	AKC91859.1 (57/75%)
mypF	372	dehydrogenase	alcohol dehydrogenase catalytic domain-containing protein [*Streptomyces sp.* ADI98-12]	WP_124288541.1 (60/74%)
mypG	249	TE	thioesterase [*Streptomyces sp.* NRRL B-24085]	WP_053852351.1 (49/65%)
mypH	632	Radical-SAM methyltransferase	hypothetical protein [*Streptomyces atratus*]	WP_072485841.1 (57/73%)
mypI	83	Acyl carrier protein	acyl carrier protein [*Acholeplasma granularum*]	WP_025725118.1 (49/67%)

mypJ	349	ketosynthase	ketoacyl-ACP synthase III [*Streptomyces sp.* CB02009]	WP_073904150.1 (63/76%)

a. Abbreviations of catalytic nonribosomal peptide synthetase (NRPS) domains: C = condensation domain, A = adenylation domain, MT = methylation domain, T = thiolation domain, also known as peptidyl carrier protein (PCP) domain in NRPS, E = epimerization domain, TE = thioesterase domain.

Supplementary Table 3. Substrate specificity analysis of the 11 adenylation (A) domains from the mycoplanecins megasynthetase

	Specificity-conferring code[a]										Predicted substrate[b]	Incorporated[c]
	235	236	239	278	299	301	322	330	331	517		
A1	D	F	W	S	V	G	I	V	H	K	MeThr	N-MeVal
A2	D	V	Q	F	V	G	H	A	C	K	Pro	MePro/EtPro/PrPro
A3	D	F	W	S	V	G	M	V	H	K	MeThr	N-MeThr
A4	D	A	M	L	V	G	A	I	A	K	Leu	Leu
A5	D	V	Q	Y	V	G	H	A	C	K	Pro	MePro
A6	D	V	M	V	I	G	G	I	A	K	'asc'	Homoleu
A7	D	V	M	V	I	G	G	I	A	K	MeThr	N-MeVal
A8	D	V	Q	Y	V	A	H	V	S	K	Pro	Pro
A9	D	A	M	L	V	G	A	I	A	K	Leu	N-MeLeu/N-Mehomonorleu
A10	D	I	L	Q	I	G	A	I	W	K	Gly	Gly

[a] Residues defined according to Stachelhaus *et al.* and Challis *et al.*; residue numbering corresponds to gramicidin S synthetase A domain numbering (GrsA, PDB-ID: 1AMU)
[b] Retrieved from reports of the applied antiSMASH bacteria version gene cluster analysis. In A7, no single substrates but a group of several amino acids with similar physico-chemical properties were predicted by NRPSPredictor2 as indicated by 'asc' = aromatic side chain amino acids (phe, trp, phg, tyr, bht).
[c] Amino acids incorporated in the mycoplanecins structures.

Supplementary Table 4. Analysis of active site, acyl-transfer and C-O bond formation region residues of MypJ homologues.

	Active site residues (AT activity)			Middle pocket residues		
DpsC[a]	S118	H297	D302	V116	Y165	A329
CerJ[b]	C116	H295	D300	R115	Y164	A327
ZhuH	C121	H257	N288	A120	F171	A320
MypJ	C121	H252	N283	A120	F166	A315

[a] Residue highlighted in yellow represent the conservative site for acyl-transfer region of DpsC.

[b] Residue highlighted in light green represent the conservative site for C-O bond formation region of CerJ.

Supplementary Table 5. Walking primers used for the verification of MP gene cluster.

Name	Sequence of primer
mypA-checkprimer-F	5'-GCTCGCACCACTCGCACAGT-3'
mypA-checkprimer-R	5'-CGCCACGATGCGGGATCGCT-3'
mypA-checkprimer-R2	5'-AAGAAGCGCGCAACCCGTTC-3'

mypA-checkprimer-F3	5’-CCTCCAGGTCAGGAGTGTGA-3’
mypA-checkprimer-R3	5’-CGCGGCTGGGTGAGTTGTTG-3’

Supplementary Table 6. The comparison of bioactivity of MPs and GM against different bacteria.

**Compound**	**MIC [µg/mL]**				
	***S. aureus* Newman**	***B. subtilis* DSM 10**	***E. coli* BW25113**	***C. freundii* DSM 30039**	***A. baumannii* DSM 30008**
Mycoplanecin A	> 64	> 64	> 64	> 64	> 64
Mycoplanecin B	> 64	> 64	> 64	> 64	> 64
Mycoplanecin D	> 64	> 64	> 64	> 64	> 64
Mycoplanecin E	> 64	> 64	> 64	> 64	> 64
Griselimycin	> 64	> 64	> 64	> 64	> 64

Supplementary Table 7. Primers used for protein expression.

Name	Sequence of primer
AaProC-F	TGGTGCCGCGCGGCAGCCATATGACCAAGCACACCGTCGC
AaProC-R	CTCGAGTGCGGCCGCAAGCTTCAGACGGCTTGCGCGGCGA
MypF-F	ATCATATCGAAGGTAGGCATATGATATCGCCGGGCATCGC
MypF-R	TATCTAGACTGCAGGTCGACCTATCGGCCCACGCCGAGCA
MypE-F	TGGTGCCGCGCGGCAGCCATATGCAGTTGTCGGAGCAACA
MypE-R	CTCGAGTGCGGCCGCAAGCTTCACGCGACGGCTCCCTCGA
AaLeuB-F	GCG**CCATGG**CTAGCGTGGCGCGGATCGCGGTA[b]
AaLeuB-R	CGC**AAGCTT**TCAGACGGCGGCGGCGACCCGGT
MypK-F	TGGTGCCGCGCGGCAGCCATATGGCACCAGACGAGCCATT
MypK-R	GGTGCTCGAGTGCGGCCGCACTAGGGCGACGATGTGGCGA
MypL$_C$-F	ATC**GGATCC**ACCACCAAATTTTCGGCGCGCA
MypL$_C$-R	GTG**CTCGAG**CTATATGAACGCGGATATCCGA
MypL$_N$-F	ATA**CATATG**GAGAAGTGGACAGTGCACACCG
MypL$_N$-R	GCC**GATATC**TTACCGACGTGAGACGCGAGGCAGGA
AaLeuC-F	TCC**GAATTC**GGTGGGAGTCACTCCCCAGGGC
AaLeuC-R	CGC**AAGCTT**TCAGAGGTCGGCGGGGGCGGCCA

[a] Homology arm used for Gibson assembly highlighted in red.

[b] Restriction sites used for cloning in bold and underlined.

Supplementary Table 8. X-ray data collection and refinement statistics.

**Structure**	ecDnaN + GM	ecDnaN + CGM	ecDnaN + MP A
PDB-ID:	8CIX	8CIY	8CIZ
**Data collection**			
Beamline	BESSY BL 14.1	SLS X06DA (PXIII)	DESY P11
Wavelength (Å)	0.92	1.00	1.03
Space group	C222	C222	C2
Cell dimensions			
a, *b*, *c* (Å)	83.94, 150.57, 71.59	83.89, 150.71, 71.49	84.14, 151.02, 72.59
α, β, γ (°)	90, 90, 90	90, 90, 90	90, 103.95, 90
Resolution (Å)[a]	71.59 – 1.65 (1.68 – 1.65)	51.86 – 1.50 (1.504 – 1.499)	56.66 – 2.12 (2.115 – 2.118)
R_{merge} (%)[a]	10.5 (118.7)	6.4 (129.3)	7.2 (89.4)
R_{pim} (%)[a]	3.0 (36.6)	1.8 (37.0)	3.0 (35.8)
$I/\sigma I$[a]	17.8 (2.2)	25.9 (2.0)	11.6 (2.2)
Completeness (%)[a]	100.0 (100.0)	100.0 (99.7)	98.9 (99.2)
Redundancy[a]	13.3 (12.6)	13.3 (13.2)	6.8 (7.1)
$CC_{1/2}$ (%)[a]	100.0 (66.4)	100.0 (74.0)	99.9 (78.0)
**Refinement**			
Resolution (Å)	1.65	1.50	2.12
No. reflections	54857	72933	49264
$R_{work/}$ R_{free} (%)	17.53/19.49	16.69/18.07	22.23/26.46
No. atoms	3856	4112	6570
Protein	3343	3449	6327
Ligand/ion	105	112	168
Water	408	551	75
B-factors (Å^2)	27.28	27.17	68.99
Protein	25.92	25.40	68.90
Ligand/ion	28.41	29.70	75.40
Water	36.94	37.71	62.33
R.m.s deviations			
Bond lengths (Å)	0.005	0.005	0.003
Bond angles (º)	0.849	1.015	0.641
Ramachandran statistics (%)			
Favored	98.31	98.32	96.37
Allowed	1.69	1.68	3.63
Outliers	0.00	0.00	0.00
Clashscore (MolProbity)	4.75	5.36	3.82
MolProbity score	1.24	1.60	1.41

[a]Values for the highest resolution shell are shown in parentheses.

Supplementary Table 9. Crystallization conditions

**Co-crystal**	ecDnaN + GM	ecDnaN + CGM	ecDnaN + MP A
Protein concentration	20 mg/ml	20 mg/ml	10 mg/ml
Ligand concentration	10 mM	10 mM	1 mM
DMSO in sample	10 % (v/v)	10 % (v/v)	2 % (v/v)
Temperature	293 K	293 K	293 K
Precipitants/Salts	0.36 M $CaCl_2$	0.64 M $CaCl_2$	0.2 M $CaCl_2$
	0.13 M $MgCl_2$		0.15 M $MgCl_2$
	0.24 M Na Acetate	0.5 M Li Acetate	
	8.3 % (v/v) Glycerol		8.75 % (v/v) Glycerol
	12.2 % (w/v) PEG 3350	9.4 % (w/v) PEG 8000	17.5 % (w/v) PEG 3350
Buffer	0.1 M HEPES/NaOH	0.1 M HEPES/NaOH	0.1 M Tris/HCl
pH	7.7	7.7	9.0
Cryo protectant	10 % (v/v) (2R,3R)-2,3-butanediol.	10 % (v/v) (2R,3R)-2,3-butanediol.	10 % (v/v) (2R,3R)-2,3-butanediol.

Supplementary Table 10. Kinetic data for the enzymatic condensation MypK substrates

	MypK	
Substrate	Km (µM)	Kcat/Km (1/mM/s)
4-methyl-2-ketopentanoate	33.5 ± 17.9	1.63 ± 0.46
3-methyl-2-ketopentanoate	30.5 ± 0.2	0.153 ± 0.03
2-ketohexanoate	31.7 ± 7.3	1.06 ± 0.2

Structure elucidation of mycoplanecins using NMR

1D and 2D experiments as well as high resolution mass spectrometry data revealed the sequence of MP A to be as follows: α-ketobutyrate-(N-Me-Val)-(4-Et-Pro)-(Leu)-(4-Me-Pro)-(5-Me-Norleu)-(N-Me-Val)-(Pro)-(N-Me-Leu)-(Gly)-(N-Me-Thr). Cyclization of the depsipeptide takes place between the C-terminal glycine and the N-methyl-threonine hydroxyl function forming an ester-bond. A detailed description of the NMR analysis is enclosed.

Mycoplanecin A

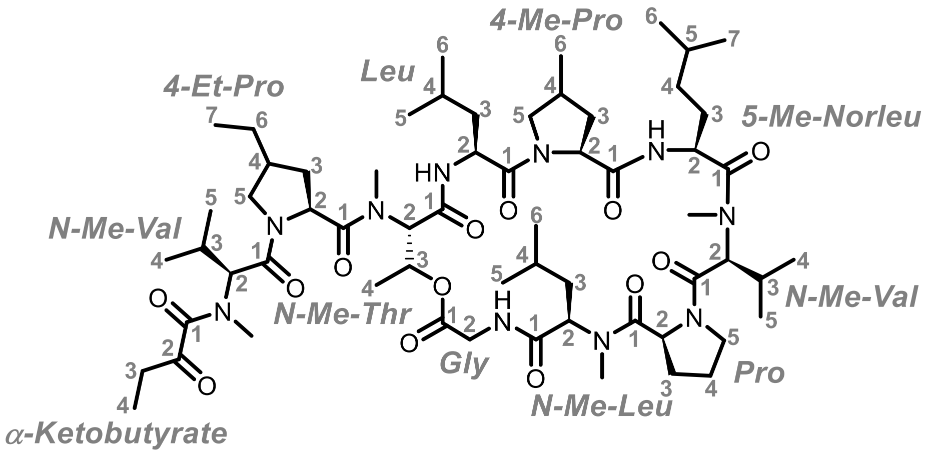

Supplementary Table 11. NMR spectroscopic data of MP A in CDCl3 at 500/125 MHz.

	NMR data in $CDCl_3$				
position	δ_C [ppm]	δ_H [ppm], mult. (*J* [Hz])	COSY correlations	HMBC correlations	TOCSY correlations
α-Keto-butyrate					
1	168.0	-	-	-	-
2	201.7*	-	-	-	-
3	33.5	2.76, m	4	2	4
4	6.8	1.15, t (7.61)	3	2	3
N-Me-Val					
1	167.8	-	-	-	-
2	58.8	4.94, m	3	1,3,4,5,N-Me,1 *α-Ketobutyrate*	3,4,5
3	27.1	2.33, m	2,4,5	1,2,4,5	4,5
4,5	18.9	0.99, d (6.24)	2	2,3	3
N-Me	30.7	2.97*, s	-	1 *α-Ketobutyrate*, 2	-
4-Et-Pro					

1	173.3	-	-	-	-
2	56.4	4.94, m	3	1,3,4,5	3,5,7
3	34.3	2.11,1.82, ddd (12.49, 6.72, 2.34)	2,4	4,5,6	4
4	39.3	2.46, m	3,5	3,5,6,7	3,5
5	52.7	3.97,3.34, dd (10.04,7.84)	4,5	2,3,4,6	3,4,6,7
6	26.2	1.36, m	7	-	3,5,7
7	12.4	0.88, m	6	4,6	6
N-Me-Thr					
1	168.9	-	-	-	-
2	59.9	5.59, d (3.12)	3	1,3,4,N-Me,1 *4-Et-Pro*	3,4
3	69.0	5.92, m	2,4	2,4,1 Gly	2,4
4	17.7	1.35, d (6.33)	3	2,3	2,3
N-Me	34.2	3.13*, s	-	2, 1 *4-Et-Pro* ,2 *4-Et-Pro*	2
Leu					
1	173.5	-	-	-	-
2	50.9	4.38, dd (10.04, 8.30)	3	1,3,4,1 *N-Me-Thr*	3,4,5,6, NH
3	40.1	1.51, 1.28, m	2,4	5,6	2,5,6, NH
4	25.3	1.75, m	5,6	-	-
5,6	23.2	0.93, m	4	3,4,5,6	-
NH	-	6.47, d (7.79)	2	2,3,1 *N-Me-Thr*	2,3,4,5,6
4-Me-Pro					
1	170.5	-	-	-	-
2	60.2	4.59, d (8.16)	3	1,4,5,1 *Leu*	3,5,6
3	33.1	2.56, 1.40, m	2,4	2,4,5,6	2,4,5,6
4	33.6	2.45, m	3,5,6	3,5,6	3,5,6
5	53.3	3.11, 3.51, m	4	4	2,3,4,6
6	16.7	1.08, d (6.33)	4	3,5	2,3,4,5
5-Me-Norleu					
1	174.5	-	-	-	-
2	48.5	4.83, m	3,NH	1,4,1 *4-Me-Pro*	-

3	30.7	1.75, 1.44, m	2,4	1,2,4,5	NH
4	34.5	1.22, 1.04, m	3,5	2,5,6,7	NH
5	27.9	1.52, m	4,6,7	4,6,7	-
6,7	22.3	0.89, m	5	4	NH
NH	-	7.89, s	2	2,1 *4-Me-Pro*	3,4,6,7
N-Me-Val					
1	168.7	-	-	-	-
2	57.6	4.79, m	3	1,3,4, N-Me, 1 *5-Me-Norleu*	4,5
3	28.2	2.26, m	2,5	1,2,4,5	2,5
4	18.8	0.89, m	-	-	2,5
5	18.3	0.75, d (6.69)	3	1,2,3,4	2,3,4
N-Me	30.8	3.30*, s	-	2,1 *5-Me-Norleu*	-
Pro					
1	169.8	-	-	-	-
2	58.6	4.79, m	3	-	3,5
3	31.4	2.32, 1.98, m	2	1,4	2,5
4	21.8	1.87, m	5	2	5
5	46.8	3.70, 3.59, m	4	2,4, 1 *N-Me-Val*	2,3,4
N-Me-Leu					
1	171.5	-	-	-	-
2	54.9	5.17, dd (8.53, 6.05)	3	1,3,4,N-Me,1 *Pro*	3,5,6
3	36.8	1.81, 1.41, m	2,4	1,2,4	2,4,5,6
4	24.5	1.36, m	3,5,6	3	3,5,6
5,6	20.9	0.92, m	4	3,4	2,3,4
N-Me	30.4	2.86*, s	-	2,1 *Pro*	-
Gly					
1	170.6	-	-	-	-
2	42.9	4.25, 4.01, m	NH	1,1 *N-Me-Leu*	NH
NH	-	8.78, dd (6.05, 3.94)	2	1,2,1 *N-Me-Leu*	2

*****compared to chemical shifts determined by Nakajima, deviation of 0.03 ppm in ^{1}H experiment and 1 ppm in ^{13}C experiment [25].

Mycoplanecin B

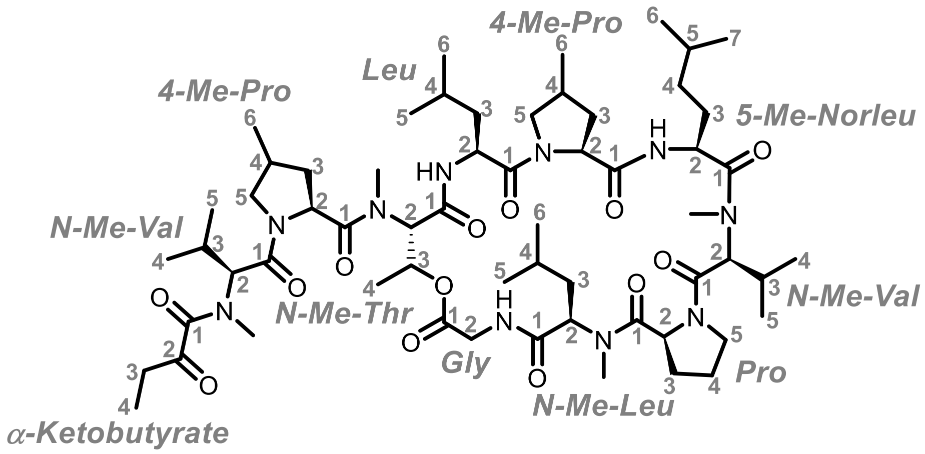

Supplementary Table 12. NMR spectroscopic data of MP B in CDCl3 at 500/125 MHz.

	NMR data in $CDCl_3$				
position	δ_C [ppm]	δ_H [ppm], mult. (J [Hz])	COSY correlations	HMBC correlations	TOCSY correlations
α-Keto-butyrate					
1	168.0	-	-	-	-
2	201.8	-	-	-	-
3	33.5	2.78, dd (7.52,6.51)	4	2,4	4
4	6.8	1.16, t (7.34)	3	2,3	3
N-Me-Val					
1	167.7	-	-	-	-
2	58.9	4.95, m	3	1,3,N-Me	4,5
3	27.1	2.34, m	2,4,5	1	4,5
4,5	18.9	1.01, dd (8.16,6.69)	3	2,3	2,3
N-Me	30.7	2.98, s	-	1 *α-Ketobutyrate*, 2	-
4-Me-Pro					
1	173.2	-	-	-	-
2	56.6	4.97, m	3	1,3,4	3,5,6

3	36.4	2.13,1.82, ddd (12.84, 6.51, 2.75)	2,4	1,2,4,5	2,6
4	32.2	2.64, m	3,5,6	5,6	-
5	54.3	3.96,3.31, dd (9.90,7.52)	4	2,3,4,6, 1 *N-Me-Val*	2,4,6
6	17.5	1.03, m	4	4,5	2,3,4,5
N-Me-Thr					
1	168.9	-	-	-	-
2	59.9	5.60, d (3.30)	3	1,3,4,N-Me,1 *4-Me-Pro*	3,4, N-Me
3	69.0	5.93, m	2,4	2,1 Gly	2,4
4	17.8	1.36, d (6.33)	3	1,2,3	2,3
N-Me	34.1	3.13, s	-	2, 1 *4-Me-Pro*,2 *4-Me-Pro*	2
Leu					
1	173.5	-	-	-	-
2	50.9	4.39, m	3,NH	1,3	NH
3	40.1	1.54, 1.31, m	2	-	5,6,NH
4	25.3	1.78, m	5,6	-	-
5,6	23.3	0.96, m	4	2,3	3,NH
NH	-	6.48, d (7.7)	2	2,3,1 *N-Me-Thr*	2,3,5,6
4-Me-Pro					
1	170.6	-	-	-	-
2	60.3	4.60, d (8.16)	3	1,4,5,1 *Leu*	3,5,6
3	33.1	2.57, 1.43, dd (12.01,6.05)	2,4	2,4,5,6	2,5,6
4	33.6	2.47, m	3,5,6	-	5,6
5	53.3	3.12, 3.52, m	4,6	2,3,4,6	2,3,4,6
6	16.7	1.09, d (6.42)	4,5	3,5	2,3,4,5
5-Me-Norleu					
1	174.5	-	-	-	-
2	48.6	4.84, m	3,NH	1,4,1 *4-Me-Pro*	6,7

3	30.9	1.76, 1.46, m	2,4	1,2,4	NH
4	34.5	1.24, 1.06, m	3	2,5,6,7	NH
5	28.0	1.54, m	-	4,6,7	-
6,7	22.3	0.90, m	-	-	2,NH
NH	-	7.91, d (9.35)	2	2,3,1 *4-Me-Pro*	2,3,4,6,7
N-Me-Val					
1	168.7	-	-	-	-
2	57.6	4.81, m	3	1,3,4, N-Me, 1 *5-Me-Norleu*	4,5
3	28.3	2.28, m	2,4,5	1,2,4,5	5
4	18.9	0.90, m	3	-	2,3,5
5	18.4	0.77, d (6.69)	3	1,2,3,4	2,3,4
N-Me	30.8	3.31, s	-	2,1 *5-Me-Norleu*	-
Pro					
1	169.8	-	-	-	-
2	58.6	4.80, m	3	4	5
3	31.4	2.33, 1.99, m	2	1,5	2,5
4	21.9	1.88, m	5	-	5
5	46.8	3.71, 3.61, m	4	2,3,4, 1 *N-Me-Val*	2,3,4
N-Me-Leu					
1	171.5	-	-	-	-
2	54.9	5.18, dd (8.67, 6.01)	3	1,3,4,N-Me,1 *Pro*	3,5,6
3	36.9	1.82, 1.42, m	2	1,2,4	2,5,6
4	24.6	1.39, m	2	2,3	5,6
5,6	20.9	0.94, m	-	3,4	2,3,4
N-Me	30.4	2.87, s	-	2,1 *Pro*	-
Gly					
1	170.6	-	-	-	-
2	42.9	4.26, 4.02, m	NH	1,1 *N-Me-Leu*	NH

NH	-	8.80, dd (6.24, 3.85)	2	1,2,1 *N-Me-Leu*	2

Mycoplanecin D

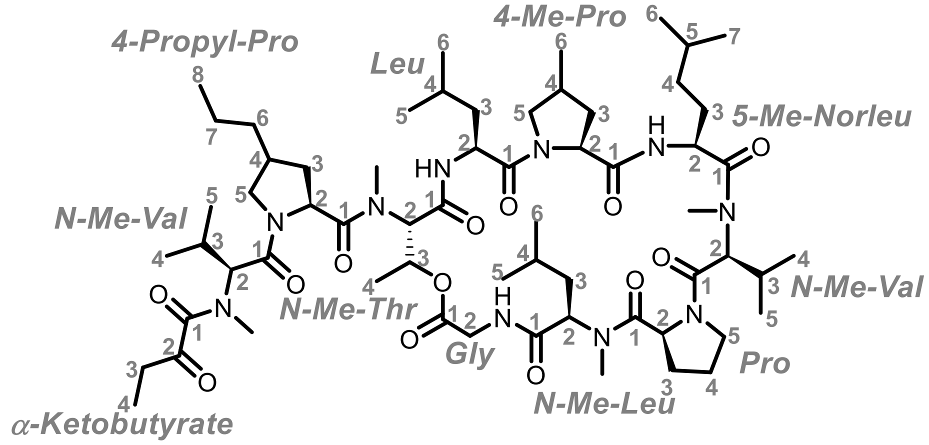

Supplementary Table 13. NMR spectroscopic data of MP D in CDCl3 at 500/125 MHz.

	NMR data in $CDCl_3$				
position	δ_C [ppm]	δ_H [ppm], mult. (*J* [Hz])	COSY correlations	HMBC correlations	TOCSY correlations
α-Keto-butyrate					
1	168.1	-	-	-	-
2	201.8	-	-	-	-
3	33.6	2.78, m	4	-	4
4	6.8	1.15, m	3	2,3	3
N-Me-Val					
1	167.8	-	-	-	-
2	58.6	4.95, m	3	1,3,4,5	4,5
3	27.1	2.32, m	2,4,5	-	-
4,5	18.9	1.00, m	3	3	2
N-Me	30.6	2.99, m	-	1 *α-Ketobutyrate*	-
4-Propyl-Pro					
1	173.5	-	-	-	-
2	56.4	4.95, m	3	-	3
3	34.2	2.13,1.83, m	2,4	1	2,6
4	39.3	2.47, m	3,5	-	-
5	54.2	3.93,3.26, m	4	4	-
6	31.7	1.26, m	-	-	3
7	22.6	1.29, m	8	6,8	-
8	14.1	0.88, m	7	4	-
N-Me-Thr					
1	168.9	-	-	-	-
2	59.9	5.60, m	-	1,3,N-Me,1 *4-Propyl-Pro*	3,4
3	69.0	5.94, m	4	1 Gly	2,4
4	17.8	1.36, m	3	2,3	2,3
N-Me	34.2	3.15, m	-	2, 1 *4-Propyl-*	-

				Pro	
Leu					
1	173.5	-	-	-	-
2	50.9	4.39, m	3,NH	-	NH,3
3	40.1	1.52, 1.31, m	2	-	5,6,NH
4	24.9	1.78, m	5,6	-	-
5,6	23.3	0.95, m	4	3,4	2,3
NH	-	6.49, m	2	-	2,3
4-Me-Pro					
1	170.5	-	-	-	-
2	60.2	4.59, m	3	1,4,1 *Leu*	3,5
3	33.0	2.56, 1.42, dd (12.33,6.1)	2	1,4	2,5,6
4	33.7	2.47, m	5,6	-	-
5	53.3	3.11, 3.52, m	4	4,6	2,3,6
6	16.7	1.10, d (6.60)	5	3	3,5
5-Me-Norleu					
1	174.5	-	-	-	-
2	48.6	4.85, m	3,NH	1	NH
3	30.7	1.74, 1.47, m	2,4,6,7	-	6,7,NH
4	34.5	1.23, 1.04, m	3	5,6,7	NH
5	28.0	1.54, m	-	-	-
6,7	22.3	0.88, m	3	4,5	3,NH
NH	-	7.91, d (9.58)	2	1 *4-Me-Pro*	2,3,4,6,7
N-Me-Val					
1	168.9	-	-	-	-
2	57.6	4.82, m	3	1,3,5, N-Me, 1 *5-Me-Norleu*	4,5
3	28.3	2.26, m	2,4,5	-	5
4	18.9	0.89, m	3	2,5	2,3,5
5	18.4	0.77, m	3	2,3	2,3,4
N-Me	30.9	3.31, s	-	2,1 *5-Me-Norleu*	-
Pro					
1	169.9	-	-	-	-
2	58.5	4.80, m	3	3,4	3,4,5
3	31.4	2.31, 1.98, m	2	-	2
4	21.8	1.88, m	5	-	2,5
5	46.8	3.72, 3.61, m	4	3,4	2,4
N-Me-Leu					
1	171.5	-	-	-	-
2	54.9	5.18, m	3	1,3,4,N-Me,1 *Pro*	3,5,6
3	36.9	1.82, 1.42, m	2	1,2,4	2,5,6
4	24.6	1.37, m	5,6	-	-
5,6	20.9	0.93, m	4	3,4	2,3
N-Me	30.4	2.87, m	-	2,1 *Pro*	-

Gly					
1	170.5	-	-	-	-
2	42.9	4.28, 4.04, m	NH	1,1 *N-Me-Leu*	NH
NH	-	8.80, m	2	2	2

Mycoplanecin E

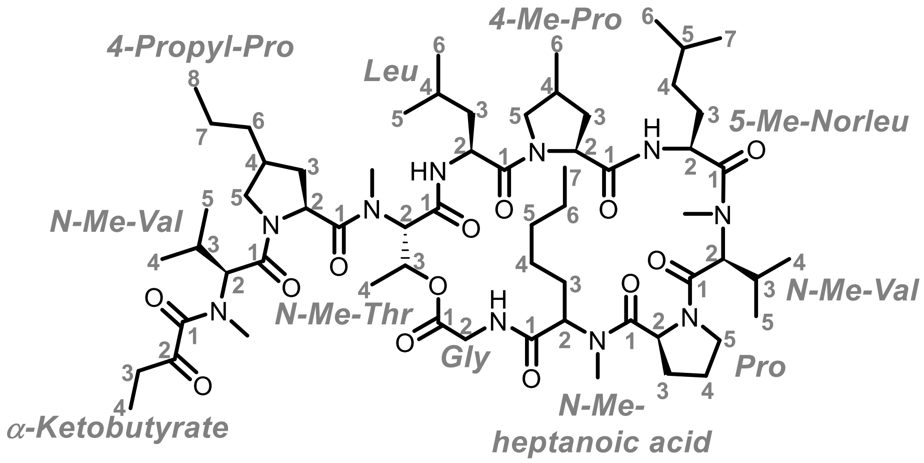

Supplementary Table 13. NMR spectroscopic data of MP E in CDCl3 at 700/175 MHz.

	NMR data in $CDCl_3$			
position	δ_C [ppm]	δ_H [ppm], mult. (*J* [Hz])	COSY correlations	HMBC correlations
α-Keto-butyrate				
1	168.1	-	-	-
2	201.8	-	-	-
3	33.6	2.78, m	4	2,4
4	6.8	1.15, m	3	2,3
N-Me-Val				
1	167.8	-	-	-
2	56.8	4.98, m	3	3
3	27.1	2.34, m	2,4,5	-
4,5	18.8	0.91, m	3	2,3
N-Me	30.8	2.98, m	-	1 *α-Ketobutyrate*
4-Propyl-Pro				
1	173.4	-	-	-
2	58.9	4.97, m	3	1 *4-Propyl-Pro,*4, 1 *N-Me-Val*
3	33.0	2.09,1.84, m	-	1,5
4	39.2	2.46, m	-	-
5	51.7	4.00,3.35, m	-	-
6	31.9	1.27, m	-	-
7	22.7	1.30, m	8	-
8	14.1	0.89, m	7	6,7
N-Me-Thr				
1	168.9	-	-	-
2	59.9	5.60, m	3	1,N-Me,1 *4-Propyl-Pro*
3	69.1	5.93, m	2,4	1 Gly

4	17.7	1.35, m	3	2,3
N-Me	34.1	3.14, m	-	2, 1 *4-Propyl-Pro*
Leu				
1	173.4	-	-	-
2	50.9	4.40, m	3,NH	1 *4-Me-Pro*,3,4
3	40.1	1.53, 1.32, m	2,4	-
4	25.4	1.78, m	3,5,6	-
5,6	21.0	0.93, m	4	-
NH	-	6.49, m	2	1 *N-Me-Thr*
4-Me-Pro				
1	170.3	-	-	-
2	60.3	4.60, br d (8.33)	3	1,5,1 *Leu*
3	33.0	1.42, m	2,4	-
4	33.6	2.47, m	3,5,6	-
5	53.3	3.12, 3.52, m	4	2,6
6	16.7	1.09, br d (6.73)	4	5
5-Me-Norleu				
1	174.4	-	-	-
2	48.7	4.85, m	3,NH	1,3,4,1 *4-Me-Pro*
3	30.8	1.76, 1.46, m	2,4	1 *4-Me-Pro*
4	34.6	1.24, 1.06, m	3	-
5	28.0	1.53, m	6,7	4
6,7	22.7	0.88, m	5	4
NH	-	7.89, m	5	1 *4-Me-Pro*
N-Me-Val				
1	168.9	-	-	-
2	57.4	4.81, m	3	1,3, 1 *5-Me-Norleu*
3	28.2	2.27, m	2,4,5	-
4,5	18.4	0.77, m	3	2,3
N-Me	30.8	3.31, s	-	2,1 *5-Me-Norleu*
Pro				
1	169.8	-	-	-
2	58.9	4.81, m	3	1,3,4
3	31.4	2.33, 1.99, m	2,4	-
4	21.9	1.87, m	3,5	-
5	46.8	3.71, 3.61, m	4	-
N-Me-heptanoic acid				
1	171.5	-	-	-
2	55.0	5.18, m	3	1,3,N-Me,1 *Pro*
3	37.0	1.82, 1.42, m	2	1,2
4	38.9	1.16, m	5	-
5	27.3	1.26, m	4	4
6	24.9	1.64, m	-	-
7	14.1	0.90, m	-	-
N-Me	30.4	2.87, m	-	4

Gly				
1	170.6	-	-	-
2	42.9	4.25, 4.04, m	NH	1,1 *N-Me-Leu*
NH	-	8.79, m	2	-

References

1. Lukat, P. et al. Biosynthesis of methyl-proline containing griselimycins, natural products with anti-tuberculosis activity. *Chem. Sci.* 8, 7521-7527 (2017).
2. Wittmann, M., Linne, U., Pohlmann, V. & Marahiel, M.A. Role of DptE and DptF in the lipidation reaction of daptomycin. *FEBS J.* 275, 5343-5354 (2008).
3. Kraas, F.I., Helmetag, V., Wittmann, M., Strieker, M. & Marahiel, M.A. Functional dissection of surfactin synthetase initiation module reveals insights into the mechanism of lipoinitiation. *Chem. Biol.* 17, 872-880 (2010).
4. Imker, H.J., Krahn, D., Clerc, J., Kaiser, M. & Walsh, C.T. N-acylation during glidobactin biosynthesis by the tridomain nonribosomal peptide synthetase module GlbF. *Chem. Biol.* 17, 1077-1083 (2010).
5. Lewis, R.A. et al. Active site modification of the β-ketoacyl-ACP synthase FabF3 of Streptomyces coelicolor affects the fatty acid chain length of the CDA lipopeptides. *Chem. Commun. (Camb)* 47, 1860-1862 (2011).
6. Bretschneider, T. et al. A ketosynthase homolog uses malonyl units to form esters in cervimycin biosynthesis. *Nat. Chem. Biol.* 8, 154-161 (2011).
7. Jackson, D.R. et al. Structural and functional studies of the daunorubicin priming ketosynthase DpsC. *ACS Chem. Biol.* 13, 141-151 (2018).
8. Pan, H. et al. Crystal structure of the priming beta-ketosynthase from the R1128 polyketide biosynthetic pathway. *Structure*. 10, 1559-1568 (2002).
9. Hitchman, T.S., Crosby, J., Byrom, K.J., Cox, R.J. & Simpson, T.J. Catalytic self-acylation of type II polyketide synthase acyl carrier proteins. *Chem. Biol.* 5, 35-47 (1998).
10. Misra, A., Sharma, S.K., Surolia, N. & Surolia, A. Self-acylation properties of type II fatty acid biosynthesis acyl carrier protein. *Chem. Biol.* 14, 775-783 (2007).
11. Sievers, F. et al. Fast, scalable generation of high-quality protein multiple sequence alignments using Clustal Omega. *Mol. Syst. Biol.* 7, 539 (2011).
12. Robert, X. & Gouet, P. Deciphering key features in protein structures with the new ENDscript server. *Nucleic. Acids. Res.* 42, W320-324 (2014).
13. Oakley, A.J. et al. Flexibility revealed by the 1.85 A crystal structure of the beta sliding-clamp subunit of Escherichia coli DNA polymerase III. *Acta. Crystallogr. D. Biol. Crystallogr.* 59, 1192-1199 (2003).
14. Yin, Z., Kelso, M.J., Beck, J.L. & Oakley, A.J. Structural and thermodynamic dissection of linear motif recognition by the *E. coli* sliding clamp. *J. Med. Chem.* 56, 8665-8673 (2013).
15. Yin, Z. et al. Bacterial sliding clamp inhibitors that mimic the sequential binding mechanism of endogenous linear motifs. *J. Med. Chem.* 58, 4693-4702 (2015).
16. Kling, A. et al. Antibiotics. Targeting DnaN for tuberculosis therapy using novel griselimycins. *Science* 348, 1106-1112 (2015).
17. Gui, W.J. et al. Crystal structure of DNA polymerase III β sliding clamp from *Mycobacterium tuberculosis*. *Biochem. Biophys. Res. Commun.* 405, 272-277 (2011).
18. Kukshal, V. et al. M. tuberculosis sliding β-clamp does not interact directly with the NAD+-dependent DNA ligase. *PLoS One* 7, e35702 (2012).
19. Wolff, P. et al. Differential modes of peptide binding onto replicative sliding clamps from various bacterial origins. *J. Med. Chem.* 57, 7565-7576 (2014).
20. Steiningerova, L. & Kamenik, Z. Different reaction specificities of F420H2-dependent reductases facilitate pyrrolobenzodiazepines and lincomycin to fit their biological targets. *J. Am. Chem. Soc.* 142, 3440-3448 (2020).
21. Junne, T. et al. Decatransin, a new natural product inhibiting protein translocation at the Sec61/SecYEG translocon. *J. Cell. Sci.* 128, 1217-1229 (2015).

22. Shiba, T. & Mukunoki, Y. The total structure of the antibiotic longicatenamycin. *J. Antibiot.* 28, 561-566 (1975).
23. Son, S. & Hong, Y.S. Genomics-driven discovery of chlorinated cyclic hexapeptides ulleungmycins A and B from a Streptomyces species. *J. Nat. Prod.* 80, 3025-3031 (2017).
24. Wang, S.A., Lin, C.I. & Zhang, J. Studies of lincosamide formation complete the biosynthetic pathway for lincomycin A. *Proc. Natl. Acad. Sci.* 117, 24794-24801 (2020).
25. Nakajima, M. et al. Mycoplanecins, novel antimycobacterial antibiotics from *Actinoplanes awajinensis subsp. mycoplanecinus subsp.* nov. III. Structural determination of mycoplanecin A. *J. Antibiot.* 36, 967-975 (1983).
