# Supplementary material for: Elucidation of unusual biosynthesis and DnaN-targeting mode of action of potent anti-tuberculosis antibiotics Mycoplanecins
Source: Nat Commun. 2024 Jan 26;15:791. doi: 10.1038/s41467-024-44953-5 (PMC10817943; doi:10.1038/s41467-024-44953-5)
Supplement: Supplementary file 2 — Reporting Summary [file 41467_2024_44953_MOESM2_ESM.pdf]

nature portfolio

Corresponding author(s): Rolf Müller

Last updated by author(s): Oct 27, 2023

Reporting Summary

Nature Portfolio wishes to improve the reproducibility of the work that we publish. This form provides structure for consistency and transparency in reporting. For further information on Nature Portfolio policies, see our Editorial Policies and the Editorial Policy Checklist.

Statistics

For all statistical analyses, confirm that the following items are present in the figure legend, table legend, main text, or Methods section.

n/a	Confirmed	
☐	☒	The exact sample size (*n*) for each experimental group/condition, given as a discrete number and unit of measurement
☐	☒	A statement on whether measurements were taken from distinct samples or whether the same sample was measured repeatedly
☒	☐	The statistical test(s) used AND whether they are one- or two-sided<br>*Only common tests should be described solely by name; describe more complex techniques in the Methods section.*
☒	☐	A description of all covariates tested
☒	☐	A description of any assumptions or corrections, such as tests of normality and adjustment for multiple comparisons
☐	☒	A full description of the statistical parameters including central tendency (e.g. means) or other basic estimates (e.g. regression coefficient) AND variation (e.g. standard deviation) or associated estimates of uncertainty (e.g. confidence intervals)
☒	☐	For null hypothesis testing, the test statistic (e.g. *F*, *t*, *r*) with confidence intervals, effect sizes, degrees of freedom and *P* value noted<br>*Give P values as exact values whenever suitable.*
☒	☐	For Bayesian analysis, information on the choice of priors and Markov chain Monte Carlo settings
☒	☐	For hierarchical and complex designs, identification of the appropriate level for tests and full reporting of outcomes
☒	☐	Estimates of effect sizes (e.g. Cohen's *d*, Pearson's *r*), indicating how they were calculated

Our web collection on statistics for biologists contains articles on many of the points above.

Software and code

Policy information about availability of computer code

Data collection	Bruker TopSpin 4
Data analysis	BLAST 2.9.0, antiSMASH 5.1.2, MAFFT 7.450, Cytoscape 3.9.1, ACD/Labs 2021.2.0, Bruker Compass DataAnalysis V4.4, autoPROC (Global Phasing) 1.0.5, XDS Jan 10, 2022, CCP4 8.0.006, Pointless 1.12.14, Aimless 0.7.9, Phenix 1.20.1-4487, Phaser 2.8.3, Phenix.Refine 1.20.1-4487, Coot 0.9.6, PyMOL (Schrödinger) 2.4.0, Maestro (Schrödinger) 12.6.149

For manuscripts utilizing custom algorithms or software that are central to the research but not yet described in published literature, software must be made available to editors and reviewers. We strongly encourage code deposition in a community repository (e.g. GitHub). See the Nature Portfolio guidelines for submitting code & software for further information.

Data

Policy information about availability of data

All manuscripts must include a data availability statement. This statement should provide the following information, where applicable:

- Accession codes, unique identifiers, or web links for publicly available datasets
- A description of any restrictions on data availability
- For clinical datasets or third party data, please ensure that the statement adheres to our policy

The mycoplanecin biosynthetic gene cluster sequence has been deposited in GenBank under accession number OR083095. The protein crystal structure data have

been deposited in the Protein Data Bank under PDB IDs 8CIX, 8CIY, and 8CIZ. All data that support the findings of this study are available in the main text and the supplementary information.

Research involving human participants, their data, or biological material

Policy information about studies with human participants or human data. See also policy information about sex, gender (identity/presentation), and sexual orientation and race, ethnicity and racism.

Reporting on sex and gender	n/a
Reporting on race, ethnicity, or other socially relevant groupings	n/a
Population characteristics	n/a
Recruitment	n/a
Ethics oversight	n/a

Note that full information on the approval of the study protocol must also be provided in the manuscript.

Field-specific reporting

Please select the one below that is the best fit for your research. If you are not sure, read the appropriate sections before making your selection.

☒ Life sciences ☐ Behavioural & social sciences ☐ Ecological, evolutionary & environmental sciences

For a reference copy of the document with all sections, see nature.com/documents/nr-reporting-summary-flat.pdf

Life sciences study design

All studies must disclose on these points even when the disclosure is negative.

Sample size	n/a, because our study does not entail any sample collection
Data exclusions	No data were excluded.
Replication	For evaluating the binding affinity to M. smegmatis DnaN using MST, all compounds were titrated in triplicates in an appropriate concentration range for proper dose-response curves with 16 serial dilution steps.
Randomization	n/a, because no clinical trial or patients' data is involved in this study
Blinding	n/a, because no clinical trial or patients' data is involved in this study

Reporting for specific materials, systems and methods

We require information from authors about some types of materials, experimental systems and methods used in many studies. Here, indicate whether each material, system or method listed is relevant to your study. If you are not sure if a list item applies to your research, read the appropriate section before selecting a response.

Materials & experimental systems

n/a	Involved in the study	
☒	☐	Antibodies
☐	☒	Eukaryotic cell lines
☒	☐	Palaeontology and archaeology
☒	☐	Animals and other organisms
☒	☐	Clinical data
☒	☐	Dual use research of concern
☒	☐	Plants

Methods

n/a	Involved in the study	
☒	☐	ChIP-seq
☒	☐	Flow cytometry
☒	☐	MRI-based neuroimaging

Eukaryotic cell lines

Policy information about cell lines and Sex and Gender in Research

Cell line source(s)	The CHO-K1 cell line was ordered from ATCC. CHO-K1 cell line was derived as a subclone from the parental CHO cell line, which was initiated from a biopsy of an ovary of an adult, female Chinese hamster in 1957.
Authentication	The CHO-K1 cell line was ordered from ATCC. This cell line was not authenticated in our lab.
Mycoplasma contamination	Mycoplasma contamination was not detected.
Commonly misidentified lines (See ICLAC register)	No commonly misidentified cell lines were used in the study.
